# Proceedings of the International Cancer Imaging Society (ICIS) 16th Annual Teaching Course: Glasgow, UK. 3–5 October 2016

**DOI:** 10.1186/s40644-016-0079-z

**Published:** 2016-10-03

**Authors:** Dow-Mu Koh, Sue Creviston Kaste, Sarah J. Vinnicombe, Giovanni Morana, Andrea Rossi, Christian J. Herold, Theresa C. McLoud, Kirk A. Frey, Bernhard Gebauer, Derek Roebuck, Jurgen J. Fütterer, Alexander J. Towbin, Thierry A. G. Huisman, Anne M. J. B. Smets, Giovanni Morana, Jeong Min Lee, Hersh Chandarana, Marius E. Mayerhoefer, Markus Raderer, Alexander Haug, Matthias Eiber, Bernhard Gebauer, Andrea Rockall, Aslam Sohaib, Victoria S Warbey, Hebert Alberto Vargas, Dow-Mu Koh, Jay P. Heiken, Isaac R. Francis, Mahmoud M. Al-Hawary, Ravi K. Kaza, Giovanni Morana, Mirko D’Onofrio, Harriet C. Thoeny, Ann D. King, Giovanni Morana, Arnoldo Piccardo, Maria Luisa Garrè, Andrea Rossi, Hebert Alberto Vargas, Theresa C. McLoud, Nick Reed, Carlos Rodriguez-Galindo, Hersh Chandarana, Hebert Alberto Vargas, Isaac R. Francis, Ashish P. Wasnik, Stefan Diederich, Jurgen J. Fütterer, Wim J. G. Oyen, Cheng Lee Chaw, Nicholas van As, Igor Vieira, Frederik De Keyzer, Elleke Dresen, Sileny Han, Ignace Vergote, Philippe Moerman, Frederic Amant, Michel Koole, Vincent Vandecaveye, R. Dresen, S. De Vuysere, F. De Keyzer, E. Van Cutsem, A. D’Hoore, A. Wolthuis, V. Vandecaveye, P. Pricolo, S. Alessi, P. Summers, E. Tagliabue, G. Petralia, C. Pfannenberg, B. Gückel, S. C. Schüle, A. C. Müller, S. Kaufmann, N. Schwenzer, M. Reimold, C. la Fougere, K. Nikolaou, P. Martus, G. J. Cook, G. K. Azad, B. P. Taylor, M. Siddique, J. John, J. Mansi, M. Harries, V. Goh, S. Seth, R. Burgul, A. Seth, S. Waugh, N. Muhammad Gowdh, C. Purdie, A. Evans, E. Crowe, A. Thompson, S. Vinnicombe, F. Arfeen, T. Campion, E. Goldstraw, M. D’Onofrio, V. Ciaravino, S. Crosara, R. De Robertis, R. Pozzi Mucelli, M. Uhrig, D. Simons, H. Schlemmer, Kate Downey, S. Murdoch, A. S. Al-adhami, S. Viswanathan, S. Smith, P. Jennings, D. Bowers, R. Soomal, S. Smith, P. Jennings, D. Bowers, R. Soomal, T. M. Mutala, A. O. Odhiambo, N. Harish, P. Pricolo, S. Alessi, P. Summers, E. Tagliabue, G. Petralia, M. Hall, M. Sproule, S. Sheridan, K. Y. Thein, C. H. Tan, Y. L. Thian, C. M. Ho, S. De Luca, C. Carrera, V. Blanchet, L. Alarcón, E. Eyheremnedy, B. K. Choudhury, K. Bujarbarua, G. Barman, G. J. Cook, E. Lovat, M. Siddique, V. Goh, R. Ferner, V. S. Warbey, L. Potti, B. Kaye, A. Beattie, K. Dutton, A. A. Seth, F. Constantinidis, H. Dobson, A. A. Seth, F. Constantinidis, H. Dobson, R. Bradley, G. Bozas, G. Avery, A. Stephens, A. Maraveyas, S. Bhuva, C. A. Johnson, M. Subesinghe, N. Taylor, L. E. Quint, R. M. Reddy, G. P. Kalemkerian, G. González Zapico, E. Gainza Jauregui, R. Álvarez Francisco, S. Ibáñez Alonso, I. Tavera Bahillo, L. Múgica Álvarez, O. Francies, R. Wheeler, L. Childs, A. Adams, A. Sahdev, S. E. De Luca, M. E. Casalini Vañek, M. D. Pascuzzi, T. Gillanders, P. M. Ramos, E. P. Eyheremendy, C. Stove, M. Digby, M. Nazar, M. Wirtz, M. D. Pascuzzi, F. Troncoso, F. Saguier, E. P. Eyheremendy, D. J. Quint, L. Dang, M. Carlson, S. Leber, F. Silverstein, R. Rueben, S. Viswanathan, B. Nazir, T. H. Teo, J. B. Khoo, K. Sharma, N. Gupta, B. Mathew, T. Jeyakumar, K. Harkins, K. Sharma, B. Mathew, N. Gupta, T. Jeyakumar, S. Joshua, D. Christodoulou, S. Gourtsoyianni, A. Jacques, N. Griffin, V. Goh, C. A. Johnson, J. Lee, J. A. Goodfellow, A. S. Al-adhami, S. Viswanathan, R. Bradley, R. Bradley, A. Yong, S. Jenkins, G. Joseph, S. Bhuva, K. Partington, C. A. Johnson, S. Bhuva, M. Subesinghe, N. Taylor, C. Carrera, A. Zanfardini, S. De Luca, L. Alarcón, V. Blanchet, E. P. Eyheremendy, K. Cavanagh, E. Lau

**Affiliations:** 1 Royal Marsden Hospital, Sutton, Surrey SM2 5PT UK; 2 Department of Diagnostic Imaging, St. Jude Children’s Research Hospital, 262 Danny Thomas Place, MSN #220, Memphis, TN 38105 USA; 3 Department of Oncology, St. Jude Children’s Research Hospital, 262 Danny Thomas Place, Memphis, TN 38105 USA; 4 Department of Radiology, University of Tennessee Health Science Center, 920 Madison Ave, Memphis, TN 38103 USA; 5 Division of Cancer Research, University of Dundee, Ninewells Hospital and Medical School, Dundee, Angus DD1 9SY UK; 6 Neuroradiology Unit, Istituto Giannina Gaslini, Genoa, GE 16145 Italy; 7 Department of Biomedical Imaging and Image Guided Therapy, Medical University of Vienna / Vienna General Hospital, 1090 Vienna, Austria; 8 Department of Radiology, Massachusetts General Hospital, Harvard Medical School, 55 Fruit Street, Boston, MA 02114 USA; 9 Division of Nuclear Medicine and Molecular Imaging, Department of Radiology, The University of Michigan, Ann Arbor, MI 48109-5028 USA; 10 Department of Radiology, Charité, Campus Virchow-Klinikum, Augustenburger Platz 1, 13353 Berlin, Germany; 11 Department of Radiology, Great Ormond Street Hospital, London, WC1N 3JH UK; 12 Department of Radiology and Nuclear Medicine, Radboud University Medical Center, Nijmegen, 6500HB The Netherlands; 13 Department of Radiology, Cincinnati Children’s Hospital, 3333 Burnet Avenue, MLC 5031, Cincinnati, OH 45229 USA; 14 Radiology, Pediatrics, Neurology and Neurosurgery, Department of Imaging and Imaging Science, Johns Hopkins Bayview, 4940 Eastern Avenue, Baltimore, MD 21224 USA; 15 Department of Radiology, Academic Medical Center, University of Amsterdam, Amsterdam, The Netherlands; 16 Radiological Department, General Hospital Ca’ Foncello, Treviso, Italy; 17 Department of Radiology, Seoul National University Hospital, Seoul, Korea; 18 Department of Radiology, New York University School of Medicine, New York, 10016 USA; 19 Department of Biomedical Imaging and Image-guided Therapy, Medical University of Vienna, Vienna, 1090 Austria; 20 Department of Internal Medicine I, Medical University of Vienna, Medical University of Vienna, Vienna, 1090 Austria; 21 Department of Nuclear Medicine, Klinikum rechts der Isar, Technical University Munich, Munich, Germany; 22 Department of Radiology, Charité, Campus Virchow-Klinikum, Augustenburger Platz 1, 13353 Berlin, Germany; 23 Radiology Department, Hammersmith Hospital, London, W12 0HS UK; 24 The Royal Marsden NHS Foundation Trust, Sutton London, UK; 25 Division of Imaging Sciences and Biomechanical Engineering, King’s College London and Guy’s & St Thomas’ PET Centre, King’s College London, London, SE1 7EH UK; 26 Department of Radiology, Memorial Sloan Kettering Cancer Center, New York, NY USA; 27 Royal Marsden Hospital, Sutton, Surrey SM2 5PT UK; 28 Mallinckrodt Institute of Radiology, Washington University School of Medicine, St. Louis, MO 63110 USA; 29 Department of Radiology, University of Michigan Health System, Ann Arbor, Michigan USA; 30 Radiological Department, General Hospital Ca’ Foncello, Treviso, Italy; 31 Department of Radiology, University Hospital G.B. Rossi, Piazzale L.A. Scuro 10, 37134 Verona, Italy; 32 University of Verona, Verona, Italy; 33 Department of Radiology, Neuroradiology and Nuclear Medicine, Inselspital, University of Bern, Bern, Switzerland; 34 Department of Imaging and Interventional Radiology, Chinese University of Hong Kong, Hong Kong, China; 35 Neuroradiology Unit, Istituto Giannina Gaslini, Genoa, GE, 16145 Italy; 36 Nuclear Medicine Unit, Ospedali Galliera, Genoa, GE, 16128 Italy; 37 Neuro-oncology Unit, Istituto Giannina Gaslini, Genoa, GE, 16145 Italy; 38 Department of Radiology, Memorial Sloan Kettering Cancer Center, New York, NY USA; 39 Department of Radiology, Massachusetts General Hospital, Harvard Medical School, 55 Fruit Street, Boston, MA 02114 USA; 40 Beatson Oncology Centre, Glasgow, UK; 41 Department of Global Pediatric Medicine, St. Jude Children’s Research Hospital, Memphis, TN 38105 USA; 42 Department of Radiology, New York University School of Medicine, New York, 10016 USA; 43 Department of Radiology, Memorial Sloan Kettering Cancer Center, New York, NY USA; 44 Department of Radiology, University of Michigan Health System, Ann Arbor, Michigan USA; 45 Department of Diagnostic and Interventional Radiology, Marien Hospital, 40479 Düsseldorf, Germany; 46 Department of Radiology and Nuclear Medicine, Radboud University Medical Center, Nijmegen, 6500HB The Netherlands; 47 The Institute of Cancer Research and The Royal Marsden NHS Foundation Trust, London, UK; 48 Department of Clinical Oncology, Royal Marsden Hospital NHS Foundation Trust and the Institute of Cancer Research, London, UK; 49 Department of Nuclear Medicine, University Hospitals Leuven, Leuven, Belgium; 50 Department of Radiology, University Hospitals Leuven, Leuven, Belgium; 51 Department of Obstretics and Gynaecology, University Hospitals Leuven, Leuven, Belgium; 52 Department of Pathology, University Hospitals Leuven, Leuven, Belgium; 53 Center Gynaecological Oncology Amsterdam (CGOA), Antoni van Leeuwenhoek, Amsterdam, The Netherlands; 54 Department of Radiology, Leuven Cancer Institute, University Hospitals Leuven, KU Leuven, Leuven, Belgium; 55 Department of Oncology, Leuven Cancer Institute, University Hospitals Leuven, KU Leuven, Leuven, Belgium; 56 Department of Abdominal Surgery, Leuven Cancer Institute, University Hospitals Leuven, KU Leuven, Leuven, Belgium; 57 European Institute of Oncology, Milano, Italy; 58 Department of Diagnostic and Interventional Radiology, University Tuebingen, Tübingen, Germany; 59 Clinic of Radiation Oncology, University Tuebingen, Tübingen, Germany; 60 Department of Nuclear Medicine, University Tuebingen, Tübingen, Germany; 61 Institute of Clinical Epidemiology and applied Biostatistics, University Tuebingen, Tübingen, Germany; 62 Cancer Imaging Department, King’s College London and Guys & St Thomas’ Hospitals, London, UK; 63 Forth Valley Royal Hospital, Scotland, UK; 64 Department of Medical Physics, Ninewells Hospital, Dundee, UK; 65 Department of Breast Imaging, Ninewells Hospital, Dundee, UK; 66 Department of Pathology, Ninewells Hospital, Dundee, UK; 67 Department of Clinical Radiology, Ninewells Hospital, Dundee, UK; 68 Department of Surgical Oncology, MD Anderson Cancer Centre, Houston, USA; 69 Homerton Hospital, Homerton Row, London, UK; 70 University Hospital G.B. Rossi, Verona, Italy; 71 German Cancer Research Center, Heidelberg, Germany; 72 The Royal Marsden Hospital, London, UK; 73 Glasgow Royal Infirmary, Scotland, UK; 74 Ipswich Hospital, University of Suffolk, Heath Rd, Ipswich, Suffolk UK; 75 Ipswich Hospital, University of Suffolk, Heath Rd, Ipswich, Suffolk UK; 76 University of Nairobi, Nairobi, Kenya; 77 European Institute of Oncology, Via Ripamonti 435, Milano, Italy; 78 Queen Elizabeth University Hospital, 1345 Govan Rd, Glasgow, G51 4TF UK; 79 Khoo Teck Puat Hospital, Alexandra Health Group, Yishun, Singapore; 80 Hospital Aleman, Buenos Aires, Argentina; 81 Dr. B. Borooah Cancer Institute, Guwahati, India; 82 Cancer Imaging Department, King’s College London and Guys & St Thomas’ Hospitals, London, UK; 83 North Cumbria University Hospitals NHS Trust, Cumberland Infirmary, Newtown Road, Carlisle, Cumbria CA2 7HY UK; 84 West of Scotland Breast Screening Centre, Glasgow, UK; 85 West of Scotland Breast Screening Centre, Glasgow, UK; 86 North Lincolnshire & Goole NHS Foundation Trust, Scunthorpe, UK; 87 Hull and East Yorkshire Hospitals NHS Trust, Kingston upon Hull, UK; 88 Oxford University Hospitals NHS Foundation Trust, Oxford, UK; 89 University of Michigan Health System, Ann Arbor, Michigan USA; 90 Hospital Universitario Cruces, Bizkaia, Spain; 91 Barts Health NHS Trust, London, UK; 92 Deustches Hospital, Buenos Aires, Argentina; 93 Stobhill Hospital, 133 Balornock Rd, Glasgow, G21 3UW UK; 94 Deutsches Hospital, Buenos Aires, Argentina; 95 University of Michigan Medical Center, Ann Arbor, Michigan USA; 96 Royal Infirmary, Glasgow, UK; 97 National Cancer Centre, Singapore, Singapore; 98 St. Vincent’s Medical Center, Bridgeport, Connecticut USA; 99 St. Vincent’s Medical Center, Bridgeport, Connecticut USA; 100 Guy’s and St Thomas’ Hospitals NHS Foundation Trust, London, UK; 101 Oxford University Hospitals NHS Foundation Trust, London, UK; 102 Glasgow Royal Infirmary, 84 Castle St, Glasgow, G4 0ET UK; 103 North Lincolnshire & Goole NHS Foundation Trust, Scunthorpe, UK; 104 North Lincolnshire & Goole NHS Foundation Trust, Scunthorpe, UK; 105 Velindre Cancer Centre, Cardiff, UK; 106 Oxford University Hospitals NHS Foundation Trust, Oxford, UK; 107 Oxford University Hospitals NHS Foundation Trust, London, UK; 108 Hospital Alemán, Buenos Aires, Argentina; 109 Peter MacCallum Cancer Centre, Melbourne, Australia

## Abstract

O1 Tumour heterogeneity: what does it mean?

Dow-Mu Koh

O2 Skeletal sequelae in adult survivors of childhood cancer

Sue Creviston Kaste

O3 Locoregional effects of breast cancer treatment

Sarah J Vinnicombe

O4 Imaging of cancer therapy-induced CNS toxicity

Giovanni Morana, Andrea Rossi

O5 Screening for lung cancer

Christian J. Herold

O6Risk stratification of lung nodules

Theresa C. McLoud

O7 PET imaging of pulmonary nodules

Kirk A Frey

O8 Transarterial tumour therapy

Bernhard Gebauer

O9 Interventional radiology in paediatric oncology

Derek Roebuck

O10 Image guided prostate interventions

Jurgen J. Fütterer

O11 Imaging cancer predisposition syndromes

Alexander J. Towbin

O12Chest and chest wall masses

Thierry AG Huisman

O13 Abdominal masses: good or bad?

Anne MJB Smets

O14 Hepatobiliary MR contrast: enhanced liver MRI for HCC diagnosis and management

Giovanni Morana

O15 Role of US elastography and multimodality fusion for managing patients with chronic liver disease and HCC

Jeong Min Lee

O16 Opportunities and challenges in imaging metastatic disease

Hersh Chandarana

O17 Diagnosis, treatment monitoring, and follow-up of lymphoma

Marius E. Mayerhoefer, Markus Raderer, Alexander Haug

O18 Managing high-risk and advanced prostate cancer

Matthias Eiber

O19 Immunotherapy: imaging challenges

Bernhard Gebauer

O20 RECIST and RECIST 1.1

Andrea Rockall

O21 Challenges of RECIST in oncology imaging basics for the trainee and novice

Aslam Sohaib

O22 Lymphoma: PET for interim and end of treatment response assessment: a users’ guide to the Deauville Score

Victoria S Warbey

O23 Available resources

Hebert Alberto Vargas

O24 ICIS e-portal and the online learning community

Dow-Mu Koh

O25 Benign lesions that mimic pancreatic cancer

Jay P Heiken

O26 Staging and reporting pancreatic malignancies

Isaac R Francis, Mahmoud, M Al-Hawary, Ravi K Kaza

O27 Intraductal papillary mucinous neoplasm

Giovanni Morana

O28 Cystic pancreatic tumours

Mirko D’Onofrio

O29 Diffusion-weighted imaging of head and neck tumours

Harriet C. Thoeny

O30 Radiation injury in the head and neck

Ann D King

O31 PET/MR of paediatric brain tumours

Giovanni Morana, Arnoldo Piccardo, Maria Luisa Garrè, Andrea Rossi

O32 Structured reporting and beyond

Hebert Alberto Vargas

O33 Massachusetts General Hospital experience with structured reporting

Theresa C. McLoud

O34 The oncologist’s perspective: what the oncologist needs to know

Nick Reed

O35 Towards the cure of all children with cancer: global initiatives in pediatric oncology

Carlos Rodriguez-Galindo

O36 Multiparametric imaging of renal cancers

Hersh Chandarana

O37 Linking imaging features of renal disease and their impact on management strategies

Hebert Alberto Vargas

O38 Adrenals, retroperitoneum and peritoneum

Isaac R Francis, Ashish P Wasnik

O39 Lung and pleura

Stefan Diederich

O40 Advances in MRI

Jurgen J. Fütterer

O41 Advances in molecular imaging

Wim J.G. Oyen

O42 Incorporating advanced imaging, impact on treatment selection and patient outcome

Cheng Lee Chaw, Nicholas van As

S1 Combining ADC-histogram features improves performance of MR diffusion-weighted imaging for Lymph node characterisation in cervical cancer

Igor Vieira, Frederik De Keyzer, Elleke Dresen, Sileny Han, Ignace Vergote, Philippe Moerman, Frederic Amant, Michel Koole, Vincent Vandecaveye

S2 Whole-body diffusion-weighted MRI for surgical planning in patients with colorectal cancer and peritoneal metastases

R Dresen, S De Vuysere, F De Keyzer, E Van Cutsem, A D’Hoore, A Wolthuis, V Vandecaveye

S3 Role of apparent diffusion coefficient (ADC) diffusion-weighted MRI for predicting extra capsular extension of prostate cancer.

P. Pricolo (paola.pricolo@ieo.it), S. Alessi, P. Summers, E. Tagliabue, G. Petralia

S4 Generating evidence for clinical benefit of PET/CT – are management studies sufficient as surrogate for patient outcome?

C. Pfannenberg, B. Gückel, SC Schüle, AC Müller, S. Kaufmann, N. Schwenzer, M. Reimold,C. la Fougere, K. Nikolaou, P. Martus

S5 Heterogeneity of treatment response in skeletal metastases from breast cancer with 18F-fluoride and 18F-FDG PET

GJ Cook, GK Azad, BP Taylor, M Siddique, J John, J Mansi, M Harries, V Goh

S6 Accuracy of suspicious breast imaging—can we tell the patient?

S Seth, R Burgul, A Seth

S7 Measurement method of tumour volume changes during neoadjuvant chemotherapy affects ability to predict pathological response

S Waugh, N Muhammad Gowdh, C Purdie, A Evans, E Crowe, A Thompson, S Vinnicombe

S8 Diagnostic yield of CT IVU in haematuria screening

F. Arfeen, T. Campion, E. Goldstraw

S9 Percutaneous radiofrequency ablation of unresectable locally advanced pancreatic cancer: preliminary results

D’Onofrio M, Ciaravino V, Crosara S, De Robertis R, Pozzi Mucelli R

S10 Iodine maps from dual energy CT improve detection of metastases in staging examinations of melanoma patients

M. Uhrig, D. Simons, H. Schlemmer

S11Can contrast enhanced CT predict pelvic nodal status in malignant melanoma of the lower limb?

Kate Downey

S12 Current practice in the investigation for suspected Paraneoplastic Neurological Syndromes (PNS) and positive malignancy yield.

S Murdoch, AS Al-adhami, S Viswanathan

P1 Technical success and efficacy of Pulmonary Radiofrequency ablation: an analysis of 207 ablations

S Smith, P Jennings, D Bowers, R Soomal

P2 Lesion control and patient outcome: prospective analysis of radiofrequency abaltion in pulmonary colorectal cancer metastatic disease

S Smith, P Jennings, D Bowers, R Soomal

P3 Hepatocellular carcinoma in a post-TB patient: case of tropical infections and oncologic imaging challenges

TM Mutala, AO Odhiambo, N Harish

P4 Role of apparent diffusion coefficient (ADC) diffusion-weighted MRI for predicting extracapsular extension of prostate cancer

P. Pricolo, S. Alessi, P. Summers, E. Tagliabue, G. Petralia

P5 What a difference a decade makes; comparison of lung biopsies in Glasgow 2005 and 2015

M. Hall, M. Sproule, S. Sheridan

P6 Solid pseudopapillary tumour of pancreas: imaging features of a rare neoplasm

KY Thein, CH Tan, YL Thian, CM Ho

P7 MDCT - pathological correlation in colon adenocarcinoma staging: preliminary experience

S De Luca, C Carrera, V Blanchet, L Alarcón, E Eyheremnedy

P8 Image guided biopsy of thoracic masses and reduction of pneumothorax risk: 25 years experience

B K Choudhury, K Bujarbarua, G Barman

P9 Tumour heterogeneity analysis of 18F-FDG-PET for characterisation of malignant peripheral nerve sheath tumours in neurofibromatosis-1

GJ Cook, E Lovat, M Siddique, V Goh, R Ferner, VS Warbey

P10 Impact of introduction of vacuum assisted excision (VAE) on screen detected high risk breast lesions

L Potti, B Kaye, A Beattie, K Dutton

P11 Can we reduce prevalent recall rate in breast screening?

AA Seth, F Constantinidis, H Dobson

P12 How to reduce prevalent recall rate? Identifying mammographic lesions with low Positive Predictive Value (PPV)

AA Seth (archana.seth@nhs.net), F Constantinidis, H Dobson

P13 Behaviour of untreated pulmonary thrombus in oncology patients diagnosed with incidental pulmonary embolism on CT

R. Bradley, G. Bozas, G. Avery, A. Stephens, A. Maraveyas

P14 A one-stop lymphoma biopsy service – is it possible?

S Bhuva, CA Johnson, M Subesinghe, N Taylor

P15 Changes in the new TNM classification for lung cancer (8^th^ edition, effective January 2017)

LE Quint, RM Reddy, GP Kalemkerian

P16 Cancer immunotherapy: a review of adequate imaging assessment

G González Zapico, E Gainza Jauregui, R Álvarez Francisco, S Ibáñez Alonso, I Tavera Bahillo, L Múgica Álvarez

P17 Succinate dehydrogenase mutations and their associated tumours

O Francies, R Wheeler, L Childs, A Adams, A Sahdev

P18 Initial experience in the usefulness of dual energy technique in the abdomen

SE De Luca, ME Casalini Vañek, MD Pascuzzi, T Gillanders, PM Ramos, EP Eyheremendy

P19 Recognising the serious complication of Richter’s transformation in CLL patients

C Stove, M Digby

P20 Body diffusion-weighted MRI in oncologic practice: truths, tricks and tips

M. Nazar, M. Wirtz, MD. Pascuzzi, F. Troncoso, F. Saguier, EP. Eyheremendy

P21 Methotrexate-induced leukoencephalopathy in paediatric ALL Patients

D.J. Quint, L. Dang, M. Carlson, S. Leber, F. Silverstein

P22 Pitfalls in oncology CT reporting. A pictorial review

R Rueben, S Viswanathan

P23 Imaging of perineural extension in head and neck tumours

B Nazir, TH Teo, JB Khoo

P24 MRI findings of molecular subtypes of breast cancer: a pictorial primer

K Sharma, N Gupta, B Mathew, T Jeyakumar, K Harkins

P25 When cancer can’t wait! A pictorial review of oncological emergencies

K Sharma, B Mathew, N Gupta, T Jeyakumar, S Joshua

P26 MRI of pancreatic neuroendocrine tumours: an approach to interpretation

D Christodoulou, S Gourtsoyianni, A Jacques, N Griffin, V Goh

P27 Gynaecological cancers in pregnancy: a review of imaging

CA Johnson, J Lee

P28 Suspected paraneoplastic neurological syndromes - review of published recommendations to date, with proposed guideline/flowchart

JA Goodfellow, AS Al-adhami, S Viswanathan

P29 Multi-parametric MRI of the pelvis for suspected local recurrence of prostate cancer after radical prostatectomy

R Bradley

P30 Utilisation of PI-RADS version 2 in multi-parametric MRI of the prostate; 12-months experience

R Bradley

P31 Radiological assessment of the post-chemotherapy liver

A Yong, S Jenkins, G Joseph

P32 Skeletal staging with MRI in breast cancer – what the radiologist needs to know

S Bhuva, K Partington

P33 Perineural spread of lympoma: an educational review of an unusual distribution of disease

CA Johnson, S Bhuva, M Subesinghe, N Taylor

P34 Visually isoattenuating pancreatic adenocarcinoma. Diagnostic imaging tools.

C Carrera, A Zanfardini, S De Luca, L Alarcón, V Blanchet, EP Eyheremendy

P35 Imaging of larynx cancer: when is CT, MRI or FDG PET/CT the best test?

K Cavanagh, E Lau

## Oral presentations

### O1 Tumour heterogeneity: what does it mean?

#### Dow-Mu Koh

##### Royal Marsden Hospital, Sutton, Surrey, SM2 5PT, UK

Cancers arise from unregulated cellular growth and exhibits a range of pathophysiological characteristics and behaviour. The term “tumour heterogeneity” encompasses a broad range of tumour features that have arisen as a result of different mechanistic processes including stem-cell origin, tumour evolution (linked to genetic mutations and adverse tumour microenvironment) and clonal resistance (as a consequence of treatment). Tumour heterogeneity may be intra-tumoural (i.e. within the same tumour), inter-tumoural (i.e. between lesions in the same patient) and inter-individual (i.e. between patients).

The understanding of tumour heterogeneity is important because it is a barrier to curing cancers; and improved understanding can lead to better individualised treatments. Tumour heterogeneity is linked to treatment resistance and the emergence of aggressive tumour phenotypes. There is thus a need for biomarkers to identify relevant tumour sub-clones to which specific therapies may be directed.

Current approaches to enhance our understanding of tumour heterogeneity include genomics analysis using either multi-tumoural region and/or longitudinal tumor tissue sampling. However, non-invasive imaging using CT, MRI and PET imaging can provide an overview of the imaging characteristics of multiple disease sites across the body, thereby provide critical information about intra- and inter-tumour heterogeneity.

Using functional and molecular imaging techniques, imaging can be used to quantitatively measure different aspects of tumour biology; thus identifying imaging phenotypes linked to different stem-cell origins, gene expression, tumour evolution, treatment efficacy (including differential treatment response) and the emergence of clonal resistance. The emerging evidence for the use of imaging to explore and understand tumour heterogeneity will be presented and discussed.

### O2 Skeletal sequelae in adult survivors of childhood cancer

#### Sue Creviston Kaste^1,2,3^ (sue.kaste@stjude.org)

##### ^1^Department of Diagnostic Imaging, St. Jude Children’s Research Hospital, 262 Danny Thomas Place, MSN #220, Memphis, TN 38105, USA; ^2^Department of Oncology, St. Jude Children’s Research Hospital, 262 Danny Thomas Place, Memphis, TN 38105, USA; ^3^Department of Radiology, University of Tennessee Health Science Center, 920 Madison Ave, Memphis, TN 38103, USA

Improved diagnostic methods, treatment and understanding of the biology of childhood tumors over the past several decades have led to improved patient survival. In 2009, the U.S. population of childhood cancer survivors was estimated at one in 570 young adults aged 20 to 34 years [1]. This rapidly growing population underscores the importance of studying long-term complications of cancer therapy. While childhood cancer patients are returning to the mainstream of life, toxicities from prior therapy may compound or potentiate changes typically seen with the normal aging process. Physical consequences of disease and therapy (e.g., surgery, chemotherapy and radiation therapy) such as scoliosis, craniofacial dysplasia, and limb-length discrepancy, may present functional limitations, psychosocial challenges, and require extensive surgical interventions.

However, skeletal toxicities [2] such as osteonecrosis [3-5] and deficits in bone mineral density [6, 7] are typically silent until they reach advanced stages when attempts at amelioration may be unsuccessful. These two sequelae result from multifactorial interactions including genetic predisposition, disease, therapy (chemotherapy and radiation therapy) and physical activity. As both bone mineral density deficits and osteonecrosis may occur in a single patient, therapeutic interventions may be complex and standard treatment practices for one of these sequel may exacerbate the other toxicity.

Osteonecrosis is an important long-term toxicity that can compromise joint functionality and, thus, quality of life in survivors of childhood cancer in whom it develops. Its development is multi-factorial but understanding of this process in pediatric oncology is complex and incomplete. Osteonecrosis affects up to one-third of pediatric leukemia patients and is typically asymptomatic in its early stage when most susceptible to treatment [3]. Despite this fact, it is only when pain occurs that osteonecrosis is typically suspected. MR imaging is the diagnostic modality of choice for screening, follow-up and risk prediction of functional deterioration in early stages and readily demonstrates osteonecrotic parameters such as size of the lesion, involvement of articular surface, presence of a crescent sign, etc. Clinical symptoms and demographics are predictive of disease progression in pediatric steroid-induced hip osteonecrosis [3].

This presentation will concentrate on what is known, factors as yet unknown, and potential implications of such toxicity. It will focus on bone mineral density deficits which may predispose childhood cancer survivors to earlier onset and more severe osteopenia and osteoporosis than the normal population. Also detailed, will be osteonecrosis which predisposes survivors to impairment of joint function. Therapeutic interventions for osteonecrosis are currently limited and ultimately, may lead to joint resurfacing or replacement at a young age.

**Acknowledgements**

Supported in part by grant P30 CA-21765 from the National Institutes of Health, a Center of Excellence grant from the State of Tennessee, the Le Bonheur Foundation (Memphis TN), and the American Lebanese Syrian Associated Charities (ALSAC).

**References**

1. Mariotto AB, Rowland JH, Yabroff KR, Scoppa S, Hachey M, Ries L, Feuer EJ. Long-term survivors of childhood cancers in the United States. Cancer Epidemiol Biomarkers Prev. 2009 Apr;18(4):1033-40. doi:10.1158/1055-9965.EPI-08-0988. Epub 2009 Mar 31.

2. Kaste SC. Skeletal toxicities of treatment in children with cancer. Pediatr Blood Cancer. 2008 Feb;50(2 Suppl):469-73; discussion 486.

3. Kaste SC, Pei D, Cheng C, Neel MD, Bowman WP, Ribeiro RC, Metzger ML, Bhojwani D, Inaba H, Campbell P, Rubnitz JE, Jeha S, Sandlund JT, Downing JR, Relling MV, Pui CH, Howard SC. Utility of early screening magnetic resonance imaging for extensive hip osteonecrosis in pediatric patients treated with glucocorticoids. J Clin Oncol. 2015 Feb 20;33(6):610-5. doi:10.1200/JCO.2014.57.5480. Epub 2015 Jan 20.

4. Kawedia JD, Kaste SC, Pei D, Panetta JC, Cai X, Cheng C, Neale G, Howard SC, Evans WE, Pui CH, Relling MV. Pharmacokinetic, pharmacodynamic, and pharmacogenetic determinants of osteonecrosis in children with acute lymphoblastic leukemia. Blood. 2011 Feb 24;117(8):2340-7; quiz 2556. doi:10.1182/blood-2010-10-311969. Epub 2010 Dec 10.

5. Karimova EJ, Rai SN, Howard SC, Neel M, Britton L, Pui CH, Kaste SC. Femoral head osteonecrosis in pediatric and young adult patients with leukemia or lymphoma. J Clin Oncol. 2007 Apr 20;25(12):1525-31.

6. Wasilewski-Masker K, Kaste SC, Hudson MM, Esiashvili N, Mattano LA, Meacham LR. Bone mineral density deficits in survivors of childhood cancer: long-term follow-up guidelines and review of the literature. Pediatrics. 2008 Mar;121(3):e705-13. doi:10.1542/peds.2007-1396.

7. Kaste SC, Jones-Wallace D, Rose SR, Boyett JM, Lustig RH, Rivera GK, Pui CH, Hudson MM. Bone mineral decrements in survivors of childhood acute lymphoblastic leukemia: frequency of occurrence and risk factors for their development. Leukemia. 2001 May;15(5):728-34.

### O3 Locoregional effects of breast cancer treatment

#### Sarah J Vinnicombe

##### Division of Cancer Research, University of Dundee, Ninewells Hospital and Medical School, Dundee, Angus, DD1 9SY, UK

The treatment of both early and late stage breast cancer has evolved markedly over the past three decades, hence the significant reduction in breast cancer mortality observed in developed countries over the past three decades [1]. However, the majority of breast cancers are still treated with a combination of surgery and radiotherapy, generally with some form of axillary procedure. Thus, the commonest locoregional effects of breast cancer treatment are secondary to surgery and to a lesser extent, radiotherapy.

Surgical effects can be acute or chronic. Acute effects include post-operative seromas, haematomas, and infection. Postoperative seromas are extremely common and can persist for years [2]. Occasionally they may be very uncomfortable, especially in the axilla, necessitating ultrasound-guided aspiration, often repeated. Rare consequences of autologous reconstructions secondary to inadequate tissue perfusion include wound breakdown and necrosis, but in the chronic phase, the commonest complication of surgery by far is fat necrosis, which can present with a symptomatic lump. Though the imaging appearances of mature fat necrosis are characteristic, it can cause diagnostic confusion initially, with solid-appearing masses on mammography, ultrasound and MRI, and indeterminate or suspicious mammographic microcalcification [3]. Together with the less common epidermal inclusion cysts, the major issue with these benign complications is the resultant patient anxiety, since the main differential diagnosis is obviously local recurrence [4]. However, post-surgical complications tend to occur earlier than recurrent disease and the imaging differentiation is usually straightforward.

The short-term effects of whole breast radiotherapy are predictable, with radiation-induced oedema, skin and trabecular thickening, which is readily apparent at mammography and ultrasound. Such changes peak around 18 months post radiotherapy and then generally diminish. However, patients may be symptomatic with tenderness and focal thickening for years after radiotherapy. The longer-term effects are less predictable, particularly the development of radiation induced angiosarcoma [5]. The latency for this condition ranges from 3 to 20 years but with breast cancer increasingly becoming a chronic disease, it is likely that it will be seen more often. The other devastating effect of radiation to the axilla and supraclavicular fossa is radiation induced brachial plexopathy [6]. This highly debilitating iatrogenic complication is mercifully much less common with modern conformal radiotherapy techniques and careful observance of fields and dosages. Nonetheless the differentiation between radiation induced and malignant plexopathy can be challenging and may necessitate MRI and PET scanning [7, 8].

Recognition of the long-term sequelae of conventional treatment of breast cancer has resulted in a move towards more breast conservation, (with oncoplastic surgical techniques if necessary), less axillary intervention (with less resultant lymphoedema) and accelerated partial breast irradiation techniques. These all result in specific imaging findings on surveillance mammography which the breast radiologist needs to be aware of, for example more frequent fat necrosis at the site of the surgical excision after intra-operative radiotherapy [9]. Finally, it is important for the general cross-sectional radiologist to be aware of the spectrum of normal imaging findings at CT after complex reconstructive surgery and to recognise when a finding needs further evaluation [10].

**References**

1. Cancer Research UK, http://www.cancerresearchuk.org/health-professional/cancer-statistics/statistics-by-cancer-type/breast-cancer/mortality, accessed 07/2016.

2. Kim SM, Park JM. Normal and abnormal ultrasound findings at the mastectomy site. Radiographics 2004;**24**:357-65

3. Hogge JP, Robinson RE, Magnant CM et al. Fat necrosis of the breast: clinical, mammographic and sonographic features. Radiographics 1995;**15**:1347-56

4. Fersis N, Hoenig A, Relakis K et al. Skin-sparing mastectomy and immediate breast reconstruction: incidence of recurrence in patients with invasive breast cancer. Breast 2004;**13**:488-93

5. Kirova YM, Vilcoq JR, Asselain B et al. Radiation-induced sarcomas after radiotherapy for breast carcinoma: a large scale institution review. Cancer 2005;**104**:856-63

6. Senkus-Konefka E, Jassem J. Complications of breast-cancer radiotherapy. Clin Oncol 2006;**18**:229-35

7. Lutz AM, Gold G, Beaulieu C. MR imaging of the brachial plexus. Neuroimaging Clin N Am 2014;**24**:91-108

8. Chandra P, Purandare N, Agrawal A et al. Indian J Nucl Med 2016;**31**:123-7

9. Elsberger B, Romsauerova A, Vinnicombe S et al. Comparison of mammographic findings after intraoperative radiotherapy or external beam whole breast radiotherapy. Eur J Surg Oncol 2014;**40**:163-7

10. Jung JI, Kim HK, Park SH et al. Thoracic manifestations of breast cancer and its therapy. Radiographics 2004;**24**:1269-85

### O4 Imaging of cancer therapy-induced CNS toxicity

#### Giovanni Morana, Andrea Rossi

##### Neuroradiology Unit, Istituto Giannina Gaslini, Genoa, GE, 16145, Italy

###### **Correspondence:** Giovanni Morana (giovannimorana@gaslini.org) – Neuroradiology Unit, Istituto Giannina Gaslini, Genoa, GE, 16145, Italy

Cancer therapy-induced CNS toxicity comprises a wide spectrum of clinical and radiological complications, causing significant morbidity and mortality [1].

Adequate interpretation of the neuroimaging findings of patients experiencing neurotoxicity represents a complex diagnostic challenge and requires a detailed knowledge of the underlying pathology (systemic disease or CNS tumour), of the treatment protocol and schedule used, and of the timing of symptoms’ onset.

Early recognition of toxic injury is fundamental to discontinue the offending agent, to judge the overall success and efficacy of therapy and to institute early treatment of neurotoxic complications [2].

Chemotherapy (CT), Radiotherapy (RT), or both in combination, can determine acute and delayed brain side effects [3].

CT induced toxicity can be caused by several drugs and among different patterns of injury the most common presentations include Posterior Reversible Encephalopathy Syndrome and Acute Toxic Leukoencephalopathy [4].

Among RT induced complications “pseudoprogression” and radiation necrosis are respectively early and late delayed effects, enhanced by concomitant CT, that can be associated with clinical worsening mimicking disease progression [5,6].

Late delayed RT induced complications include also diffuse radiation leukoencephalopathy, cavernous malformations, mineralizing microangiopathy and pituitary disfunction, which are extremely common in the paediatric population [7].

Conventional Magnetic Resonance Imaging (MRI) with current high field systems is the gold standard method for the evaluation of therapy related brain alterations. Advanced imaging modalities such as, Diffusion Weighted Imaging, Magnetic Resonance Spectroscopy and Perfusion Weighted Imaging, as well as Positron Emission Tomography with amino-acid tracers, may substantially improve the ability to orient a proper diagnosis and to guide patient management. When MRI is unavailable or contraindicated, Computerized Tomography (CT) remains an alternative diagnostic tool, even though its role in acute settings is mainly limited to rule out haemorrhage.

Evaluation and depiction of the most common neuroimaging patterns of brain injury induced by cancer therapy is the focus of the present work.

**References**

1. Dietrich J, Klein JP. Imaging of cancer therapy-induced central nervous system toxicity. Neurol Clin. 2014,32:147-57.

2. Rossi A, Morana G, Gandolfo C, Severino M. Neuroradiology of chemotherapeutic. Neurotoxicity in children. Neuroradiol J. 2010,23:183-90

3. Vázquez E, Delgado I, Sánchez-Montañez A, Barber I, Sánchez-Toledo J, Enríquez G. Side effects of oncologic therapies in the pediatric central nervous system: update on neuroimaging findings. Radiographics. 2011,31:1123-39.

4. Iyer RS, Chaturvedi A, Pruthi S, Khanna PC, Ishak GE. Medication neurotoxicity in children. Pediatr Radiol. 2011,41:1455-64.

5. Pruzincová L, Steno J, Srbecký M, Kalina P, Rychlý B, Boljesíková E, et al. MR imaging of late radiation therapy- and chemotherapy-induced injury: a pictorial essay. Eur Radiol. 2009,19:2716-27.

6. Fatterpekar GM, Galheigo D, Narayana A, Johnson G, Knopp E. Treatment-related change versus tumor recurrence in high-grade gliomas: a diagnostic conundrum-use of dynamic susceptibility contrast-enhanced (DSC) perfusion MRI. AJR Am J Roentgenol. 2012,198:19-26.

7. Faraci M, Morana G, Bagnasco F, Barra S, Polo P, Hanau G, et al. Magnetic resonance imaging in childhood leukemia survivors treated with cranial radiotherapy: a cross sectional, single center study. Pediatr Blood Cancer. 2011,57:240-6.

### O5 Screening for lung cancer

#### Christian J. Herold

##### Department of Biomedical Imaging and Image Guided Therapy, Medical University of Vienna/Vienna General Hospital, 1090 Vienna, Austria

The most compelling evidence supporting the use of low dose computed tomography in the screening of high risk populations for lung cancer was generated by the National Lung Cancer Screening Trial carried out in the United States by the National Cancer Institute and the American College of Radiology Imaging Network. This trial was a randomised prospective study which included over 53,000 participants. Data from the NLST demonstrated that screening reduced mortality by 20 % in the CT arm [1]. Other smaller studies carried out in Europe have reported no mortality benefit. However, these studies included a younger screening population, had a smaller number of participants and probably did not have the power to show a mortality benefit. Major medical societies and US government agencies such as US Preventative Services Task Force and the Center for Medicare Services have now recommended LDCT. These decisions not only recommend screening but require US insurance companies and Medicare in the US to provide reimbursement.

In the United States, the Center for Medicare and Medicaid Services (CMS) decision to provide reimbursement for lung cancer screening with low dose CT included a number of specific criteria for the establishment of lung cancer screening programs [2]. It requires a written order from a physician for the initial and subsequent screens and a required office visit with the referring physician in which the patient would be counseled concerning lung cancer screening and a shared decision making would be implemented. Also included are criteria for radiologist eligibility including certification by the American Board of Radiology or equivalent and the interpretation of at least 300 chest CTs in the past 3 years and documentation of continuing medical education. Radiology imaging centers must be accredited as well. The American College of Radiology is providing such accreditation. CMS is also requiring collection and submission of data by an approved national registry for each screen performed.

The American College of Radiology has developed a reporting system for lung cancer screening designated “Lung Rads” [3]. It is very similar to Bi-Rads that includes numeric categories. It associates CT findings with guideline based management decisions. LDCT findings are categorized from 1-4 according to the likelihood of malignancy. 1 and 2 are considered benign findings and 3 and 4 have a higher probability of malignancy. There are descriptions for each of the 4 categories plus an enumeration of findings. The categories include characterization of nodules including size and density (solid, part-solid, or non- solid ground glass). In addition to the categories and findings there are guidelines for management in each category with estimates of the probability of malignancy and the estimated population prevalence.

Some of the caveats of screening result from the limits of generalization of the NLST results based on risk groups and demographics. There are also potential harms and complications from the LDCT screening which include radiation exposure, overdiagnosis/overmanagement, the use of invasive procedures for diagnosis and the high false positive rate [4]. Nevertheless, these potential limitations, harms and complications seem to be outweighted by the benefits of screening.

In the future, we will see a consolidation of the body of evidence and gain new insights through the pooling of the European trial data (Nelson [5]), and the UK Lung Cancer Screening trial pilot study [6].

**References:**

1) Aberle DR, Adams AM, Berg CD, Black WC, Clapp JD, Fagerstrom RM, et al, National Lung Screening Trial Research Team. Reduced lung-cancer mortality with low-dose computed tomographic screening. N Engl J Med. 2011;365:395-409

2) Pinksy PF, Gierada DS, Hocking W, Patz Jr EF, Kramer BS. National Lung Screening Trial Findings by Age: Medicare-Eligible Versus Under-65 Population. Ann Intern Med. 2014. 161(9):627-633

3) Lung CT screening reporting and data system (Lung-RADS). Available at: http://www.acr.org/Quality-Safety/Resources/LungRADS. Accessed 25 June 2014.

4) Parker MS, Groves RC, Fowler AA, Shepherd RW, Cassano AD, Cafaro PC, Chestnut GT. Lung Cancer Screening With Low-dose Computed Tomography. J Thorac Imaging 2014. 00;00

5) Horeweg N, van Rosmalen J, Heuvelmans MA,van der Aalst CM, Vliegenthart R, Scholten ET, ten Haaf K, Nackaerts K, Lammers JW, Weenink C, Groen HJ, van Ooijen P, de Jong PA, de Bock GH, Mali W, de Koning HJ, Oudkerk M. Lung cancer probability in patients with CT-detected pulmonary nodules: a prespecified analysis of data from the NELSON trial of low-dose CT screening. Lancet Oncol 2014. 15(12):1332-41

6) Field JK, Duffy SW, Baldwin DR, Brain KE, Devaraj A, Eisen T, Green BA, Holemans JA, Kavanagh T, Kerr KM, Ledson M, Lifford K, McRonald FE, Nair A, Page RD, Parmar MKB, Rintoul RC, Screaton N, Wald NJ, Weller D, Whynes DK, Williamson PR, Yadegarfar G, Hansell DM. The UK Lung Cancer Screening Trial: a pilot randomized controlled trial of low-dose computed tomography screening for the early detection of lung cancer. Health Technology Assessment May 2016. 20;40:1366-5278

### O6 Risk stratification of lung nodules

#### Theresa C. McLoud

##### Department of Radiology, Massachusetts General Hospital, Harvard Medical School, 55 Fruit Street, Boston, MA 02114, USA

Risk stratification is important for large and small nodules. Special issues arise with small pulmonary nodules defined as less than 8 mm. The vast majority of these are benign.

There are both clinical and imaging risk factors for malignancy that should be taken into account. Clinical risk factors include older age, history of smoking, history of extrathoracic cancer within 5 years, and family history of cancer. The CT characteristics that indicate risk from malignancy include a size of greater than 8 mm, certain border characteristics, density features, growth, and location. A presumptive benign diagnosis can be made on the basis of 2 year stability excluding ground glass nodules which require longer follow-up. Benign calcification (diffuse, central, laminated, and popcorn), the presence of fat, a size less than 4 mm and perifissural location are very strong predictors of benignity and no further evaluation of such nodules is required.

Adequate evaluation of solitary pulmonary nodules requires thin section CT with a 1.5 to 2 mm slice thickness.

The solidity of nodules is important in determining their malignant potential. Among purely solid nodules, a size greater than 8 mm, location in the upper lobes, spiculated or lobulated contour, and internal characteristics such as eccentric calcification, air bronchograms and cavitation are helpful predictors of malignancy in addition to the growth rate. Small nodules less than 4 mm have less than a 0.5 % chance of malignancy even in smokers. Nodules in the range of 8-10 mm or greater have a 10-20 % chance of malignancy. The management of small solid nodules less than 8 mm often consists of imaging follow-up according to current recommendations by the Fleischner Society.

Subsolid nodules represent the spectrum of peripheral adenocarcinomas of the lung and may be mixed part solid/part ground glass or pure ground glass nodules. Part solid nodules have the highest likelihood of malignancy in the range of 60 to 70 %. Malignancy in subsolid nodules is associated both with lesion growth and the development and growth of the solid component.

In summary, small solitary pulmonary nodules commonly detected on CT are mostly benign. It is imperative that clinical risk factors which are mostly socio-demographic be evaluated. Important imaging findings in determining the risk of malignancy includes size, location, morphology and classic benign features, growth rate, and solidity.

References:

1. Ost DE, Gould MK: **Decision making in patients with pulmonary nodules.** *Amer J of Resp and Critical Care Medcine* 2012, 185(4):363-372.

2. MacMahon H, Austin J, Gamsu G, et al: **Guidelines for management of small pulmonary nodules detected on CT scans: A statement from the Fleischner Society.** Editorial. *Radiology* 2005, 237(2):1-7.

3. Naidich DP, Bankier AA, MacMahon H, et al: **Recommendations for the management of subsolid pulmonary nodules detected at CT: A statement from the Fleischner Society.** *Radiology* 2013, 266(1):1-11.

### O7 PET imaging of pulmonary nodules

#### Kirk A Frey

##### Division of Nuclear Medicine and Molecular Imaging, Department of Radiology, The University of Michigan, Ann Arbor, MI 48109-5028, USA

Over the past 2 decades, positron emission tomography with the tracer [^18^F]fluorodeoxyglucose (FDG-PET) has gained increasing utility and importance in the diagnosis and management of patients with pulmonary nodules that may represent non-small cell lung cancer (NSCLC) [1]. It is evident that FDG-PET does not replace the need for pathologic examination, but contributes to decisions about when to obtain tissue and from what location(s). Studies have shown repeatedly that overall patient survival and progression-free survival in NSCLC are predicted by the lesion intensity in FDG-PET at initial characterization [2-4]. Thus, although many non-neoplastic lesions can be FDG-avid, and while many instances of low-grade malignancy may be FDG “negative”, use of serial anatomic imaging in the latter patients may suffice to characterize lesions, without significant risk of upstaging when malignancy is found in a growing lesion. Presence of avid FDG uptake in a new pulmonary lesion identifies cases where tissue diagnosis should be expedited, and may serve simultaneously to stage the malignancy. More recent evidence suggests an added role of FDG-PET in directing needle biopsy of pulmonary lesions, improving diagnostic yield [5].

An emerging application of FDG-PET in NSCLC is in the assessment of therapeutic response [6], and in the possibility of applying individualised, adaptive therapy. Studies in the use of external beam radiotherapy have identified predictive value of initial FDG activity patterns in NSCLC lesion(s) with the likelihood of post-therapeutic relapse [7]. Other studies have identified that early tumor response to radiotherapy may be predicted well before completion of dose delivery [8], supporting the novel development of protocols based on re-planning and re-targeting of remaining radiation dose midway during treatment. A similar strategy can be envisioned surrounding the use of targeted chemotherapies in advanced stage NSCLC, where FDG-PET monitoring of response at all sites of involvement could distinguish effective versus futile treatments.

**References**

1. Fletcher JW, Djulbegovie B, Soares HP, et al: **Recommendations on the use of** ^**18**^ **F-FDG PET in oncology.** *J Nucl Med* 2008, **49:**480-508.

2. Marom EM, Sarvis S, Herndon JE, Patz EF: **T1 lung cancers: Sensitivity of diagnosis with fluorodeoxyglucose PET.** *Radiology* 2002, **223:**453-459.

3. Higashi K, Udea Y, Arisaka Y, Sakuma T, et al: **Disease-free survival in NSCLC.** *J Nucl Med* 2002, **43:**39-45.

4. Nair VS, Barnett PG, Anrth L, et al; for the Veterans Affairs Solitary Nodule Accuracy Project Cooperative Studies Group: **PET scan** ^**18**^ **F-fluorodeoxyglucose uptake in patients with resected clinical stage IA non-small cell lung cancer.** *Chest* 2010, **137:**1150-1156.

5. Guralnik L, Rozenberg R, Frenkel A, Israel O, Keidar Z: **Metabolic PET/CT-guided lung lesion biopsies: Impact on diagnostic accuracy and rate of sampling error.** *J Nucl Med* 2015, **56:**518-522.

6. Hicks RJ: **Role of** ^**18**^ **F-FDG PET in assessment of response in non-small cell lung cancer.** *J Nucl Med* 2009, **50:**31S-42S.

7. Calais J, Thureau S, Dubray B, et al: **Areas of high** ^**18**^ **F-FDG uptake on preradiotherapy PET/CT identify preferential sites of local elapse after chemoradiotherapy for non-small cell lung cancer.** *J Nucl Med* 2015, **56:**196-203.

8. Kong F-MS, Frey KA, Quint LE et al: **A pilot study of [** ^**18**^ **F]fluorodeoxyglucose positron emission tomography scans during and after radiation-based therapy in patients with non-small-cell lung cancer.** *J Clin Oncol* 2007, **25:**3116-3123.

### O8 Transarterial tumour therapy

#### Bernhard Gebauer (bernhard.gebauer@charite.de)

##### Department of Radiology, Charité, Campus Virchow-Klinikum, Augustenburger Platz 1, 13353 Berlin, Germany

Transarterial tumour therapy means that the arterial route of an organ is used for selective tumour therapy. Therapeutics usually used for transarterial tumour therapy are usually small particles, chemotherapeutics and radionuclides. Transarterial tumour therapy is most often performed in primary and secondary liver tumours, and less often in other organs, like kidney, lung, prostate, uterus, etc.

For primary or secondary hepatic tumours either transarterial (bland) embolisation (TAE), transarterial chemoembolisation (TACE), hepatic arterial infusion (HAI) therapy or transarterial radioembolisation (RE) are the typical transarterial tumour therapies. A recent method that is still experimental is chemosaturation [1].

Transarterial Chemoembolization (TACE) is the standard treatment for intermediate stage (BCLC B) hepatocellular cancers (HCC). TACE utilises the fact that HCCs are perfused to 80 % by the liver artery and only to 20 % by the portal venous system. TACE uses the arterial perfusion of HCCs to introduce ischemia and chemotherapeutics into the tumour to kill tumour cells and to minimise systemic effects for the patient. TACE was developed in the early 1980s in Japan and traditionally was a mixture of lipiodol (ethyl ester of iodized fatty acids of poppy seed oil) and a chemotherapeutic agent (water-in-oil emulsion) [2]. This so called conventional or lipiodol TACE (cTACE) is usually completed by intraarterial application of gelatin sponge and this technique is established as the standard treatment for HCCs without portal vein invasion as a result of 2 randomised studies using doxorubicin [3] or cisplatium [4] as a chemotherapeutic agent. Both studies demonstrated a significant superiority of cTACE compared to best supportive care.

In the last 15 years 2 new TACE techniques were developed, TACE with drug-eluting microspheres (DEB-TACE) and TACE using degradable starch microspheres (DSM-TACE). In DEB-TACE the chemotherapeutic agent is released slowly from the embolising microparticles reducing the systemic side effects of TACE [5]. This technique showed a reduction of post-procedural abdominal pain, but failed to demonstrate a survival benefit compared to cTACE [6].

TACE is also used to stabilize HCC tumors in patients on the waiting list for liver transplantation, so called “bridge to transplantation”, and as an adjunctive to thermal local ablative techniques (e.g. radio frequency ablation (RFA) or microwave ablation (MWA)). The addition of a TACE before thermal ablation reduces the perfusion induced “cooling” of the ablation zone and significantly increases local tumour control and survival when combined with thermal ablation in HCCs from 3.5 to 7 cm [7].

In recent years TACE spread to other secondary liver tumour, specially to liver metastasis from gastroenteropancreatic neuroendocrine tumors (GEP-NETs) and from colorectal cancers (CRC) [8, 9]. In GEP-NET either a transarterial bland embolisation (TAE) without chemotherapeutic agent or a cTACE with doxorubicin is performed in specialised centers. For CRC liver metastasis DEB-TACE using irinotecan as loading drug is the established technique for TACE.

**References**

1. Vogl TJ, Zangos S, Scholtz JE, Schmitt F, Paetzold S, Trojan J, et al. Chemosaturation with percutaneous hepatic perfusions of melphalan for hepatic metastases: experience from two European centers. RoFo: Fortschritte auf dem Gebiete der Rontgenstrahlen und der Nuklearmedizin. 2014;186(10):937-44.

2. de Baere T, Arai Y, Lencioni R, Geschwind JF, Rilling W, Salem R, et al. Treatment of Liver Tumors with Lipiodol TACE: Technical Recommendations from Experts Opinion. Cardiovasc Intervent Radiol. 2016;39(3):334-43.

3. Llovet JM, Real MI, Montana X, Planas R, Coll S, Aponte J, et al. Arterial embolisation or chemoembolisation versus symptomatic treatment in patients with unresectable hepatocellular carcinoma: a randomised controlled trial. Lancet. 2002;359(9319):1734-9.

4. Lo CM, Ngan H, Tso WK, Liu CL, Lam CM, Poon RT, et al. Randomized controlled trial of transarterial lipiodol chemoembolization for unresectable hepatocellular carcinoma. Hepatology. 2002;35(5):1164-71.

5. Lammer J, Malagari K, Vogl T, Pilleul F, Denys A, Watkinson A, et al. Prospective randomized study of doxorubicin-eluting-bead embolization in the treatment of hepatocellular carcinoma: results of the PRECISION V study. Cardiovasc Intervent Radiol. 2010;33(1):41-52.

6. Golfieri R, Giampalma E, Renzulli M, Cioni R, Bargellini I, Bartolozzi C, et al. Randomised controlled trial of doxorubicin-eluting beads vs conventional chemoembolisation for hepatocellular carcinoma. Br J Cancer. 2014;111(2):255-64.

7. Peng ZW, Zhang YJ, Chen MS, Xu L, Liang HH, Lin XJ, et al. Radiofrequency ablation with or without transcatheter arterial chemoembolization in the treatment of hepatocellular carcinoma: a prospective randomized trial. J Clin Oncol. 2013;31(4):426-32.

8. Del Prete M, Fiore F, Modica R, Marotta V, Marciello F, Ramundo V, et al. Hepatic arterial embolization in patients with neuroendocrine tumors. Journal of experimental & clinical cancer research : CR. 2014;33:43.

9. De Groote K, Prenen H. Intrahepatic therapy for liver-dominant metastatic colorectal cancer. World J Gastrointest Oncol. 2015;7(9):148-52.

### O9 Interventional radiology in paediatric oncology

#### Derek Roebuck

##### Department of Radiology, Great Ormond Street Hospital, London WC1N 3JH, UK

Despite widespread acceptance of ablation and other local interventional radiology (IR) procedures in adult oncology, the applications of IR in paediatric oncology have until recently been mostly restricted to biopsy, central venous access and certain forms of supportive care [1]. In many centres image-guided biopsy has essentially replaced surgical biopsy for the diagnosis of extracranial solid tumours in children.

Attempts to introduce newer therapeutic techniques into paediatric practice have been hindered by practical, legislative and cultural obstacles [2]. Although a 2014 systematic review found reports of only 28 children treated with ablation techniques for malignant or aggressive benign lesions [3], many more relevant publications have appeared since then. For the present, at least, the most likely application of such techniques is in children with recurrent (or perhaps unresectable) tumors. Other indications may appear as paediatric oncologists become more familiar with the value of these procedures. One such possibility would be the use of radiofrequency ablation or cryoablation as an alternative to partial nephrectomy for treatment of bilateral nephroblastoma (Wilms’ tumour).

Treatment of malignant liver tumours in children with chemoembolization (TACE) was investigated as long ago as the early 1990s, with promising results [4]. Three principal indications for TACE in children have been identified: conversion of unresectable tumours to resectability, as a bridge to transplantation, and as a part of palliation. Despite this, chemoembolization has not so far been incorporated into any major paediatric liver tumour trial.

Some other IR procedures have an occasional role in the palliative care of children with cancer [5]. These include techniques for the management of malignant pleural effusions or ascites, and methods for delivering analgesic drugs or nerve blocks.

**References**

1. Hoffer FA: **Interventional radiology in pediatric oncology.** *Eur J Radiol* 2005; **53**: 3-13.

2. Hoffer FA: **Interventional radiology; the future.** *Pediatr Radiol* 2011; **41** (Suppl 1): S201-S206.

3. Gómez Muñoz F, Patel PA, Stuart S, Roebuck DJ: **Systematic review of ablation techniques for the treatment of malignant or aggressive benign lesions in children.** *Pediatr Radiol* 2014; **44**: 1281-1289.

4. Malogolowkin MH, Stanley P, Steele DA, Ortega JA: **Feasibility and toxicity of chemoembolization for children with liver tumors.** *J Clin Oncol* 2000; **18**: 1279-1284.

5. Roebuck DJ: **Interventional radiology in paediatric palliative care.** *Pediatr Radiol* 2014; **44**: 12-17.

### O10 Image guided prostate interventions

#### Jurgen J. Fütterer

##### Department of Radiology and Nuclear Medicine, Radboud University Medical Center, Nijmegen, 6500HB, The Netherlands

With the widespread use of prostate-specific antigen screening and increasing life expectancy, more men are being diagnosed with localized, low-risk, low-grade prostate cancer [1]. As a result, men with localised prostate cancer and physicians who advise them face a difficult therapeutic dilemma: surveillance versus radical whole-gland therapy [2]. The available evidence from randomized controlled trials demonstrates that there is little to no difference between these choices in terms of overall and cancer-specific survival after a median of 10 years of follow-up [3]. Consequently, deferred treatment such as active surveillance is an appealing management solution which maximize the quality of life [4]. Conversely, radical treatment options, i.e. definitive radiotherapy or radical prostatectomy, come with considerable side effects such as erectile dysfunction and/or incontinence. Focal therapy is a strategy by which the overtreatment burden of the current prostate cancer pathway could be reduced [2]. This therapy concept has already been successfully applied to kidney [5], liver, breast, and lung cancer.

Focal therapy is an emerging local treatment option, which offers great hopes in term of decreased morbidity associated with standard whole-gland therapy without jeopardising cancer control [6]. The challenge of focal therapy is to treat the tumour, sparing the rest of the prostate, especially near the neurovascular bundles, bladder neck, rectum and the urethral sphincter, to minimize the potential morbidity. Concern regarding focal therapy has centered on the knowledge that prostate cancer is multifocal in origin. In prostate cancer, a larger dominant lesion is often accompanied by two or three smaller low-grade lesions. A hypothesis has emerged that the largest lesion in the prostate — the index lesion — drives disease progression [7]. The index lesion tends to be associated with the highest Gleason grade, harbours other pathological determinants of progression, and has been associated with lymph node metastases on genetic profiling. If the index lesion could be isolated with reasonable precision and treatment directed to it alone, then the oncological efficacy of whole-gland treatment might be matched while minimizing the risk of side effects.

**References:**

1. Stamey TA, Caldwell M, McNeal JE, Nolley R, Hemenez M, Downs J: **The prostate specific antigen era in the United States is over for prostate cancer: what happened in the last 20 years?** *The Journal of urology* 2004, **172**:1297-1301.

2. Valerio M, Ahmed HU, Emberton M, Lawrentschuk N, Lazzeri M, Montironi R, et al.: **The role of focal therapy in the management of localised prostate cancer: a systematic review**. *European urology* 2014, **66(4)**:732-751.

3. Wilt TJ, Brawer MK, Jones KM, Barry MJ, Aronson WJ, Fox S, et al.: **Radical prostatectomy versus observation for localized prostate cancer**. *N Engl J Med* 2012, **367(3)**:203-213.

4. Heidenreich A, Bellmunt J, Bolla M, Joniau S, Mason M, Matveev V, et al.: **EAU guidelines on prostate cancer. Part 1: screening, diagnosis, and treatment of clinically localised disease.** *European urology* 2011, **59(1)**:61-71.

5. Kutikov A, Kunkle DA, Uzzo RG: **Focal therapy for kidney cancer: a systematic review.** *Current opinion in urology* 2009, **19(2)**:148-153.

6. Ahmed HU, Pendse D, Illing R, Allen C, van der Meulen JH, Emberton M: **Will focal therapy become a standard of care for men with localized prostate cancer?** *Nat Clin Pract Oncol* 2007; **4**:632–642.

7. Ahmed HU: **The index lesion and the origin of prostate cancer**. *N Engl J Med* 2009, **361**:1704–1706.

### O11 Imaging cancer predisposition syndromes

#### Alexander J. Towbin

##### Cincinnati Children’s Hospital, Department of Radiology, 3333 Burnet Avenue, MLC 5031, Cincinnati, OH 45229, USA

Over the past five decades, researchers have begun to unravel many of the molecular pathways that lead to development of cancer. Through this work we have learned that 90 % of cancers occur as a result of an acquired somatic mutation while the remaining 10 % of cancers occur as a result of a hereditary, germline mutation [1]. Patients with a hereditary cancer predisposition syndrome often present younger in life than those patients with a somatic mutation [1].

To date, there are at least 114 known cancer predisposition syndromes [2]. The purpose of this talk is to describe several of the more common cancer predisposition syndromes including Li-Fraumeni syndrome, DICER 1 syndrome, Beckwith Wiedemann syndrome, tuberous sclerosis, and Down syndrome [3-7]. For each syndrome, the common tumours will be described and the current guidelines for screening will be discussed.

**References**

1. Saletta F, Dalla Pozza L, Byrne JA. Genetic causes of cancer predisposition in children and adolescents. Translational Pediatrics. 2015;4(2):67-75.

2. Rahman N. Realizing the promise of cancer predisposition genes. Nature. 2014 Jan 16;505(7483):302-8.

3. Agarwal R, Liebe S, Turski ML, Vidwans SJ, Janku F, Garrido-Laguna I, Munoz J, Schwab R, Rodon J, Kurzrock R, Subbiah V; Pan-Cancer Working Group. Targeted therapy for genetic cancer syndromes: Fanconi anemia, medullary thyroid cancer, tuberous sclerosis, and RASopathies. Discov Med. 2015 Feb;19(103):101-8.

4. Radhakrishnan R, Towbin AJ. Imaging findings in Down syndrome. Pediatr Radiol. 2014 May;44(5):506-21.

5. Anupindi SA, Bedoya MA, Lindell RB, Rambhatla SJ, Zelley K, Nichols KE, Chauvin NA. Diagnostic Performance of Whole-Body MRI as a Tool for Cancer Screening in Children With Genetic Cancer-Predisposing Conditions. AJR Am J Roentgenol. 2015 Aug;205(2):400-8.

6. Dehner LP, Messinger YH, Schultz KA, Williams GM, Wikenheiser-Brokamp K, Hill DA. Pleuropulmonary Blastoma: Evolution of an Entity as an Entry into a Familial Tumor Predisposition Syndrome. Pediatr Dev Pathol. 2015 Nov-Dec;18(6):504-11.

7. Mussa A, Molinatto C, Baldassarre G, Riberi E, Russo S, Larizza L, Riccio A, Ferrero GB. Cancer Risk in Beckwith-Wiedemann Syndrome: A Systematic Review and Meta-Analysis Outlining a Novel (Epi)Genotype Specific Histotype Targeted Screening Protocol. J Pediatr. 2016 Jun 29.

### O12 Chest and chest wall masses

#### Thierry AG Huisman

##### Radiology, Pediatrics, Neurology and Neurosurgery, Department of Imaging and Imaging Science, Johns Hopkins Bayview, 4940 Eastern Avenue, Baltimore, MD 21224, USA

Pediatric chest and chest wall masses are heterogeneous in etiology and presentation. Correct diagnosis of chest or chest wall masses is often challenging. Differentiation between malignant and benign processes including congenital abnormalities is of essential importance. Overall, primary pediatric pulmonary malignancies are uncommon. However, mediastinal masses and chest wall masses are not uncommon and should prompt thorough investigation.

In this interactive session we will use a case based approach to discuss various common and rare pediatric chest neoplasms as well as non-neoplastic differential diagnoses. In particular we will discuss the value of correlating the most likely primary site or epicenter of the lesion with the imaging characteristics as seen on radiography, ultrasonography, computed tomography, or magnetic resonance imaging as well as the age and gender of the child in narrowing down the differential diagnosis.

### O13 Abdominal masses: good or bad?

#### Anne MJB Smets (a.m.smets@amc.uva.nl)

##### Department of Radiology, Academic Medical Center, University of Amsterdam, Amsterdam, The Netherlands

Imaging plays an important role in the diagnosis of an abdominal mass in a child. Imaging studies need to be performed and interpreted in the light of the age of the child, clinical presentation and history, physical examination and laboratory results.

Ultrasound is a valuable technique for examining the pediatric abdomen and should always be the first imaging test to be performed. Both CT and MRI are modalities with a high burden for the child. The dose of ionizing radiation that comes with an abdominal CT is considerable. For abdominal MR, young children will need to be sedated or anaesthetized.

Abdominal malignancies in children are rare, with an incidence of 1-9 per million children per year. The most frequent malignant abdominal tumours of childhood are neuroblastoma, originating from the adrenal gland or anywhere from the sympathetic nervous system, nephroblastoma (Wilms’ tumour) from the kidney, hepatoblastoma from the liver [1-3]. Benign lesions of hemorrhagic, inflammatory or infectious origin as well as congenital malformations may mimic a malignancy and a malignant tumour may be mistaken for a benign lesion [4].

In some cases ultrasound imaging will demonstrate the benign nature of a mass, and exposure of the child to imaging modalities with a high burden will hence be unnecessary. Whenever ultrasound findings are inconclusive or suggestive of a malignancy, complementary cross-sectional imaging studies are necessary. CT or MRI should then be carried out with the highest possible yield of diagnostic information and with the ALARA principle in mind [5].

In order to avoid pitfalls, it is important to be aware of the complementarity of the different imaging modalities and to include all information in the conclusion of the report.

**References**

1. McCarville MB. **What MRI can tell us about neurogenic tumors and rhabdomyosarcoma**. Pediatric radiology. 2016;46(6):881-90.

2. Smets AM, de Kraker J. **Malignant tumours of the kidney: imaging strategy.** Pediatric radiology. 2010;40(6):1010-8.

3. Pugmire BS, Towbin AJ. **Magnetic resonance imaging of primary pediatric liver tumors**. Pediatric radiology. 2016;46(6):764-77.

4. Wessely K, Biassoni L, McHugh K. Pitfalls in paediatric oncology imaging. Cancer imaging : the official publication of the International Cancer Imaging Society. 2011;11:144-54.

5. Voss SD, Reaman GH, Kaste SC, Slovis TL. **The ALARA concept in pediatric oncology**. Pediatric radiology. 2009;39(11):1142-6.

### O14 Hepatobiliary MR contrast: enhanced liver MRI for HCC diagnosis and management

#### Giovanni Morana

##### Radiological Department, General Hospital Ca’ Foncello, Treviso Italy

Hepatocellular carcinoma (HCC) is the most frequent primary liver tumour (80 % - 90 %) and represents more than 5 % of all cancers. HCC is the endpoint of a serial transformation beginning from a dysplastic nodule, often triggered by chronic liver inflammation and cirrhosis. The progression from dysplastic nodule to HCC implies not only morphological changes but also vascular transformations, with process of neoangiogenesis leading to an increased number of unpaired arteries, progressive reduction of hepatocellular function [1].

Dynamic T1w during the arterial phase is of outmost importance for the detection of small HCCs [2]. Typically, HCCs demonstrate arterial enhancement and venous washout showing later hypointensity. However, HCCs smaller than 2 cm and well-differentiated HCCs frequently have atypical enhancement patterns [3]. In these situations, the use of liver-specific contrast agents (Gd-BOPTA or Gd-EOB-DTPA) can be helpful. While dynamic imaging of HCC with liver-specific MR contrast agents is similar to what observed with conventional CA, delayed hepatobiliary phase (HBP) imaging reveals a number of different enhancement patterns, with both iso-, hypo- and hyperintense patterns possible.

The signal intensity of HCCs on the HBP images has been recently considered as a potential imaging biomarker [4]. HBP hyper-intense HCCs have been reported to have histologic features related with favorable outcomes more frequently than HBP hypointense HCCs, showing higher grades of tumor differentiation, a lower rate of microvascular invasion [4], lower levels of expression of several kinds of poorer prognostic immunohistochemical markers [5].

The hepatobiliary phase images can increase the value of Gd-enhanced MRI in the diagnosis of early stage HCC. The use of liver specific contrast agents increase the sensitivity and accuracy for small HCCs either with Gd-EOB-DTPA [6] and with Gd-BOPTA [7].

**References**

1. Hanna RF, Aguirre DA, Kased N et al. Cirrhosis- associated hepatocellular nodules: correlation of histopathologic and MR Imaging features. RadioGraphics 2008; 28:747–769

2. Krinsky GA, Lee VS, Theise ND et al. Hepatocellular carcinoma and dysplastic nodules in patients with cirrhosis: prospective diagnosis with MR imaging and explantation correlation. Radiology 2001;219 (2):445-454.

3. Yoon SH, Lee JM, So YH, Hong SH, Kim SJ, Han JK, Choi BI. Multiphasic MDCT enhancement pattern of hepatocellular carcinoma smaller than 3 cm in diameter: tumor size and cellular differentiation. AJR Am J Roentgenol. 2009 Dec;193(6):W482-9).

4. Kitao A, Matsui O, Yoneda N, Kozaka K, Kobayashi S, Koda W, Gabata T, Yamashita T, Kaneko S, Nakanuma Y, Kita R, Arii S. Hypervascular hepatocellular carcinoma: correlation between biologic features and signal intensity on gadoxetic acid-enhanced MR images. Radiology. 2012;265:780–789.

5. Yoneda N, Matsui O, Kitao A, Kita R, Kozaka K, Koda W, Kobayashi S, Gabata T, Ikeda H, Nakanuma Y. Hypervascular hepatocellular carcinomas showing hyperintensity on hepatobiliary phase of gadoxetic acid-enhanced magnetic resonance imaging: a possible subtype with mature hepatocyte nature. Jpn J Radiol. 2013;31(7):480–490.

6. Haradome H, Grazioli L, Tinti R, Morone M, Motosugi U, Sano K, Ichikawa T, Kwee TC, Colagrande S. Additional value of gadoxetic acid-DTPA-enhanced hepatobiliary phase MR imaging in the diagnosis of early-stage hepatocellular carcinoma: comparison with dynamic triple-phase multidetector CT imaging. J Magn Reson Imaging. 2011 Jul;34(1):69-78.)

7. Morana G, Grazioli L, Kirchin MA, Bondioni MP, Faccioli N, Guarise A, Schneider G. Solid Hypervascular Liver Lesions: Accurate Identification of True Benign Lesions on Enhanced Dynamic and Hepatobiliary Phase Magnetic Resonance Imaging After Gadobenate Dimeglumine Administration. Invest Radiol. 2011 Apr;46(4):225-39

### O15 Role of US elastography and multimodality fusion for managing patients with chronic liver disease and HCC

#### Jeong Min Lee

##### Department of Radiology, Seoul National University Hospital, Seoul, Korea

Ultrasound plays an important role for monitoring development of liver cirrhosis and for screening of hepatocellular carcinoma (HCC). Recent developments of US including ultrasound elastography (USE) and multimodality fusion imaging may play an important role for management of patients with chronic liver disease and liver cirrhosis [1, 2]. Prognosis of patients with chronic liver disease is determined by the extent and progression of liver fibrosis, which may ultimately lead to hepatocellular carcinoma (HCC) [3]. Although liver biopsy (LB) is regarded as the gold standard to estimate the extent of liver fibrosis, it has several limitations, the foremost being its invasiveness and sampling bias due to its limited sampling volume. Among the several non-invasive methods for assessing liver fibrosis recently developed, MR elastography and USE techniques including transient elastography (TE), point or two-dimensional shearwave elastography (SWE) provide an accurate representation of the extent of liver fibrosis [2, 4]. Recent studies have demonstrated the usefulness of USE for assessing the risk of HCC development and HCC recurrence after curative treatment, and developed novel models to calculate the risk of HCC development based on stiffness values. In addition, with the technical development of ultrasonography (US), electromagnetic tracking-based fusion imaging of real-time US and computed tomography/magnetic resonance (CT/MR) images has been used for percutaneous hepatic intervention such as biopsy and radiofrequency ablation (RFA) [5]. Real-time multimodality fusion imaging can enhance lesion detectability and reduce the false positive detection of focal hepatic lesions with poor sonographic conspicuity, and may improve efficacy of the percutaneous RFA of liver tumors using an overlapping ablation technique or multi-electrode technique [1]. Volumetric and fusion imaging should improve the therapeutic management of malignant liver lesions and particularly percutaneous guidance of thermo-ablation procedures.

**References**

1. Lee MW. Fusion imaging of real-time ultrasonography with CT or MRI for hepatic intervention. Ultrasonography (Seoul, Korea). 2014;33(4):227-39.

2. Yoon JH, Lee JM, Joo I, et al. Hepatic fibrosis: prospective comparison of MR elastography and US shear-wave elastography for evaluation. Radiology. 2014;273(3):772-82.

3. Park MS, Han KH, Kim SU. Non-invasive prediction of development of hepatocellular carcinoma using transient elastography in patients with chronic liver disease. Expert review of gastroenterology & hepatology. 2014;8(5):501-11.

4. Chang W, Lee JM, Yoon JH, et al. Liver Fibrosis Staging with MR Elastography: Comparison of Diagnostic Performance between Patients with Chronic Hepatitis B and Those with Other Etiologic Causes. Radiology. 2016;280(1):88-97.

5. Minami Y, Kudo M. Ultrasound fusion imaging of hepatocellular carcinoma: a review of current evidence. Digestive diseases (Basel, Switzerland). 2014;32(6):690-5.

### O16 Opportunities and challenges in imaging metastatic disease

#### Hersh Chandarana

##### Department of Radiology, New York University School of Medicine, New York 10016, USA

Positron emission tomography (PET) and magnetic resonance (MR) imaging until recently has been performed by separate PET and MR devices with temporal delay between these two acquisitions. However, various recent hardware solutions have been developed by different vendors which permit simultaneous or near simultaneous PET and MR acquisition. However, the clinical translation of this modality for oncologic imaging requires not only identifying the appropriate clinical indications, but also understanding various components involved in establishing a PET/MR service which include physical installation of the system, equipment safety, clinical workflow, technician and physician training, and monetary reimbursement.

The current and potential clinical indications for imaging of metastatic disease can be broadly classified as follows:

1. Simultaneous local and distant staging of cancers such as rectal cancer and gynecologic malignancies. Here high spatial resolution of MRI provides information about local extent of the disease and PET is used predominantly for distant staging.
2. Problem solving for potential metastatic disease such as for small liver lesions, small lymph nodes, or bone marrow involvement. Additional information provided by MRI and PET can better characterize lesions and improve confidence in diagnosing presence or absence of metastatic disease.
3. Assessment of treatment response. Use of quantitative MR and PET information can potentially provide synergistic information in assessing treatment response.

To address these clinical need there are number of operational considerations such as:

1. Protocol optimisation.
2. Workflow in scanning and interpretation of studies.

Some of the technical challenges and limitations that need to be considered include:

1. Limitation of MRI for lung lesion detection.
2. Attenuation correction.
3. Registration of free-breathing PET and breath-hold thoacoabdominal MR data.

While FDG PET/CT remains the workhorse for diagnosis and management of oncologic diseases, early experience shows that PET/MR may have a complementary role. PET/MR could potentially play a significant role in diagnosis and management algorithms of several malignancies.

### O17 Diagnosis, treatment monitoring, and follow-up of lymphoma

#### Marius E. Mayerhoefer^1^, Markus Raderer^2^, Alexander Haug^1^

##### ^1^Dept. of Biomedical Imaging and Image-guided Therapy, Medical University of Vienna, Vienna, 1090, Austria; ^2^Dept. of Internal Medicine I, Medical University of Vienna, Medical University of Vienna, Vienna, 1090, Austria

###### **Correspondence:** Marius E. Mayerhoefer (marius.mayerhoefer@meduniwien.ac.at) – Dept. of Biomedical Imaging and Image-guided Therapy, Medical University of Vienna, Vienna, 1090, Austria

Integrated PET/MR (positron emission tomography/magnetic resonance imaging) devices have only been recently introduced into routine clinical practice. Compared to PET/CT (computed tomography), the most widely utilised hybrid imaging technique at present, for which the total scan duration is only about 20-25 min, the scan duration is rather long for PET/MR, with 45-60 min for a whole-body examination. Therefore, a justification for the use of PET/MR, which is more cost intensive while providing a lower patient throughput, is required.

For malignant lymphoma, a heterogeneous family of cancers for which PET/MR has already been evaluated, the most obvious advantage, compared to PET/CT, is the low radiation exposure (about 6-8 mSv, compared to at least 20 mSv for a fully diagnostic, contrast-enhanced PET/CT examination). This topic is of relevance because lymphoma is one of the more common malignancies among children and adolescents, for whom life-long monitoring may be required, and for whom the risk of development of secondary, radiation-induced neoplasms must therefore be minimised.

A less frequently recognised advantage of PET/MR is the fact that DWI (diffusion-weighted imaging) can be incorporated in the examination. In lymphoma, DWI – even as a stand-alone whole-body technique – has shown to be only moderately inferior to [18F]-FDG-PET/CT – both in terms of pre-therapeutic staging and post-therapeutic restaging/treatment response assessment [1, 2]. Similar to the quantification of glucose metabolism on [18F]-FDG-PET, DWI offers the ability to quantify diffusion restriction by means of apparent diffusion coefficients, and thus, indirectly quantify cell density. Notably, DWI has also performed particularly well in some of the lymphoma subtypes for which [18F]-FDG-PET is currently not recommended by the ICML (International Conference on Malignant Lymphoma), which includes marginal zone lymphomas, SLL/CLL (small lymphocytic lymphoma/chronic lymphocytic leukemia), Morbus Waldenström, and mycosis fungoides [3]. It is thus not surprising that the current literature suggests not just a non-inferiority of [18F]-FDG-PET/MR, compared to [18F]-FDG-PET/CT, but a moderate superiority, at least when the entire spectrum of lymphomas is taken into account [4].

Another advantage of PET/MR lies in the fact that dynamic or multiple time-point PET can be easily integrated into the work-flow. Delayed time-point [18F]-FDG-PET, for instance, has been shown to improve the detection of slowly-growing, indolent lymphomas with no, or low, FDG uptake, such as MALT lymphoma [5], the third most-common type of Non-Hodgkin lymphoma.

With regard to MALT lymphoma, SLL/CLL, and possibly, several other lymphoma subtypes for which [18F]-FDG is of limited value, novel PET tracers might be used in the context of PET/MR. This includes [68Ga]-Pentixafor, a radiotracer that specifically attaches to CXCR4 chemokine receptors [6]. CXCR4 receptors are overexpressed on the cell surfaces of different tumours, including those derived from the lympho-proliferative system, and regulate, among other things, cell migration. In an ongoing study presently performed at our institution, [68Ga]-Pentixafor has, so far, shown a very high sensitivity for detection of MALT lymphomas.

**References**

1. Mayerhoefer ME, Karanikas G, Kletter K, Prosch H, Kiesewetter B, Skrabs C, Porpaczy E, Weber M, Pinker-Domenig K, Berzaczy D, Hoffmann M, Sillaber C, Jaeger U, Müllauer L, Simonitsch-Klupp I, Dolak W, Gaiger A, Ubl P, Lukas J, Raderer M: **Evaluation of diffusion-weighted MRI for pretherapeutic assessment and staging of lymphoma: results of a prospective study in 140 patients.** *Clin Cancer Res* 2014;**20**:2984-2993.

2. Mayerhoefer ME, Karanikas G, Kletter K, Prosch H, Kiesewetter B, Skrabs C, Porpaczy E, Weber M, Knogler T, Sillaber C, Jaeger U, Simonitsch-Klupp I, Ubl P, Muellauer L Dolak W, Lukas J, Raderer M: **Evaluation of diffusion-weighted magnetic resonance imaging for follow-up and treatment response assessment of lymphoma: results of an** 18F**-FDG-PET/CT-controlled prospective study in 64 patients.** *Clin Cancer Res* 2015;**21**:2506-2513.

3. Cheson B, Fisher RI, Barrington SF, Cavalli F, Schwartz LH, Zucca E, Lister TA: **Recommendations for initial evaluation, staging, and response assessment of Hodgkin and non-Hodgkin lymphoma: the Lugano classification.** *J Clin Oncol* 2014;**32**:3059-3068.

4. Giraudo G, Raderer M, Karanikas G, Weber M, Kiesewetter B, Dolak W, Simonitsch-Klupp I, Mayerhoefer ME: **[**18F**]-FDG-PET/MR in lymphoma: comparison with [**18F**]-FDG-PET/CT and with the addition of diffusion-weighted MRI**. *Invest Radiol* 2016;**51**:163-169.

5. Mayerhoefer ME, Giraudo C, Senn D, Hartenbach M, Weber M, Rausch I, Kiesewetter B, Herold CJ, Hacker M, Pones M, Simonitsch-Klupp I, Müllauer L, Dolak W, Lukas J, Raderer M: **Does delayed-time-point imaging improve F-18-FDG-PET in patients with MALT lymphoma? Observations in a series of 13 patients**. *Clin Nucl Med* 2016;**41**:101-10

6. Herrmann K, Lapa C, Wester HJ, Schottelius M, Schiepers C, Eberlein U, Bluemel C, Keller U, Knop S, Kropf S, Schirbel A, Buck AK, Lassmann M: **Biodistribution and radiation dosimetry for the chemokine receptor CXCR4-targeting probe 68Ga-pentixafor**. *J Nucl Med* 2015;**56**:410-416.

### O18 Managing high-risk and advanced prostate cancer

#### Matthias Eiber (matthias.eiber@tum.de)

##### Department of Nuclear Medicine, Klinikum rechts der Isar, Technical University of Munich, Munich, Germany

Currently national and international guidelines for imaging procedures for high-risk and advanced prostate cancer (PCa) include abdomino-pelvic cross sectional imaging, multiparametric prostate MRI, bone scintigraphy and in the case of therapy monitoring of mCRPC whole body cross-sectional imaging mainly by means of computed tomography. Positron emission tomography (PET) has became increasingly important in the work-up of prostate cancer. In the past, the use was mainly limited to radiolabbeled Choline-derivates which showed considerable limitations and did not always meet the diagnostic needs.

Recently, a ^68^Gallium-labelled ligand of the prostate-specific membrane antigen (^68^Ga-PSMA) has been introduced in PET-imaging of PCa with first promising results. Due to relatively exclusive expression of PSMA in prostatic tissue as well as increased expression in PCa ^68^Ga-PSMA was reported to exhibit a favourable lesion to background ratio compared to presently used choline- or fluorodesoxyglucose-based PET examinations. Together with the novel development of combined PET/MR, the combination of excellent morphological detail, multiparametric functional information and molecular PET data might lead to a significant improvement in detection and staging of PCa and thus may help to optimise oncological treatment. The talk encompasses:

Teaching and learning contents of the talk will include:

1. The molecular basis of prostate cancer imaging targeting the prostate-specific-membran antigen (PSMA), review of the various PSMA-tracers [1] [2].
2. The diagnostic performance and potential role of PSMA PET/CT and PET/MR for high-risk primary and advanced prostate cancer [3] [4].
3. Comparison of the use of PSMA PET-imaging with conventional imaging as included in guidelines [3,5].
4. Discussion of non-routine applications (e.g. biopsy targeting, radioguided surgery, radioreceptor therapy using Lu177-PSMA) [6] [7].

**References**

1. Maurer T, Eiber M, Schwaiger M, Gschwend JE. **Current use of PSMA-PET in prostate cancer management.** Nat Rev Urol. April 2016;13(4):226–35.

2. Lütje S, Heskamp S, Cornelissen AS, Poeppel TD, Broek SAMW van den, Rosenbaum-Krumme S, u. a. **PSMA Ligands for Radionuclide Imaging and Therapy of Prostate Cancer: Clinical Status.** Theranostics. 2015;5(12):1388–401.

3. Maurer T, Gschwend JE, Rauscher I, Souvatzoglou M, Haller B, Weirich G, u. a. **Diagnostic Efficacy of (68)Gallium-PSMA-PET compared to Conventional Imaging in Lymph Node Staging of of 130 consecutive Patients with Intermediate to High-Risk Prostate Cancer**. J Urol. 9. Dezember 2015;

4. Eiber M, Weirich G, Holzapfel K, Souvatzoglou M, Haller B, Rauscher I, u. a. **Simultaneous 68Ga-PSMA HBED-CC PET/MRI Improves the Localization of Primary Prostate Cancer.** Eur Urol [Internet]. [zitiert 19. Januar 2016]; Verfügbar unter: http://www.sciencedirect.com/science/article/pii/S0302283816000117

5. Pyka T, Weirich G, Einspieler I, Maurer T, Theisen J, Hatzichristodoulou G, u. a. **[68Ga]PSMA-HBED PET for differential diagnosis of suspicious lung lesions in patients with prostate cancer.** J Nucl Med Off Publ Soc Nucl Med. 19. November 2015;

6. Maurer T, Weirich G, Schottelius M, Weineisen M, Frisch B, Okur A, u. a. P**rostate-specific membrane antigen-radioguided surgery for metastatic lymph nodes in prostate cancer.** Eur Urol. September 2015;68(3):530–4.

7. Heck MM, Retz M, D’Alessandria C, Rauscher I, Scheidhauer K, Maurer T, u. a. **Systemic radioligand therapy with (177)Lu-PSMA-I&T in patients with metastatic castration-resistant prostate cancer**. J Urol. 8. März 2016;

### O19 Immunotherapy: imaging challenges

#### Bernhard Gebauer (bernhard.gebauer@charite.de)

##### Department of Radiology, Charité, Campus Virchow-Klinikum, Augustenburger Platz 1, 13353 Berlin, Germany

Traditional chemotherapy is directly introducing tumour cell death and later a shrinkage of tumour masses throughout the body. So response evaluation criteria, like WHO, RECIST or Lugano criteria, used for evaluation of response to chemotherapy rely on tumour shrinkage as a sign of efficacy [1-4]. Additionally in these criteria the appearance of new lesions or progressing non-target lesions is indicating progress of disease independent of the changes in the target lesions.

Ipilimumab, a monoclonal antibody that blocks cytotoxic T-lymphocyte-associated antigen 4 (CTLA 4), was the first immunotherapeutic agent that showed in clinical phase II trials survival benefits and successful tumour therapy in patients with advanced malignant melanoma [5]. In the study treating malignant melanoma it was shown that superficial and organ metastasis in some patients even increase in size or new lesions appear, whereas these patients later show very good response and survival benefit in the following course of the study. Following traditional response criteria these patients would have been assessed as progressive disease (PD) and excluded from the study (or dropped out from the progression free survival (PFS) group). This increase of size and appearance of the lesions is thought to be a result of lymphocyte and macrophage invasion into the tumour and is called “pseudoprogression”.

Using the results from the mentioned study new criteria, immune-related response criteria (irRC), were defined by Wolchok et al. [5]. These criteria base on bi-dimensional target lesion measurements analogue to WHO criteria. In contrast to other criteria new tumour lesions not directly trigger progressive disease (PD). The bi-dimentional measurements of new appearing lesions will be included into the sum of product tumour diameters (SPD) of the defined target lesions at baseline. Additionally every progressive disease (PD) must be confirmed by a repeated imaging with minimum 4 weeks interval. Both new rules should reduce the pseudoprogression in the tumour response assessment in immunotherapy.

In the last years these new rules have been integrated into the mono-dimensional RECIST criteria to evaluate immunotherapy. For these new mono-dimensional criteria no commonly accepted term exists. This modification is called immune-related RECIST (irRECIST), immune-modified RECIST, modified RECIST (mRECIST) or irRC 2014. Personally I prefer the term immune-related RECIST (irRECIST), because it best reflects the immunotherapeutic modification of the established RECIST system.

In the last years many new immunotherapeutic agents have been investigated in clinical studies, like pembrolizumab, nivolumab (anti-programmed cell death protein 1 (anti-PD1) antibodies), atezolizumab (anti-PD-L1 (programmed cell death-ligand 1)), ipilimumab (anti-CTLA 4), alemtuzumab (anti CD52), ofatumumab and rituximab (anti-CD20).

Current studies are investigating these checkpoint inhibitors of the immune system in colorectal cancer, malignant melanoma, breast cancer, non-small-cell lung carcinoma, bladder cancer, renal cell carcinoma, lymphoma and leukaemia.

**References**

1. Miller AB, Hoogstraten B, Staquet M, Winkler A. Reporting results of cancer treatment. Cancer. 1981;47(1):207-14.

2. Therasse P, Arbuck SG, Eisenhauer EA, Wanders J, Kaplan RS, Rubinstein L, et al. New guidelines to evaluate the response to treatment in solid tumors. European Organization for Research and Treatment of Cancer, National Cancer Institute of the United States, National Cancer Institute of Canada. J Natl Cancer Inst. 2000;92(3):205-16.

3. Eisenhauer EA, Therasse P, Bogaerts J, Schwartz LH, Sargent D, Ford R, et al. New response evaluation criteria in solid tumours: revised RECIST guideline (version 1.1). Eur J Cancer. 2009;45(2):228-47.

4. Cheson BD, Fisher RI, Barrington SF, Cavalli F, Schwartz LH, Zucca E, et al. Recommendations for initial evaluation, staging, and response assessment of Hodgkin and non-Hodgkin lymphoma: the Lugano classification. J Clin Oncol. 2014;32(27):3059-68.

5. Wolchok JD, Hoos A, O’Day S, Weber JS, Hamid O, Lebbe C, et al. Guidelines for the evaluation of immune therapy activity in solid tumors: immune-related response criteria. Clinical cancer research: an official journal of the American Association for Cancer Research. 2009;15(23):7412-20.

### O20 RECIST and RECIST 1.1

#### Andrea Rockall

##### Radiology Department, Hammersmith Hospital, London, W12 0HS, UK

The measurement of response following cancer treatment is critical in order to ensure the most appropriate and optimal management for each patient. This applies to daily practice as well as to patients taking part in clinical trials. The main difference between these two settings is that the recording of response in the trial setting needs to be done in a highly uniform and rigorous manner that is standardised and reproducible.

The detection of response, whether for clinical trial reporting or for daily practice, requires a few important pieces of information:

1. We need to know the date of baseline scan immediately prior to a new treatment being started.
2. We need to know what disease was present at the time of the baseline scan. In the case of clinical trial reporting, we need to know which target lesions were selected at baseline.
3. We need to know if there were any important events that could alter the scan appearances (for example drainage of ascitic fluid or interval surgical resection during the course of treatment).
4. We need to be aware of the findings on each scan from baseline to the time of the scan that is currently being reported, in order to be certain that the nadir (the smallest recorded diameter of target lesions) is correctly identified.

For the purposes of clinical trial reporting, it is essential to know which reporting criteria are being used for the trial. Most trials use RECIST 1.1; this updated version addresses some of the issues with the first RECIST, such as defining the measurement of lymph nodes. However, some trials do still use the original RECIST, usually in diseases that have a predominance of metastases in one organ, such as the liver in gastrointestinal neuroendocrine tumours. In this case, being restricted to only two lesions in one organ becomes restrictive and potentially not representative of changes that may be occurring.

The initial selection of target lesions at baseline lays the foundation for the future reports. Clear guidance on the selection of lesions should be adhered to. The target selection rules will be reviewed in the course of the workshop. Clear recording of the selected target lesions and the recording of the sum of target lesions will help in future reports.

A very helpful aspect of the RECIST 1.1 paper is the section on “Frequently asked questions”. Several of these more challenging aspects of trial reporting will be reviewed, together with examples.

### O21 Challenges of RECIST in oncology imaging basics for the trainee and novice

#### Aslam Sohaib

##### The Royal Marsden NHS Foundation Trust, Sutton, London, UK

Response Evaluation Criteria for Solid Tumours (RECIST) guidelines were introduced in 2000 and updated in 2009 to provide a standardised method for assessing response to treatments [1,2]. Tumour burden is measured using sum of the diameters with uni-dimensional measurements. The response categories are those of complete response, partial response, stable disease and progressive disease. Though the RECIST criteria are intended for use in the clinical trial setting, onocologist increasingly rely on RECIST based measurements to make clinical management and therapeutic decisions in daily clinical practice.

RECIST guidelines are therefore widely employed, however, they have well recognised limitations and pitfalls. Tumours that are irregular, or show diffuse infiltration or poorly visualised eg with fatty liver, can all be difficult to measure in a reliable and reproducible way. Tumour with non-spherical growth pattern can be difficult to serially follow up and assess response. The categories for the response criteria are arbitrarily defined and may not correlate with clinical outcome. Morphological characteristics and tumour heterogeneity are not taken into consideration. Functional or physiological change with response is not assessed / measured in RECIST. Reliance of response is based exclusively on tumour size and clinical beneficial chemotherapeutic effects may occur without reduction in tumour size eg many new targeted therapies have anti-angiogenic effects and result in symptomatic improvement in patients. Similar immue mediated response may artificially result in short term increase in tumour size or small new lesions. Treatment effects such as necrosis, cystic change and haemorrhage can result in artificial change in tumour size. Mixed and differential response is simplified in the response assessment in RECIST. Bone disease without significant soft tissue and cystic tumour it can be difficult to assess change with the tumour.

The limitations in RECIST guidelines have led to the publications and developments in modified RECIST and other response criteria to overcome these shortfalls.

**References**

1. Therasse, P., et al., *New guidelines to evaluate the response to treatment in solid tumors. European Organization for Research and Treatment of Cancer, National Cancer Institute of the United States, National Cancer Institute of Canada.* J Natl Cancer Inst, 2000. **92**(3): p. 205-16.

2. Eisenhauer, E.A., et al., *New response evaluation criteria in solid tumours: revised RECIST guideline (version 1.1).* Eur J Cancer, 2009. **45**(2): p. 228-472.

3. Tirkes T, Hollar MA, Tann M, Kohli MD, Akisik F, Sandrasegaran K. *Response criteria in oncologic imaging: review of traditional and new criteria.* Radiographics. 2013. **33**(5):1323-41

### O22 Lymphoma: PET for interim and end of treatment response assessment: a users’ guide to the Deauville Score

#### Victoria S Warbey

##### King’s College London and Guy’s & St Thomas’ PET Centre, Division of Imaging Sciences and Biomechanical Engineering, King’s College London, London, SE1 7EH, UK

**Introduction:** The Deauville Score (DS) (also referred to as the Deauville criteria) is a standardised five-point scale developed to assess treatment response in lymphoma. The scale originated from a scoring system developed at St Thomas’ Hospital and was at that time named the SELCN (South East London Cancer Network) Score. Following initial work confirming high concordance of the reporting criteria between reporters, particularly with respect to use in clinical trials [1], the scoring system was adopted as the preferred method for reporting of response assessment in lymphoma at the First International Workshop on PET in Lymphoma in Deauville, France, in 2009 [2]. Its use has subsequently been validated in Hodgkin, diffuse large B-cell and follicular lymphomas [3,4,7].

Since then the DS has been increasingly used in clinical practice and in national and international trials as a simple, quick and reproducible method to assess response to treatment in lymphoma, with good interobserver agreement reported in several studies [5, 6, 7]. It has also been shown to have predictive and prognostic significance.

**How to use the Deauville Score:** To use the DS, the reader makes an initial visual assessment to identify the residual area(s) of focal uptake at original sites of disease, in comparison to a pretreatment baseline scan and measures the maximum standardised uptake value (SUVmax) of these areas. The reader then draws two background regions of interest. The first is drawn within the aortic arch (taking care to exclude vessel walls and associated calcification) and represents mediastinal blood pool. A second region is drawn in a large region of normal liver to determine the normal liver SUVmax.

The five-point scale scores the most intense uptake in a site of initial disease as:

1. no uptake.
2. uptake ≤ mediastinal blood pool.
3. uptake > mediastinal blood pool but ≤ liver.
4. uptake moderately higher than liver*.
5. uptake markedly higher than liver and/or new lesions**.

X new areas of uptake unlikely to be related to lymphoma.

*moderately is uptake up to 3 times greater than the SUVmax in a large region of normal liver.

**markedly is uptake greater than 3 times the SUVmax in a large region of normal liver.

**When to use the Deauville Score:** Recently published guidance recommends that the DS is used for reporting response assessment both at interim and end of treatment [8]. Whilst the majority of lymphomas are FDG-avid, published experience is predominantly with Hodgkin, diffuse large B-cell and follicular lymphomas. Furthermore, in specific circumstances, there is now evidence that interim PET assessed using the DS can be used to guide response adapted therapy [9, 10].

**Reporting template:** The presentation will also introduce the idea of a reporting template for response assessment in lymphoma incorporating the ideas discussed in the talk.

**Acknowledgements**

I am grateful to all my colleagues at PET Imaging Centre at St Thomas’, in particular Professor Sally Barrington for her invaluable guidance and expertise.

**References**

1. Barrington SF, Qian W, Somer EJ, et al: **Concordance between four European centres of PET reporting criteria designed for use in multicentre trials in Hodgkin lymphoma.** *Eur J Nucl Med Mol Imaging* 2010, **37**:1824-1833.

2. Meignan M, Gallamini A, Haioun C: **Report on the First International Workshop on Interim-PET-Scan in Lymphoma.** *Leuk lymphoma* 2009, **50**:1257-60.

3. Biggi A, Gallamini A, Chauvie S, et al. **International Validation study for interim PET in ABVD-treated advanced-stage hodgkin lymphoma: interpretation criteria and concordance rate among reviewers.** *J Nucl Med* 2013, **54:**683-90.

4. Trotman J, Luminari S, Bouseetta s, et al. **Prognostic value of PET-CT after first-line therapy in patients with follicular lymphoma: a pooled analysis of central scan review in three multicentre studies.** *Lancet Haematol* 2014, **1** e17-e27.

5. Le Roux PY, Gastinne T, Le Gouill S, et al: **Prognostic value of interim FDG PET/CT in Hodgkin’s lymphoma patients treated with interim response-adapted strategy: comparison of International Harmonization Project (IHP), Gallamini and London criteria.** *Eur J Nucl Med Mol Imaging* 2011, **38**:1064-71.

6. Furth C, Amthauer H, Hautzel H, et al: **Evaluation of interim PET response criteria in paediatric Hodgkin’s lymphoma-results for dedicated assessment criteria in a blinded dual-centre read.** Ann Oncol 2011, **22**:1198-1203.

7. Itti E, Meignan M, Berriolo-Riedinger A, et al: **An international confirmatory study of the prognostic value of early PET/CT in diffuse large B-cell lymphoma: comparison between Deauville criteria and DeltaSUVmax.** *Eur J Nucl Med Mol Imaging* 2013, **40:**1312-20.

8. Barrington SF, Mikhaeel NG, Kostakoglu L, et al. **The role of imaging in the staging and response assessment of lymphoma: consensus of the ICML Imaging Working Group.** *J Clin Oncol* 2014, **32:**3048-3058.

9. Johnson P, Federico M, Kirkwood A et al. **Adapted Treatment Guided by Interim PET-CT Scan in Advanced Hodgkin’s Lymphoma.** *N Engl J Med* 2016, **374**:2419-29.

10. Radford J, Illidge T, Counsell N, et al. **Results of a Trial of PET-Directed Therapy for Early-Stage Hodgkin’s Lymphoma.** *N Engl J Med* 2015, **372:**1598-607.

### O23 Available resources

#### Hebert Alberto Vargas

##### Department of Radiology, Memorial Sloan Kettering Cancer Center. New York, NY, USA

There is no doubt that imaging is a central tool in contemporary cancer care. From staging to assessing treatment response and follow-up, it would be difficult to imagine a patient with cancer in 2016 who has not undergone some form of imaging as part of their disease evaluation. This paradigm has solidified the place of oncologic imaging as a sub-specialty, but it has also lead to challenges related to augmenting the knowledge of those practicing it in areas that are not classically considered mainstream radiology. These continuously evolving topics are definitely relevant to oncologic imaging, such as molecular biology, genomics, proteomic and immunotherapy, just to name a few. All these have direct or indirect implications on the imaging findings or their interpretation, and as such should be part of the opportunities for lifelong learning. In this session, we will discuss resources for increasing radiologists’ knowledge of these topics.

### O24 ICIS e-portal and the online learning community

#### Dow-Mu Koh

##### Royal Marsden Hospital, Sutton, Surrey, SM2 5PT, UK

Life long learning in medicine is a process of continuously scrutinising our practice, recognising gaps in our understanding and actively acquiring new knowledge to ensure that we are practicing medicine to the highest professional standards. Life long learning is self-directed, personal, active, participatory and reflective. However, the busy working life and competing demands for our time may make it difficult to realise these aims. The key barriers to success include difficulty with personal reflection, environment strain, competing demands, difficulty with goal generation, and problems with plan generation and implementation. In addition, lack of access to high-quality learning materials may also impede progress.

E-learning is increasingly recognised as an effective way to deliver learning material for continuous medical education. As Oncologic Imaging is an emerging sub-speciality discipline, there are few sites dedicated to cancer imaging. The ICIS e-portal aims to address this gap by providing high-quality carefully curated learning material to the Society Membership. This will include dedicated online cancer imaging course, cases crowd-sourced from the ICIS Online Learning Community and development of the ICIS Ring of Excellence case archives.

### O25 Benign lesions that mimic pancreatic cancer

#### Jay P Heiken (heikenj@mir.wustl.edu)

##### Mallinckrodt Institute of Radiology, Washington University School of Medicine, St. Louis, MO 63110, USA

Chronic inflammatory processes involving the pancreas, normal anatomic variants and some benign neoplasms can mimic pancreatic malignancy. Some of these processes are discussed below.

*Intrapancreatic splenule* appears as a round enhancing mass within the tail of the pancreas. Because of its vascularity it can be mistaken for a pancreatic neuroendocrine tumor. One of the keys to diagnosis is recognition that the mass has enhancement, attenuation and/or signal intensity characteristics that parallel the spleen on all image acquisitions. The diagnosis can be confirmed with a technetium 99 m heat-damaged red blood cell scan, which demonstrates radiotracer uptake within the mass.

*Mass-forming chronic pancreatitis*, particularly *autoimmune pancreatitis*, frequently is misdiagnosed as pancreatic adenocarcinoma or a neuroendocrine tumour. Most commonly mass-forming chronic pancreatitis is isoattenuating/isointense during both the pancreatic and hepatic parenchymal phases of contrast enhancement. In contradistinction, pancreatic adenocarcinoma typically is hypoattenuating/hypointense during both enhancement phases; however, approximately 10 % of pancreatic adenocarcinomas are isoattenuating/isointense during both phases. On MR cholangiopancreatography (MRCP) the pancreatic duct within the mass-forming pancreatitis may be visible but narrowed (duct-penetrating sign), whereas the duct within pancreatic carcinoma often is occluded. One study has shown the duct-penetrating sign to be 94 % accurate in distinguishing the two entities. Elevation of serum IgG4 is the best serological marker for autoimmune pancreatitis (sensitivity 73-75 %; specificity 93-95 %); however, approximately 10 % of patients with pancreatic cancer may have elevated IgG4.

Pancreatic *serous cystadenoma* is a benign mass that consists of numerous tiny cysts separated by glandular tissue and fibrous stroma. On CT it appears as a well-circumscribed hypoattenuating mass with varying degrees of contrast enhancement, depending on the size of the cysts and the proportion of cystic to glandular tissue. In most cases it is not difficult to distinguish this multicystic lesion from a solid pancreatic neoplasm; however a small proportion of serous cystadenomas consist largely of glandular tissue and fibrous septa with only a small proportion of tiny cysts. Such lesions appear hypervascular and may mimic a pancreatic neuroendocrine tumor. Clues to the diagnosis include precontrast attenuation value within the range of fluid and the presence of small cystic areas with the enhancing mass. Heavily T2 weighted images may help demonstrate the microcystic nature of the mass in some cases. In addition, this diagnosis should be considered if the mass is found incidentally in an elderly individual or a patient with von Hippel Lindau disease.

*Focal fatty replacement of the pancreas* occasionally can mimic a pancreatic tumor, appearing as a focal hypoattenuating area on CT. The most common location is the anterior portion of the pancreatic head. Clues to the correct diagnosis include triangular shape and absence of mass effect or ductal obstruction. The presence of fat can be confirmed by demonstrating reduction in signal intensity on the opposed phase of chemical shift MR imaging.

**References**

1. Takahashi, N: **Rare pancreatic neoplasms and mimics of pancreatic cancer.** In, *Pancreatic Cancer*. Edited by Heiken JP. Cambridge: Cambridge University Press; 2009, 175-192.

2. Ichikawa T, Sou H, Araki T, et al. **Duct-penetrating sign at MRCP: usefulness for differentiating inflammatory pancreatic mass from pancreatic carcinoma.** *Radiology* 2001, **221:**107-116.

3. Turcotte S, Turkhey B, Barak S, et al. **von Hippel-Lindau disease-associated solid microcystic serous adenomas masquerading as pancreatic neuroendocrine neoplasms.** *Surgery* 2012, **152**:1106-1117.

4. Machado MC, Machado MA. **Solid serous adenoma of the pancreas: an uncommon but important entity.** *Eur J Surg Oncol* **2008**, 34:730-733.

5. Hayashi K, Fujimitsu R, Ida M, et al. **CT differentiation of solid serous cystadenoma vs endocrine tumor of the pancreas**. *Eur J Radiol* **2012**, 81:e203-208.

6. Kim HJ, Byun JH, Park SH, et al. **Focal Fatty replacement of the pancreas: usefulness of chemical shift MRI**. *Am J Roentgenol* **2007**, 188:429-432.

### O26 Staging and reporting pancreatic malignancies

#### Isaac R Francis, Mahmoud, M Al-Hawary, Ravi K Kaza

##### Department of Radiology, University of Michigan Health System, Ann Arbor, Michigan, USA

Accurate staging of many malignancies including pancreatic adenocarcinoma is essential for determining patient treatment. Only a minority (15-20 %) of patients with pancreatic carcinoma is resectable at the time of presentation and patients with a curative resection with margin negative resections have a better prognosis than those with residual macroscopic disease after resection. So it is crucial to separate these tumors into those who are resectable, borderline resectable and unresectable and metastatic groups. The commonly used staging systems in the United States are those put forward by the American Joint Committee on Cancer [AJCC] and the National Comprehensive Cancer Network [NCCN]. The AJCC system is based on the TNM system, with the T based on location and size and local extent, N, nodal and M, distant metastasis. Vascular contact/abutment is described as + or < or > 180^0^. However there are a few pertinent findings that are not included in these staging systems which are essential for surgical planning and determining resectability status. These include the presence of venous thrombus, tumour contact with common hepatic artery to the origins of the right and left hepatic arteries, tumour contact with first SMA branch and most proximal draining vein into the SMV, as well the presence of arterial variants.

Despite the preference of standardized structured or template reporting over conventional reporting by referring clinicians, its adoption into daily clinical practice has been slow in coming. The most commonly used type of conventional or free style reporting, tends to either “bury” the pertinent information needed for patient management in lengthy reports which include irrelevant incidental findings or fail to mention aspects of the findings that are crucial the treatment and management of the current condition for which the patient was imaged. Structured or template reports on the other hand can provide relevant information that is essential for patient management and this is especially true for patients with pancreatic carcinoma, given the variability in expertise and definition of disease extent amongst different readers.

In an effort to move the concept of structured reporting, a consensus statement was issued in 2014 by both the Society of Abdominal Radiology and the American Pancreatic Association, regarding a reporting template for pancreatic adenocarcinoma. This has been jointly published simultaneously in two major journals: the American Journal of Gastroenterology and Radiology.

Some initial studies have shown the value of standardized structured reporting in tumour staging and surgical planning, both to our referring clinicians as well as radiologists and radiology trainees. Brook and colleagues compared the results of structured versus nonstructured reporting of findings of MDCT (multidetector computed tomography) for the staging and subjective assessment of resectability for pancreatic cancer and showed that surgeons were confident regarding decisions regarding tumor resectability when structured reports were available than with nonstructured reports. We and others have now started to work on developing a similar working reporting template with the Society of Abdominal Radiology through disease-focussed panels (DFP’S) s for cystic pancreatic lesions.

**References:**

1. Kee D, Zalcberg JR. Radiology reporting templates in oncology: a time for change. J Med Imaging Radiat Oncol 2009; 53(6):511–513.
2. Schwartz LH, Panicek DM, Berk AR, Li Y, Hricak H. Improving communication of diagnostic radiology findings through structured reporting. Radiology 2011; 260(1):174–181.
3. Al-Hawary MM, Francis IR, Chari ST, Fishman EK, Hough DM, Lu DS, Macari M, Megibow AJ, Miller FH, Mortele KJ, Merchant NB, Minter RM, Tamm EP, Sahani DV, Simeone DM.
4. Pancreatic ductal adenocarcinoma radiology reporting template: consensus statement of the Society of Abdominal Radiology and the American Pancreatic Association. Radiology. 2014 Jan; 270(1):248-260 and Gastroenterology 2014
5. Brook OR, Brook A, Vollmer CA et al. Structures reporting of multiphasic CT for pancreatic cancer: Potential effect of staging and surgical planning. Radiology 2015:274:464-472

### O27 Intraductal papillary mucinous neoplasm

#### Giovanni Morana

##### Radiological Department, General Hospital Ca’ Foncello, Treviso Italy

Intraductal papillary mucinous neoplasms (IPMNs) are a group of exocrine mucin-producing tumours, diagnosed at a mean age of 60 years, with a male prevalence [1].

IPMN arises from the epithelium of the pancreatic ductal system and can display the full spectrum of histologic dysplasia, including hyperplasia, adenoma, borderline tumour, in situ or invasive carcinoma [2].

Three types of IPMNs have been described [1]: the main duct type; the branch-duct type and the mixed type, which meet the criteria for both MD-IPMN and BD-IPMN. There are significant differences in frequencies of malignancy in IPMNs according to the morphological types, higher for MD-type (mean 61.6 %) and lower for BD-type (25.5 %) [3].

A clear differentiation between IPMN lesions with different pathologic expression can be difficult, unless clear evidence of malignancy is present. However, some imaging findings can be suggestive of aggressive behavior of the IPMN [3].

High risk stigmata suggest the high possibility that the lesion is malignant, thus requiring surgical resection if Patient is fit: main duct diameter > 10 mm for MD-IPMN, the presence of solid enhancing nodules within the cyst in BD-IPMN, or obstructive jaundice in presence of a cystic lesion of the pancreatic head. Worrisome features suggest the possibility that the lesion could evolve in malignant, thus requiring further workup by EUS, to better risk-stratify the lesion, and a strict follow-up: cyst > 3 cm, thickened enhanced cyst walls, MPD size of 5-9 mm, non-enhancing mural nodules, abrupt change in the MPD caliber with distal pancreatic atrophy, and lymphadenopathy.

The management of IPMN is mainly based on the location (MD- and mixed type-IPMN versus BD-IPMN), the size of the largest cyst in BD-IPMN, the presence of “high risk stigmata” (main duct diameter > 10 mm for MD-IPMN, the presence of solid enhancing nodules within the cyst in BD-IPMN, or obstructive jaundice in presence of a cystic lesion of the pancreatic head) or “worrisome features” (cyst of > 3 cm, thickened enhanced cyst walls, MPD size of 5-9 mm, non-enhancing mural nodules, abrupt change in the MPD caliber with distal pancreatic atrophy, and lymphadenopathy). Moreover, risk stratification for age and fit for surgery must be considered.

Patients with BD-IPMN and cysts of >3 cm and no “worrisome features” can also be considered for EUS to verify the absence of thickened walls or mural nodules, particularly if the patient is elderly, while if patient is young and fit for surgery, surgery should be strongly considered. Patients with BD-IPMN and cysts of <3 cm and no “worrisome features” should be considered for observation according to size stratification [3].

MD-IPMN with MPD dilation of 5-9 mm can also be considered as a worrisome feature, with a recommendation of evaluation, but no immediate resection [3].

Imaging plays a pivotal role in the management of patients with IPMNs, with the different imaging techinques offering specific imaging features. However, MRI with MRCP is the leading imaging technique, either in the initial assessment and in the follow-up [4-7].

**References**

1. Manfredi R, Mehrabi S, Motton M, Graziani R, Ferrari M, Salvia R, et al. MR imaging and MR cholangiopancreatography of multifocal intraductal papillary mucinous neoplasms of the side branches: MR pattern and its evolution. Radiol Med 2008 Apr;113(3):414-428.

2. Ishida M, Egawa S, Aoki T, Sakata N, Mikami Y, Motoi F, et al. Characteristic clinicopathological features of the types of intraductal papillary-mucinous neoplasms of the pancreas. Pancreas 2007 Nov;35(4):348-352.

3. Tanaka M, Fernandez-del Castillo C, Adsay V, Chari S, Falconi M, Jang JY, et al. International consensus guidelines 2012 for the management of IPMN and MCN of the pancreas. Pancreatology 2012 May-Jun;12(3):183-197.

4. Sainani NI, Saokar A, Deshpande V, Fernandez-del Castillo C, Hahn P, Sahani DV. Comparative performance of MDCT and MRI with MR cholangiopancreatography in characterizing small pancreatic cysts. AJR Am J Roentgenol 2009 Sep;193(3):722-731.

5. Sahani DV, Kadavigere R, Blake M, Fernandez-Del Castillo C, Lauwers GY, Hahn PF. Intraductal papillary mucinous neoplasm of pancreas: multi-detector row CT with 2D curved reformations--correlation with MRCP. Radiology 2006 Feb;238(2):560-569.

6. Kim JH, Eun HW, Park HJ, Hong SS, Kim YJ. Diagnostic performance of MRI and EUS in the differentiation of benign from malignant pancreatic cyst and cyst communication with the main duct. Eur J Radiol 2012 Nov;81(11):2927-2935.

7. Lee HJ, Kim MJ, Choi JY, Hong HS, Kim KA. Relative accuracy of CT and MRI in the differentiation of benign from malignant pancreatic cystic lesions. Clin Radiol 2011 Apr;66(4):315-321.

### O28 Cystic pancreatic tumours

#### Mirko D’Onofrio (mirko.donofrio@univr.it)

##### Department of Radiology, University Hospital G.B. Rossi, Piazzale L.A. Scuro 10, 37134, University of Verona, Verona, Italy

Pancreatic neoplasms are a wide group of solid and cystic lesions with different and sometimes characteristic imaging features, clinical presentations and management.

Cystic pancreatic neoplasms comprise serous neoplasms, which are almost always benign, mucinous cystic neoplasms and intraductal papillary mucinous neoplasms, which can vary from benign to frankly malignant lesions, and solid pseudopapillary tumours.

Serous cystadenoma (SCA) is a cystic tumour with a typical multilocular “honeycomb” architecture due to the presence of multiple microcysts (<20 mm), thin walls and multiple septa oriented toward a central scar. The typical lobulated, “cloud-like” morphology is usually clearly depictable at imaging. The cystic content is anechoic at US, hypodense at CT and hypointense on T1-weighted images at MR; T2-weighted images clearly demonstrates the microcystic pattern.

After intravenous administration of contrast material, the hypervascularization of the central scar and of internal thin septa may be seen.

SCA does not communicate with the pancreatic ductal system and this can be well demonstrated at MRCP: this finding remains crucial for the differential diagnosis in respect to branch duct IPMNs.

SCA uncommon presentations are: macrocystic and unilocular; pseudosolid; huge dimensions.

Mucinous cystic neoplasms (MCNs) are pancreatic cystic tumors with different degress of malignancy. They show clear female sex predilection and usually appear as a single lesion with a rounded “ball-like” morphology, usually located in the body-tail of the pancreas probably due to its close position in respect to the rudimental ovary, and without communication with the pancreatic ductal system. Mucinous cystadenoma (MCA) usually presents as a macrocystic lesion, with irregular septa, thick walls and complex content that can be corpuscolated, viscous and dense mainly owing to mucinous content. This content makes very often the lesion heterogeneously hypoechoic at US, hypodense at CT and slightly hyperintense on T2-weighted images. On T1-weighted images, the signal intensity can vary from hypointensity, more common, to hyperintensity depending on mucin concentration. MRCP clearly demonstrates the lack of communication with the pancreatic ductal system. Differing from SCA, on post-contrast imaged, the intralesional septa are disorganised and peripherally located, describing a “bridge” along the cystic wall with a “pseudonodular” appearance. Peripheral calcifications along the thick wall can be detected, especially at CT .

MCA uncommon presentations are: uncommon site and gender; disepithelized.

The purpose of this paper is to present common and uncommon clinical and radiological presentations of cystic pancreatic tumors providing examples of multi-modality imaging approach with pathologic correlations, thus describing the histopathological bases on which it can be explain the peculiar imaging features, in order to avoid relevant misdiagnosis and to improve lesion diagnosis and management.

### O29 Diffusion-weighted imaging of head and neck tumours

#### Harriet C. Thoeny

##### Department of Radiology, Neuroradiology and Nuclear Medicine, Inselspital, University of Bern, Bern, Switzerland

Diffusion-weighted MR imaging (DW-MRI) is a challenging noninvasive MR technique that shows the Brownian motion in the extracellular extravascular space providing functional and microstructural information of the underlying tissue. In neuroradiology this technique is already an established method for the detection of an acute stroke, however, extracranial applications gained importance only in recent years. In head and neck radiology DW-MRI has several potential applications as an additional and comprehensive tool next to conventional MRI providing information on morphological alterations of the underlying tissue. It can be applied for the functional evaluation of the salivary glands, for the detection and characterisation of tumours, for lymph node staging, as well as for the differentiation between recurrent cancer and posttherapeutic changes after radiation therapy [1]. Furthermore, its potential for follow-up of patients undergoing radiation therapy of head and neck tumours might help to predict outcome at an early time point before morphological changes that occur usually relatively late in the time course of treatment.

Lower apparent diffusion coefficient (ADC) values are reported in most malignant compared to benign lesions in the head and neck region in adults and children [2].

For nodal staging DW-MRI has shown promise to be able to detect lymph node metastases even in subcentimetric nodes with lower ADC values compared to normal or reactive nodes. Early response to treatment follow up is reflected in an ADC increase in the primary tumour and nodal metastases; whereas non-responders tend to reveal only a slight increase or even a decrease in ADC during follow up [2].

However, optimization and standardisation of DWI technical parameters, comparison of DW images to morphological images and increasing experience are prerequisites for successful application of this challenging technique in the evaluation of various head and neck pathologies (2).

**References**

1) Thoeny HC. Diffusion-weighted MRI in Head and Neck Radiology: applications in oncology. Cancer Imaging 2011

2) Diffusion-weighted MR imaging in the head and neck. Thoeny HC, De Keyzer F, King AD. Radiology 2012

### O30 Radiation injury in the head and neck

#### Ann D King (king2015@cuhk.edu.hk)

##### Department of Imaging and Interventional Radiology, Chinese University of Hong Kong, Hong Kong, China

Complications of radiotherapy are important cause of morbidity and mortality in head and neck cancer survivors and may be exacerbated by the addition of chemotherapy. These complications may be symptomatic or found incidentally during imaging surveillance. It is important to be aware of these radiation induced complications as they may require treatment and also they should not be mistaken for recurrent tumour. The head and neck is a complex region and treatment induced complications involve many different structures. Examples of complications related to radiotherapy will be illustrated to show the broad range of abnormalities which can be found at the following sites-

1. Neurological tissues including the cranial nerves and temporal lobes (white matter injury, necrosis, cysts and brain abscess).
2. Osteoradionecrosis and osteomyelitis of the skull base, mandible and cervical spine.
3. Mucositis involving the pharynx and paranasal sinuses (including polyps and mucocoeles).
4. Vascular damage to the arteries (stenosis, pseudo aneurysm and carotid blow-out).
5. Glandular tissues (salivary and pituitary)
6. Radiation induced neoplasms (sarcoma and squamous cell carcinoma).

The lecture will be interactive and will discuss a wide range of radiotherapy related complications, including some that mimic tumour recurrence.

**References**

1. Hsin CH, Tseng HC, Lin HP, Chen TH. Post-irradiation otitis media, rhinosinusitis, and their interrelationship in nasopharyngeal carcinoma patients treated by IMRT. Eur Arch Otorhinolaryngol. 2016 Feb;273(2):471-7.

2. Alhilali L, Reynolds AR, Fakhran S. Osteoradionecrosis after radiation therapy for head and neck cancer: differentiation from recurrent disease with CT and PET/CT imaging. AJNR Am J Neuroradiol. 2014 Jul;35(7):1405-11.

3. Al-Saleh MA, Jaremko JL, Saltaji H, Wolfaardt J, Major PW. MRI findings of radiation-induced changes of masticatory muscles: a systematic review. J Otolaryngol Head Neck Surg. 2013 Mar 28;42:26.

4. Lobert P, Srinivasan A, Shah GV, Mukherji SK. Postoperative and postradiation changes on imaging. Otolaryngol Clin North Am. 2012 Dec;45(6):1405-22.

5. Shah R, Vattoth S, Jacob R, Manzil FF, O’Malley JP, Borghei P, Patel BN, Curé JK. Radiation necrosis in the brain: imaging features and differentiation from tumor recurrence. Radiographics. 2012 Sep-Oct;32(5):1343-59.

6. Xie CM, Liu XW, Li H, Zhang R, Mo YX, Li JP, Geng ZJ, Zheng L, Lv YC, Wu PH. Computed tomographic findings of skull base bony changes after radiotherapy for nasopharyngeal carcinoma: implications for local recurrence. J Otolaryngol Head Neck Surg. 2011 Aug;40(4):300-10.

7. King AD, Griffith JF, Abrigo JM, Leung SF, Yau FK, Tse GM, Ahuja AT. Osteoradionecrosis of the upper cervical spine: MR imaging following radiotherapy for nasopharyngeal carcinoma. Eur J Radiol. 2010 Mar;73(3):629-35.

8. Wang YX, King AD, Zhou H, Leung SF, Abrigo J, Chan YL, Hu CW, Yeung DK, Ahuja AT. Evolution of radiation-induced brain injury: MR imaging-based study. Radiology. 2010 Jan;254(1):210-8.

9. Abrigo JM, King AD, Leung SF, Vlantis AC, Wong JK, Tong MC, Tse GM, Ahuja AT. MRI of radiation-induced tumors of the head and neck in post-radiation nasopharyngeal carcinoma. Eur Radiol. 2009 May;19(5):1197-205.

10. King AD, Ahuja AT, Leung SF, Abrigo J, Wong JK, Poon WS, Woo KS, Chan HS, Tse GM. MR imaging of nonmalignant polyps and masses of the nasopharynx and sphenoid sinus after radiotherapy for nasopharyngeal carcinoma. AJNR Am J Neuroradiol. 2008 Jun;29(6):1209-14.

11. King AD, Ahuja AT, Yeung DK, Wong JK, Lee YY, Lam WW, Ho SS, Yu SC, Leung SF. Delayed complications of radiotherapy treatment for nasopharyngeal carcinoma: imaging findings. Clin Radiol. 2007 Mar;62(3):195-203.

12. Fang FM, Leung SW, Wang CJ, Su CY, Lui CC, Chen HC, Sun M, Lin TM. Computed tomography findings of bony regeneration after radiotherapy for nasopharyngeal carcinoma with skull base destruction: implications for local control. Int J Radiat Oncol Biol Phys. 1999 May 1;44(2):305-9.

### O31 PET/MR of paediatric brain tumours

#### Giovanni Morana^1^, Arnoldo Piccardo^2^, Maria Luisa Garrè^3^, Andrea Rossi^1^

##### ^1^Neuroradiology Unit, Istituto Giannina Gaslini, Genoa, GE, 16145, Italy; ^2^Nuclear Medicine Unit, Ospedali Galliera, Genoa, GE, 16128, Italy; ^3^Neuro-oncology Unit, Istituto Giannina Gaslini, Genoa, GE, 16145, Italy

###### **Correspondence:** Giovanni Morana (giovannimorana@gaslini.org) – Neuroradiology Unit, Istituto Giannina Gaslini, Genoa, GE, 16145, Italy

Modern neuroimaging techniques represent an essential tool in the evaluation of paediatric brain tumours, the second most common malignancy of childhood after leukaemia [1].

Conventional MRI is the imaging modality of choice for brain tumour identification and characterization but has limitations in distinguishing neoplastic from non-neoplastic lesions, defining tumour grade, guiding biopsy sampling, assessing treatment response, and predicting patient outcome [2]. Advanced MRI modalities, such as diffusion weighted imaging (DWI), perfusion weighted imaging (PWI) and magnetic resonance spectroscopy (MRS) have improved our understanding of brain tumours, overcoming some of the main limitations of conventional MRI. DWI gives information about the apparent diffusion coefficient of water and provides an opportunity to examine differences in cell density and tissue structure. PWI measures hemodynamic properties, such as tissue blood volume, and provides an indirect estimation of the degree of tumour angiogenesis. MRS estimates the levels of various metabolites within brain tissue that may be helpful for evaluating tumour aggressiveness [3]. Beyond MRI, metabolic imaging with PET provides further insights into tumour biological activity, especially when correlated with MRI; indeed, PET imaging combined with MRI, either by off-line co-registration or via hybrid PET/MR systems, is emerging as a valuable imaging modality not competing with but rather complementing MRI [4]; depending on the radiotracer used, different molecular processes can be assessed and a growing body of evidence supports the promising role of radiopharmaceuticals that target amino-acid transport, such as 11C-methionine (MET), 18F-dihydroxyphenylalanine (DOPA) and 18F-fluoroethyltyrosine (FET) [5,6]. Increased radiolabeled amino acid uptake in brain tumours correlates with increased use of amino acids for energy, protein synthesis, and cell division [4] and has proven advantageous over traditional 18F-fluorodeoxyglucose (FDG) because of the higher tumour to normal tissue uptake ratio [7]. Furthermore, as brain tumour uptake of amino acid tracers is predominantly determined by selective transport carried out by amino acid transporters, brain tumour depiction does not depend on the status of the blood-brain barrier, thus allowing amino acid uptake to occur in both enhancing and non-enhancing tumour components [8]. In the last few years fluorinated tracers such as 18F-DOPA and 18F-FET have emerged as alternative radiolabeled compounds to 11C-MET, given their longer half-life which allows a more widespread application even to centres without an on-site cyclotron.

Both tracers have been demonstrated to improve paediatric brain tumour management including tumour diagnosis, treatment planning, assessing response to treatment and post-treatment surveillance [6,9,10].

Integration of information obtained by MRI and PET should be performed by neuroradiologists and nuclear medicine physicians working in close collaboration, so as to properly appreciate and integrate the whole amount of diagnostic information and to offer clinicians more readily available information for treatment decision-making.

The most relevant applications of PET/MR imaging in paediatric brain tumours is the focus of the present work, with emphasis on diagnosis and surveillance of paediatric gliomas.

**References**

1. Brandão LA, Poussaint TY. Pediatric brain tumors. Neuroimaging Clin N Am. 2013,23:499-525.

2. Mabray MC, Barajas RF Jr, Cha S. Modern brain tumor imaging. Brain Tumor Res Treat. 2015,3:8-23.

3. Rossi A, Gandolfo C, Morana G, Severino M, Garrè ML, Cama A. New MR sequences (diffusion, perfusion, spectroscopy) in brain tumours. Pediatr Radiol. 2010,40:999-1009.

4. Heiss WD, Raab P, Lanfermann H. Multimodality assessment of brain tumors and tumor recurrence. J Nucl Med. 2011,52:1585-600.

5. Pirotte B, Acerbi F, Lubansu A, et al. PET imaging in the surgical management of pediatric brain tumors. Childs Nerv Syst. 2007;23:739-51

6. Dunkl V, Cleff C, Stoffels G, Judov N, Sarikaya-Seiwert S, Law I, et al. The usefulness of dynamic O-(2-18F-fluoroethyl)-L-tyrosine PET in the clinical evaluation of brain tumors in children and adolescents. J Nucl Med. 2015;56:88-92.

7. Utriainen M, Metsähonkala L, Salmi TT, et al. Metabolic characterization of childhood brain tumors: comparison of 18F-fluorodeoxyglucose and 11C-methionine positron emission tomography. Cancer. 2002;95:1376-86.

8. Morana G, Piccardo A, Garrè ML, Nozza P, Consales A, Rossi A. Multimodal MRI and 18F-DOPA PET in early characterization of pseudoresponse and nonenhancing tumor progression in a pediatric patient with malignant transformation of ganglioglioma treated with bevacizumab. J Clin Oncol. 2013,31:e1-5.

9. Morana G, Piccardo A, Puntoni M, Nozza P, Cama A, Raso A, et al. Diagnostic and prognostic value of 18F-DOPA PET and 1H-MR spectroscopy in pediatric supratentorial infiltrative gliomas: a comparative study. Neuro-oncology. 2015,17:1637-47.

10. Morana G, Piccardo A, Milanaccio C, Puntoni M, Nozza P, Cama A, et al. Value of 18F-3,4-Dihydroxyphenylalanine PET/MR Image Fusion in Pediatric Supratentorial Infiltrative Astrocytomas: A Prospective Pilot Study. J Nucl Med. 2014,55:718-23.

### O32 Structured reporting and beyond

#### Hebert Alberto Vargas

##### Department of Radiology, Memorial Sloan Kettering Cancer Center. New York, NY, USA

There are many key elements necessary to maximise the clinical utility of diagnostic imaging exams, including a pertinent clinical indication, adequate technical acquisition, accurate interpretation and effective communication of the imaging findings. The literature suggests that structured reporting in radiology leads to clearer and more thorough communication of relevant diagnostic findings than does conventional, free-form reporting. In a study of body oncologic CT examinations, structured reports were given significantly higher satisfaction ratings by both radiologists and referring physicians compared to “free-form” reports [1]. Barbosa et al. found that in addition to being preferred by the majority of the radiologists and endocrinologists participating in a study evaluating thyroid ultrasounds, the use of structured reporting resulted in improved standardisation of thyroid finding descriptors [2]. A study of coronary CT angiograms found an improved inter-observer agreement for the number of vessels with significant stenosis when a structured reporting software which required the radiologist to explicitly state which vessels were involved was used [3]. Other structured reporting software with features such as drop-down menus which facilitate data entry and minimize the amount of free-text entries have been shown to aid not only data comprehension but also reduce the length of time required for aortic aneurysm imaging [4].

However, the benefits of structured reporting cannot be accepted dogmatically. An accurate interpretation reported in “free-form” style is more clinically useful than a structured report containing erroneous information. Furthermore, the terminology used in structured reports also requires standardisation. Khorasani et al reported poor agreement between radiologists and non-radiologists in the interpretation of the most commonly used phrases in radiology reports [5]. In recent surveys gathering opinions about radiology reports, 20 % of the responding clinicians indicated that they found the language and style of radiology reports unclear [6]. Another study found that referring clinicians may reach different conclusions when reading the same reports [7].

Another important issue relevant to standardised reporting is the expression of diagnostic certainty. Radiologists are often tasked with summarising multiple findings and rendering an opinion with regards to potential explanations for the radiographic findings. There are scenarios in which no differential diagnoses are warranted and the findings are reported in terms of the absolute presence or absence of a pathologic process (e.g. “no fracture”). In other cases the findings are not definitive, and radiologists need to indicate their level of certainty for their interpretation of the imaging findings. In a study of patients with prostate cancer, 38 different terms were used in MRI reports to express the levels of certainty for the presence of extracapsular extension, prior to the introduction of a 5-point “certainty lexicon” [8]. The lexicon not only simplified the communication of the radiologists’ level of suspicion but also allowed more objective quantification of the diagnostic performance of MRI for diagnosing ECE, with a reported area under the curve of 0.85 [8]. The development of standardised “lexicons” to indicate the radiologists’ level of certainty for interpreting the imaging findings should therefore be considered an integral component of structured reports.

**References**

1. Schwartz LH, Panicek DM, Berk AR, Li Y, Hricak H. Improving communication of diagnostic radiology findings through structured reporting. Radiology 2011; 260:174-181

2. Barbosa F, Maciel LM, Vieira EM, Azevedo Marques PM, Elias J, Muglia VF. Radiological reports: a comparison between the transmission efficiency of information in free text and in structured reports. Clinics 2010; 65:15-21

3. Ghoshhajra BB, Lee AM, Ferencik M, et al. Interpreting the interpretations: the use of structured reporting improves referring clinicians’ comprehension of coronary CT angiography reports. Journal of the American College of Radiology : JACR 2013; 10:432-438

4. Karim S, Fegeler C, Boeckler D, L HS, Kauczor HU, von Tengg-Kobligk H. Development, implementation, and evaluation of a structured reporting web tool for abdominal aortic aneurysms. JMIR research protocols 2013; 2:e30

5. Khorasani R, Bates DW, Teeger S, Rothschild JM, Adams DF, Seltzer SE. Is terminology used effectively to convey diagnostic certainty in radiology reports? Academic radiology 2003; 10:685-688

6. Bosmans JM, Weyler JJ, De Schepper AM, Parizel PM. The radiology report as seen by radiologists and referring clinicians: results of the COVER and ROVER surveys. Radiology 2011; 259:184-195

7. Bastuji-Garin S, Schaeffer A, Wolkenstein P, et al. Pulmonary embolism; lung scanning interpretation: about words. Chest 1998; 114:1551-1555

8. Wibmer A, Vargas HA, Sosa R, Zheng J, Moskowitz C, Hricak H. Value of a Standardized Lexicon for Reporting Levels of Diagnostic Certainty in Prostate MRI. AJR Am J Roentgenol. 2014 Dec;203(6):W651-W657.

### O33 Massachusetts General Hospital experience with structured reporting

#### Theresa C. McLoud

##### Department of Radiology, Massachusetts General Hospital, Harvard Medical School, 55 Fruit Street, Boston, MA 02114, USA

A structured report is determined by a pre-defined report organisation based on templates that are used repetitively. Templates containing preferred common language describe findings and diagnoses. Structured reporting adds value because it limits inconsistency, avoids confusion and ambiguity. Preferred terms and definitions are used consistently which aids teaching, research, and clinical practice. Structured reports can contain tools for radiologists for appropriate content for any given indication or diagnosis.

Because radiologists may be reluctant to adopt structured reporting, at our institution we implemented certain tools which were helpful in assuring the success of such reporting. Group buy in and Involvement in the creation of report templates was used and each subspecialty division developed standardised normal reports. There were also group financial incentives for adherence to structured reporting. The MGH standard report includes patient information, date and time, requesting physician and history, and exam protocol. Templates are provided for the dictation of the standard normal report for any imaging study. Predetermined auto text can be added appropriately.

Our department has also developed clinical decision support templates for radiologists which integrate standards and best practices at the radiology point of care. The clinical decision support template helps radiologists make appropriate recommendations for further imaging work up based on best practices and guidelines. Examples include lung cancer screening, management of thyroid and adrenal nodules. Algorithms which included decision trees and end points for recommendations are included. A demonstration of the software for the clinical decision support tool for lung cancer screening will be demonstrated during the lecture.

**References:**

1. Lacson R, Prevedello LM, Andriole KP, et al: **Factors associated with radiologists’ adherence to Fleischner Society guidelines for management of pulmonary nodules.** *J Am Coll Radiol* 2012, 9(7):468-473.

2. Lu MT, Rosman DA, Wu CC, et al: **Radiologist point-of-care clinical decision support and adherence to guidelines for incidental lung nodules.** *J Am Coll Radiol* 2016, 13(2):156-162.

3. Larson DB, Towbin AJ, Pryor RM, et al: **Improving consistency in radiology reporting through the use of department-wide standardized structured reporting.** *Radiology* 2013, 267(1):1-7.

### O34 The oncologist’s perspective: what the oncologist needs to know

#### Nick Reed

##### Beatson oncology Centre, Glasgow, UK

It is a two way process with the oncologist needing to know information about the tumour but with the imaging specialist needing good information to allow the provision of an accurate and clinically useful report. It is now a given that cancer management takes part within the multidisciplinary process with tumour boards that meet regularly to discuss all new or difficult refractory cases. The use of agreed clinical protocols will help to streamline care by ensuring that patients get access to the most appropriate imaging technique and that it is carried out according to standardised and agreed clinical protocols. The imaging specialist is best placed to advise which modality is most appropriate and sometimes several modalities are required to get the information needed. The oncologist needs to know the diagnosis, staging and anatomy of the surrounding organs at risk; and if radiotherapy is being given how to target the tumour with modern state-of-the-art intensity in the modulated radiotherapy planning technologies. This is also essential to assess the response to treatment and no treatment is working but equally to know if treatment is not working so that treatment can be suspended or changed. For follow-up, imaging is part of the process of looking for evidence of recurrence. After radiation treatment it may be challenging to determine whether there is recurrence or post-radiation fibrosis and the imaging specialist plays a vital role in helping to determine this. Clinical trials play an important part of the oncologist workload and so there are particular issues in assessing response to treatment which have to comply with international guidelines and ruling from the regulatory bodies. Imaging specialists require time to get used to the RECIST criteria. Many of the new cancer drugs have brought with them their own fresh challenges as classical responses not necessarily seen. Tumours may stop growing but this is often equivalent to a response as these stabilisations may be quite durable. The role of functional imaging is becoming more important and changes in tumour blood flow dynamics may indicate at an early stage whether the tumour is responding. Both the imaging specialist and the oncologist have to learn how to deal with these changing circumstances and this is best done by team working and respecting each other’s competencies and skills. This talk will try to illustrate how the two disciplines can work together to provide the optimal care for the patient.

### O35 Towards the cure of all children with cancer: global initiatives in pediatric oncology

#### Carlos Rodriguez-Galindo

##### Department of Global Pediatric Medicine, St. Jude Children’s Research Hospital, Memphis, TN 38105, USA

Reduction of child mortality is one of the Millennium Development Goals; as low- and middle- income countries (LMIC) advance towards the achievement of this goal, initiatives aimed at reducing the burden of non-communicable diseases, including childhood cancer, need to be developed. Approximately 185,000 children are diagnosed with cancer every year worldwide; of those, 80 % live in LMIC, which account for 90 % of the deaths. Lack of quality population-based cancer registries in LMIC limits our knowledge of the epidemiology of pediatric cancer; however, available information showing variations in incidence may indicate unique interactions between environmental and genetic factors that could provide clues to etiology. Outcome of children with cancer in LMIC is dictated by late presentation and under diagnosis, high abandonment rates, high prevalence of malnutrition and other co-morbidities, suboptimal supportive and palliative care, and limited access to curative therapies. Initiatives integrating program building with education of health care providers and research have proven to be successful in the development of regional capacity. Intensity-graduated treatments adjusted to the local capacity have been developed. Regional collaborative initiatives have been developed in Central and South America and the Caribbean, Africa, the Middle East, Asia, and Oceania. These initiatives integrate regional capacity building, education of healthcare providers, implementation of intensity-graduated treatments, and establishment of research programs that are adjusted to the local capacity and needs. Together, the existing consortia and regional networks operating in LMIC have the potential to reach out to close to 60 % of all children with cancer worldwide. In summary, childhood cancer burden is shifted towards LMIC; global initiatives directed at paediatric cancer care and control are urgently needed. International partnerships facilitating step-wise processes that build capacity while incorporating epidemiology and health services research and implementing intensity-graduated treatments have shown to be effective. Regional networks aiming to build capacity while incorporating epidemiology, health-services, and outcomes research should be supported.

### O36 Multiparametric imaging of renal cancers

#### Hersh Chandarana

##### Department of Radiology, New York University School of Medicine, New York 10016, USA

Small renal masses are increasingly diagnosed incidentally. This results in management dilemma because numbers of small renal masses are either benign tumours such as angiomyolipoma (AML) or oncocytoma, or are neoplasms with indolent behavior [1]. Surgical treatments although provide excellent oncologic control is associated with development and worsening of renal insufficiency and associated cardiovascular morbidity [2]. Therefore, ability to non-invasively investigate renal tumor histopathology and aggressiveness can guide treatment decision and lower treatment cost.

Within this paradigm, the role of radiologist and imaging is evolving to predicting aggressiveness and biology of the tumor as well as providing operative guidance. MR imaging can play a very important role not only as a problem solving tool, but can provide deeper insight into tumour biology through techniques such as diffusion weighted imaging (DWI) and perfusion weighted imaging (PWI). Number of key observations highlighting the role of MR including advance imaging techniques in evaluation of renal masses is as listed below:

**1. Differentiating benign renal masses from malignant tumour.**

- There is some controversy regarding the role of signal loss on opposed phase chemical shift imaging in discriminating AML from RCC [3,4].

- Lipid poor AML tend to have uniform low T2 signal, uniform enhancement without evidence for necrosis, and restricted diffusion [5,6].

- There is overlap in the morphologic features of Oncocytoma and RCC on conventional imaging [7, 8]. Pilot data suggests that DWI and PWI may have a role in discriminating these benign renal tumours.

**2. Histologic subtyping RCC**

- Papillary subtype of RCC usually have low T2 signal, hypovascular when compared to clear cell RCC, and have lower Apparent diffusion coefficient (ADC) values. Furthermore, clear cell subtype have heterogeneous T2 signal and demonstrate heterogeneous hypervascularity [9].

- Chromophobe subtype is difficult to differentiate from clear cell RCC on the basis of enhancement. However, advance diffusion and perfusion MR techniques have shown some promise [10].

**3. Predicting tumor aggressiveness/outcome**

**-** Cystic RCC with less than 25 % solid enhancing component tend to be less aggressive than solid RCC [11].

**-** High stage clear cell RCC tend to me more heterogeneous with different texture compared to low stage RCC on Apparent diffusion coefficient (ADC) map [12].

**-** High grade clear cell RCC tend to have lower ADC compared to low grade clear cell RCC [13].

**References:**

1. Thompson RH, Kurta JM, Kaag M, et al. Tumor size is associated with malignant potential in renal cell carcinoma. J Urol 2009;181(5):2033–6.

2. Huang WC, Levey AS, Serio AM, et al. Chronic kidney disease after nephrectomy in patients with renal cortical tumours: A retrospective cohort study. *The Lancet. Oncology*. 2006;7:735-740

3. Kim JK, Kim SH, Jang YJ, et al. Renal angiomyolipoma with minimal fat: differentiation from other neoplasms at double-echo chemical shift FLASH MR imaging. Radiology. 2006 Apr;239(1):174-80.

4. Jhaveri KS, Elmi A, Hosseini-Nik H, et al. Predictive Value of Chemical-Shift MRI in Distinguishing Clear Cell Renal Cell Carcinoma From Non-Clear Cell Renal Cell Carcinoma and Minimal-Fat Angiomyolipoma.

5. Hindman N, Ngo L, Genega EM, et al. Angiomyolipoma with minimal fat: can it be differentiated from clear cell renal cell carcinoma by using standard MR techniques? Radiology. 2012 Nov;265(2):468-77.

6. Sasiwimonphan K, Takahashi N, Leibovich BC, Carter RE, Atwell TD, Kawashima A. Small (<4 cm) renal mass: differentiation of angiomyolipoma without visible fat from renal cell carcinoma utilizing MR imaging. Radiology. 2012 Apr;263(1):160-8.

7. Rosenkrantz AB, Hindman N, Fitzgerald EF, Niver BE, Melamed J, Babb JS. MRI features of renal oncocytoma and chromophobe renal cell carcinoma. AJR Am J Roentgenol. 2010 Dec;195(6):W421-7.

8. Schieda N, Al-Subhi M, Flood TA, El-Khodary M, McInnes MD. Diagnostic accuracy of segmental enhancement inversion for the diagnosis of renal oncocytoma using biphasic computed tomography (CT) and multiphase contrast-enhanced magnetic resonance imaging (MRI). Eur Radiol. 2014 Nov;24(11):2787-94.

9. Chandarana H, Rosenkrantz AB, Mussi TC, et al. Histogram analysis of whole-lesion enhancement in differentiating clear cell from papillary subtype of renal cell cancer. Radiology. 2012 Dec;265(3):790-8.

10. Chandarana H, Amarosa A, Huang WC, et al. High temporal resolution 3D gadolinium-enhanced dynamic MR imaging of renal tumors with pharmacokinetic modeling: preliminary observations. J Magn Reson Imaging. 2013 Oct;38(4):802-8.

11. Doshi AM, Huang WC, Donin NM, Chandarana H. MRI features of renal cell carcinoma that predict favorable clinicopathologic outcomes. AJR Am J Roentgenol. 2015 Apr;204(4):798-803.

12. Kierans AS, Rusinek H, Lee A, et al. Textural differences in apparent diffusion coefficient between low- and high-stage clear cell renal cell carcinoma. AJR Am J Roentgenol. 2014 Dec;203(6):W637-44.

13. Rosenkrantz AB, Niver BE, Fitzgerald EF, Babb JS, Chandarana H, Melamed J. Utility of the apparent diffusion coefficient for distinguishing clear cell renal cell carcinoma of low and high nuclear grade. AJR Am J Roentgenol. 2010 Nov;195(5):W344-51.

### O37 Linking imaging features of renal disease and their impact on management strategies

#### Hebert Alberto Vargas

##### Department of Radiology, Memorial Sloan Kettering Cancer Center. New York, NY, USA

Most renal tumours are currently detected incidentally on imaging exams performed for a non-urologic indication. At the same time, the contemporary approach to the management of renal masses has also evolved, with additional options to the classic nephrectomy approach including nephron sparing surgery, focal therapies such as cryoablation and the growing use of active surveillance for masses considered unlikely to ultimately result in significant morbidity or mortality. The role of imaging is also shifting from mere tumor detection to providing crucial information required to tailor the management strategy to individual patients. In this session, we will discuss the key imaging findings that need to be evaluated in patients with renal tumours, and how they can be used to triage patients and guide treatments.

### O38 Adrenals, retroperitoneum and peritoneum

#### Isaac R Francis, Ashish P Wasnik

##### Department of Radiology, University of Michigan Health System, Ann Arbor, Michigan, USA

**Adrenal:**

Atypical adenomas:

Atypical large adrenal adenomas can rarely undergo degeneration and necrosis, and cannot be distinguished from malignant tumors and are frequently resected. It is possible that these tumours are not FDG avid and hence could be separated from malignancies but again they have not been studied in large enough numbers with PET-FDG to determine this.

Fat-containing masses:

These include most often benign lesions but rarely primary malignant adrenal tumours as well. Most adrenal masses containing large amounts of macroscopic fat are adrenal myelolipomas. But there are other uncommon adrenal masses that can contain small amounts of macroscopic fat, such as adenomas with focal lipomatous hyperplasia and rarely adrenal cortical carcinoma and pheochromocytoma.

Primary and metastatic lesions showing rapid washout:

There have been reports of pheochromocytomas demonstrating rapid washout thereby mimicking adrenal adenomas. In at least two studies of pheochromocytomas, their incidence is about 15-20 %. In most instances these tumours are smaller in size (<5 cms) and are homogenous and usually biochemically active.

Metastases HCC and clear cell RCC, may demonstrate rapid washout thereby mimicking adrenal adenomas. If there is no prior imaging, this diagnosis could be problematic and a PET-CT or biopsy would be needed to determine the nature of the lesion. But if there is prior imaging, and the adrenal lesion is new or enlarging, it is likely to be a metastasis.

Primary and metastatic lesions showing signal loss on chemical shift (CSI) MRI:

Adrenal cortical carcinomas and pheochromocytomas may rarely contain enough intracellular lipid to show a quantitative loss of signal intensity [SI]. Metastases from HCC and clear cell carcinoma may contain enough intracellular lipid to cause of loss of SI on Chemical SI MR.

**Peritoneum/mesentery:**

Tuberculous peritonitis:

Peritoneal disease is the most common of extra-pulmonary tuberculosis and is frequently associated with other forms of tuberculosis. The imaging findings include nodular peritoneal thickening and ascites. These findings may be associated with enlarged lymph nodes and areas of GI tract involvement with the ileocecal junction being a common site.

Sclerosing peritonitis:

Also called sclerosing “encapsulating” peritonitis, this is a rare condition seen most often in patients who are undergoing have undergone peritoneal dialysis. When it encapsulates abdominal organs such as the small bowel, it is referred to as “abdominal cocoon”.

Gliomatosis peritonei:

This is a rare complication of a rupture of ovarian teratomas but can also be seen in patients with VP shunts. On imaging, these appear as peritoneal nodules, omental caking and ascites.

Splenosis:

Usually seen after trauma or surgery and is due to heterotopic autotransplantation of splenic tissue. The peritoneal nodules/masses show similar enhancement features similar to that of splenic tissue.

Sclerosing mesenteritis/mesenteric panniculitis/mesenteric lipodystrophy

Most often the etiology is idiopathic; there is more recently an association found in some patients with IgG4 disorders. It has a wide range of appearances on imaging: ranging from a proliferation of fat to calcified soft tissue mesenteric masses.

Mesenteric Omental necrosis/fat necrosis:

In most instances, seen as fatty mass which may encapsulated with adjacent soft tissue fat stranding. If the cause is omental torsion, swirling vessels are seen in the adjacent vasculature. Encapsulated fat necrosis can mimic a mass.

Fat saponification in patients with sequelae of acute pancreatitis can lead to fat saponification and to small peritoneal nodules of fat necrosis.

**References:**

1. Johnson PT, Horton KM, Fishman EK. Adrenal mass imaging with multidetector CT: Pathologic conditions, pearls and pitfalls. Radiographics 2009; 29:1333-1351

2. Choi YA, Kim CK, Park BK et al. Evaluation of adrenal metastases from renal cell carcinoma and hepatocellular carcinoma. Radiology 2013; 266:514-520

3. Patel J, Davenport MS, Cohan RC et al. Can Established CT Attenuation and Washout Criteria for Adrenal Adenoma Accurately Exclude Pheochromocytoma? AJR 2013; 201:122-127

4. Levy AD, Shaw JC, Sobin LH. From the Archives of the AFIP Secondary Tumors and Tumorlike Lesions of the Peritoneal Cavity: Imaging Features with Pathologic Correlation. Radiology 2009; 29:347-373

5. Epstein BM, Mann JH. CT of abdominal tuberculosis. AJR 1982; 139:861-866

6. George C, Al-Zwae K, Nair S et al. Computed tomography appearances of sclerosing encapsulated peritonitis. Clin Radiol 2007; 62:732-737

7. Okamoto D, Ishigami K, Yoshimitsu K et al. Gliomatosis peritonei associated with immature teratoma: a mimicker of peritoneal dissemination of malignant diseases. J Comput Assist Tomogr 2007; 31:317-319

8. Mendelson DS, Cohen BA, Armas RR. CT appearance of splenosis. J Comput Assist Tomogr 1982; 6:1188-1190

9. Kamaya A, Federle MP, Desser TS. Imaging Manifestations of Abdominal Fat Necrosis and Its Mimics. Radiographics 2011; 31:2021-2034

10. Takao H, Yamahira K, Watanabe T. Encapsulated fat necrosis mimicking abdominal liposarcoma: computed tomography findings. J Comput Assist Tomogr 2004; 28:193–194

### O39 Lung and pleura

#### Stefan Diederich (Stefan.diederich@vkkd-kliniken.de)

##### Department of Diagnostic and Interventional Radiology, Marien Hospital, 40479 Düsseldorf, Germany

**Lung:** Pulmonary nodules are commonly observed in patients with cancer as well as in patients with no known malignancy particularly in heavy smokers. Most of these nodules are small (less than 8 mm). Even in cancer patients a large proportion of these small nodules are benign [1]. The likelihood of malignancy depends on individual aspects (cancer type, grading, staging, molecular markers etc.), nodule size and risk factors [1]. For example, in a heavy smoker with lung cancer and one additional nodule larger than 8 mm the nodule is more likely to represent a second primary than a solitary metastasis. In a non-smoker with advanced high-grade soft-tissue sarcoma a solitary nodule is more likely to represent a metastasis. In all cancer patients a significant proportion of pulmonary nodules represent benign lesions such as pulmonary lymph nodes or granulomas [2, 3].

Non-solid nodules (ground glass opacities) are more likely to represent lung cancer (adenocarcinoma) than solid nodules [4, 5]. However, they may represent benign lesions such as focal fibrosis or haemorrhage. Furthermore, some (haemorrhagic) metastases may present as non-solid nodules [6].

Consolidation or diffuse ground glass usually represents benign disease such as pneumonia. However, in adenocarcinoma with predominantly lepidic growth consolidation or diffuse ground glass may be due to cancer spread in the lung.

**Pleura:** Pleural effusion may be due to malignant spread (pleural carcinomatosis) or to several benign conditions (heart or renal failure, haemorrhage, infection, etc.). Malignancy is usually confirmed by cytologic analysis of pleura fluid. However, imaging may suggest malignancy if solid pleural lesions are demonstrated within the effusion particularly at the lung base. Unilateral effusion, particularly in the left hemithorax or on the side of the underlying malignancy (e.g. breast, lung cancer) also suggests malignant effusion.

Solid pleural lesions may be clearly benign such as pleural lipoma or calcified pleural plaques in patients with asbestos exposure. Non-calcified focal solid lesions may be benign (e.g. following infection, haemorrhage) or malignant. Although pleural carcinomatosis is usually associated with effusion solid metastases without effusion can occur. Diagnosis usually requires histology [7].

**References**

1. Mery MT, Pappas AN, Bueno R, Mentzer SJ, Lukanich JM, Sugarbaker DJ, Jaklitsch MT: **Relationship between a history of antecedent cancer and the probability of malignancy for a solitary pulmonary nodule.** *Chest 2004*, **125**: 2175-2181.

2. Gould MK, Fletcher J, Iannettoni MD, Lynch WR, Midthun DE, Naidich DP, Ost DE; American College of Chest Physicians: **Evaluation of patients with pulmonary nodules: when is it lung cancer ?** ACCP evidence-based clinical practice guidelines (2^nd^ edition). Chest. 2007, 132:108S-130S.

3. Truong MT, Ko JP, Rossi SE, Rossi I, Viswanathan C, Bruzzi JF, Marom EM, Eramus JJ: **Update in the evaluation of the solitary pulmonary nodule.** Radiographics 2014, 34: 1658-1679

4. Naidich DP, Bankier AA, MacMahon H, Schaefer-Prokop CM, Pistolesi M, Goo JM, Macchiarini P, Crapo JD, Herold CJ, Austin JH, Travis WD: **Recommendations for the management of subsolid pulmonary nodules detected at CT: a statement from the Fleischner Society**. Radiology 2013, 266: 304-317.

5. Lee HY, Choi YL, Lee KS, Han J, Zo JI, Shim YM, Moon JW: **Pure ground-glass opacity neoplastic lung nodules: histopathology, imaging, and management.** AJR Am J Roentgenol 2014, 202: 224-233

6. Seo JB, Im JG, Goo JM, Chung MJ, Kim MY: **Atypical pulmonary metastases: spectrum of radiologic findings**. Radiographics 2001, 21: 403-417

7. Müller NL: **Imaging of the pleura**. Radiology 1993. 186: 297-309

### O40 Advances in MRI

#### Jurgen J. Fütterer

##### Department of Radiology and Nuclear Medicine Radboud University Medical Center, Nijmegen, 6500HB, The Netherlands

Prostate cancer is the most common non-cuteaneous neoplasm in men and the third leading cause of cancer-related death in men in Europe.

Lymph node metastases have a significant impact on the prognosis of patients with malignancies. Because benign and malignant lymph nodes have similar signal intensities in conventional MR imaging (MRI), metastatic lymph nodes are mainly identified by node enlargement. This may result in missing small metastases in normal sized nodes, resulting in false-negative metastasis. Routine cross-sectional imaging modalities, such as CT and conventional non-enhanced MR imaging, have a limited sensitivity in identifying lymph node metastases.

One of the methods to improve upon this sensitivity is the use of lymph node specific contrast agents, such as Ferumoxtran-10: an ultrasmall superparamagnetic iron oxide (USPIO) particle that has proven to be a valuable contrast agent for detecting lymph node metastases using MRI in various types of cancer (also called nano-MRI).

After intravenous injection, USPIO particles mitigate into the interstitial space, where they are incorporated in macrophages and transported to the lymph nodes. Because of the paramagnetic properties of the iron particles contained in ferumoxtran-10, healthy lymph nodes will feature reduced signal intensity in a T2* weighted MRI sequence and will appear dark in contrast. However, malignant parts of the lymph nodes will not contain any macrophages and therefore no iron. These lymph nodes remain high in signal intensity. Malignant lymph nodes can therefore be detected by their high signal intensity in strongly T2* weighted sequences [1]. A meta-analysis [2] showed a mean sensitivity of 0.90 and a specificity of 0.96 of the USPIO particles for malignant lymph node detection.

Small metastases can be prospectively recognized in small (3-10 mm) size lymph nodes using ferumoxtran-10-enhanced MR imaging [3]. These small lymph nodes would be considered to be benign in conventional MR or CT examinations based on size criteria. In addition, hyperplastic enlarged nodes can be correctly recognized as non metastatic, based on maintained uptake of ferumoxtran-10. In 41 % of patients with prostate cancer, nodal metastases outside the area of routine pelvic lymph node dissection are detected by using nano-MRI [4]. Furthermore, metastasis-directed therapies with salvage radiotherapy or salvage pelvic lymph node dissection has shown promising results in terms of biochemical recurrence-free survival and androgen therapy-free survival [5,6].

**References:**

1. Barentsz JO, Futterer JJ, Takahashi S: **Use of ultrasmall superparamagnetic iron oxide in lymph node MR imaging in prostate cancer patients**. *Eur J Radiol* 2007, **63(3)**:369-372.

2. Wu, L., et al.: **Diagnostic performance of USPIO-enhanced MRI for lymph-node metastases in different body regions: a meta-analysis**. *Eur J Radiol* 2011. **80(2)**: 582-589.

3. Harisinghani MG, Barentsz JO, Hahn, P, et al. **Noninvasive detection of clinically occult lymph-node metastases in prostate cancer**. *NEJM* 2003, **348**:2491-2499.

4. Heesakkers RA, Jager GJ, Hövels AM, et al.: **Prostate cancer: detection of lymph node metastases outside the routine surgical area with ferumoxtran-10-enhanced MR imaging**. *Radiology* 2009, **251(2)**:408-414.

5. Punnen S, Cooperberg MR, D’Amico AV, et al.: **Management of biochemical recurrence after primary treatment of prostate cancer: a systematic review of the literature**. *Eur Urol* 2013, **64(6)**:905–915.

6. Freedland SJ, Presti Jr JC, Amling CL, et al.: **Time trends in biochemical recurrence after radical prostatectomy: results of the SEARCH database.** *Urology* 2003, **61(4)**:736–741.

### O41 Advances in molecular imaging

#### Wim J.G. Oyen

##### The Institute of Cancer Research and The Royal Marsden NHS Foundation Trust, London, U.K

**Background:** Accurate staging and restaging of patients with prostate cancer is of pivotal importance, especially when considering loco-regional treatment of oligometastatic disease with a curative intent. Next to cross-sectional and functional imaging techniques using CT and MRI, the impact of molecular imaging has significantly increased in the last years due to a number of newly developed radiopharmaceuticals.

**Radiopharmaceuticals:** Now almost 20 years after the initial report on the utility of C-11 labelled *choline*, a key precursor in the biosynthesis of phosphatidylcholine (a major component of the cell membrane), C-11 and F-18 labelled choline-derivatives are available in many centres. Although the utility of choline PET/CT is well-established, the limited sensitivity in low volume disease (reflected by low PSA levels lower than 1 ng/mL) is a drawback when assessing patients suspected of oligometastatic disease.

Prostate-Specific Membrane Antigen (PSMA), an enzyme that in humans is encoded by the FOLH1 (folate hydrolase 1) gene, is considered an important target for molecular imaging as PSMA is a highly upregulated gene product in many prostate cancers. High expression of PSMA is associated with a shorter time to progression and a higher risk for patients to relapse. Initial immunoSPECT experience with In-111 labelled *Capromab Pendetide*, a murine, whole IgG monoclonal antibody targeting the intracellular domain of the PSMA molecule, was and quite challenging. The development of small PSMA-targeting molecules labelled with Ga-68 (*PSMA*)or F-18 (*DCFBC*, *DCFPyL*) for PET/CT imaging was a major step forward. Radiolabelling of the molecules is straightforward, while fast background clearance and high targeting allow early imaging after injection of the agents. High diagnostic yields have been reported in the lower PSA range, especially in patients with high PSA doubling time (>85 % when PSA < 1.0 ng/ml with PDT < 6 months).

An alternative for PSMA-based imaging agents for PET/CT is the synthetic L-leucine analogue fluciclovine (*FACBC*), which was very recently approved by the FDA. It depicts functional activity of two different transmembrane amino acid transporters (ASCT2 and LAT1). The distribution of the tracer in the body is favourable (negligible uptake in the kidneys, no activity in the urinary tract) and the detection rate of tumours seems to compare favourably to choline PET/CT when PSA levels are low. Yet another option is F-18 labelled 5α-dihydrotestosterone (*FDHT*), directly targeting the intracellular androgen receptor.

**Current status and future prospects:** In some countries in Europe, PSMA PET/CT has rapidly gained widespread acceptance for imaging patients with prostate cancer. Although an overwhelming number of favourable reports have been published recently, the definitive positioning in relation to the impact on patient management and outcome remains to be established. With the approval of FACBC, molecular imaging will become an available option for prostate cancer patients. Defining the position of all the available options for molecular imaging in the diagnostic algorithms of the various stages of disease and convert this into guidelines are upcoming challenges.

**References**

1. Fanti S, Minozzi S, Castellucci P, Balduzzi S, Herrmann K, Krause BJ, Oyen W, Chiti A. PET/CT with (11)C-choline for evaluation of prostate cancer patients with biochemical recurrence: meta-analysis and critical review of available data. Eur J Nucl Med Mol Imaging. 2016 Jan;43(1):55-69.

2. Larson SM, Morris M, Gunther I, Beattie B, Humm JL, Akhurst TA, Finn RD, Erdi Y, Pentlow K, Dyke J, Squire O, Bornmann W, McCarthy T, Welch M, Scher H. Tumor localization of 16beta-18F-fluoro-5alpha-dihydrotestosterone versus 18F-FDG in patients with progressive, metastatic prostate cancer. J Nucl Med. 2004 Mar;45(3):366-73.

3. Nanni C, Zanoni L, Pultrone C, Schiavina R, Brunocilla E, Lodi F, Malizia C, Ferrari M, Rigatti P, Fonti C, Martorana G, Fanti S. (18)F-FACBC (anti1-amino-3-(18)F-fluorocyclobutane-1-carboxylic acid) versus (11)C-choline PET/CT in prostate cancer relapse: results of a prospective trial. Eur J Nucl Med Mol Imaging. 2016 Aug;43(9):1601-10.

4. Perera M, Papa N, Christidis D, Wetherell D, Hofman MS, Murphy DG, Bolton D, Lawrentschuk N. Sensitivity, Specificity, and Predictors of Positive 68Ga-Prostate-specific Membrane Antigen Positron Emission Tomography in Advanced Prostate Cancer: A Systematic Review and Meta-analysis. Eur Urol. 2016 [Epub ahead of print].

5. Rowe SP, Macura KJ, Ciarallo A, Mena E, Blackford A, Nadal R, Antonarakis ES, Eisenberger MA, Carducci MA, Ross AE, Kantoff PW, Holt DP, Dannals RF, Mease RC, Pomper MG, Cho SY. Comparison of Prostate-Specific Membrane Antigen-Based 18F-DCFBC PET/CT to Conventional Imaging Modalities for Detection of Hormone-Naïve and Castration-Resistant Metastatic Prostate Cancer. J Nucl Med. 2016 Jan;57(1):46-53.

6. Rowe SP, Macura KJ, Mena E, Blackford AL, Nadal R, Antonarakis ES, Eisenberger M, Carducci M, Fan H, Dannals RF, Chen Y, Mease RC, Szabo Z, Pomper MG, Cho SY. PSMA-Based [(18)F]DCFPyL PET/CT Is Superior to Conventional Imaging for Lesion Detection in Patients with Metastatic Prostate Cancer. Mol Imaging Biol. 2016 Jun;18(3):411-9.

### O42 Incorporating advanced imaging, impact on treatment selection and patient outcome

#### Cheng Lee Chaw, Nicholas van As

##### Department of Clinical Oncology, Royal Marsden Hospital NHS Foundation Trust and the Institute of Cancer Research, London, UK

###### **Correspondence:** Nicholas van As (nicholas.vanAs@rmh.nhs.uk) – Department of Clinical Oncology, Royal Marsden Hospital NHS Foundation Trust and the Institute of Cancer Research, London, UK

Prostate cancer is a heterogenous disease where treatment is moving towards personalised therapy. In addition to histological confirmation and grading, imaging plays an important role in non-invasive detection, localization and staging of prostate cancer. Conventional staging such as computed tomography (CT) or magnetic resonance imaging (MRI) and whole body bone scintigraphy (BS) are the standard of practice at primary diagnosis or relapse. However, these modalities provide only morphological information but do not differentiate the biological nature and metabolic status of the tumours, therefore limiting its diagnostic performance.

New imaging techniques enable assessment on both structural change as well as the underlying pathophysiology of the tumour, thereby optimising and individualised treatment planning for patients with cancer. With the emerging data showing higher diagnostic performance of the new PET tracers ^18^F-Fluorocholine (^18^F-FCH) PET-CT and ^68^Galium (Ga)-PSMA PET-CT, additional options for prostate cancer staging have become available [1-3]. Other functional modalities including diffusion-weighted sequence MRI with superparamagnetic iron oxide and whole body diffusion MRI, have also been found useful in prostate cancer staging [4-5]. In addition to additional staging information provided, another advantage of these new imaging techniques over conventional staging is the ability to detect metastases at an earlier stage where disease is smaller volume and limited in number, namely oligometastases, thereby allowing for potentially radical treatments to be offered whether at primary or salvage setting.

Each of this imaging modality has particular advantages in detecting site-specific disease. For nodal disease, both (^68^GA)-PSMA and MRI with superparamagnetic iron oxide are highly sensitive in detecting micrometastases (<2 mm) within normal size lymph node, with the former able to achieve detection rate of 60 % at PSA < 0.5 ng/ml [5-6]. For bony disease assessment, whole body diffusion MRI excels in its detectability compared to conventional staging [7]. It is also useful in assessing response to treatment, therefore prompting change in treatment strategy for non-responders [7]. Although (^18^ F-FCH) PET-CT has lower sensitivity than ^68^ Ga –PSMA in detection of metastatic lesions, it was the one of the earliest functional imaging techniques adopted in prostate cancer management and has facilitated the application of metastatic-directed therapy in patients with oligometastases in the last 5 years, exploring a new treatment paradigm in the setting of metastatic disease [1]. Apart from diagnostic purposes, integrating the molecular level information obtained from these functional imaging, particularly PET-CT may potentially lead a pathway for therapy targeting.

The use of functional imaging in prostate cancer from staging, detection of relapse to treatment response monitoring and potential therapeutic targets is promising, rapidly evolving, and likely to deliver improved outcomes for patients.

**References:**

1. Evangelista L et al **New clinical indications for** 18F**/11C-Cholin, New tracers for Positron Emission Tomography and a promising hybrid device for prostate Cancer staging:** A Systematic Review of the literature. Eur Urol. pii s0302-2838(16)00131-7. Doi:10.10/j eruro.2016.01.029

2. Umbehr MH, Müntener M et al. **The role of 11C-choline and** 18F**-fluorocholine positron emission tomography (PET) and PET/CT in prostate cancer: a systematic review and meta-analysis. Eur. Urol**. (2013);64, 106–117.

3. Fanti S, Minozzi S et al, **PET/CT with (11)C-choline for evaluation of prostate cancer patients with biochemical recurrence: meta-analysis and critical review of available data**. Eur J Nucl Med Mol Imaging (2016); 43(1):55-69. doi: 10.1007/s00259-015-3202-7.

4. Larbi A, Dalluadiere B et al. **Whole Body MRI (WB-MRI) Assessment of metastatic spread in Prostate Cancer: Therapeutic Perspectives on Targeted Management of Oligometastastic Disease**; Prostate (2016): 1-10

5. Afshar-Oromieh A, Haberkorn U et al; **Comparison of PET imaging with a (68)Ga-labelled PSMA ligand and (18)F-choline-based PET/CT for the diagnosis of recurrent prostate cancer** Eur J Nucl Med Mol Imaging (2014); 41(1):11–20.

6. Thoeny HC, Froehlich JM et al. **Metastases in normal-sized pelvic lymph nodes: detection with diffusion-weighted MR imaging**. Radiology (2014) 273, 125-135.

7. Padhani AR et al; **Therapy monitoring of skeletal metastases with whole-body diffusion MRI;** JMRI (2014) 39:1049-1078

## Speaker presentations

### S1 Combining ADC-histogram features improves performance of MR diffusion-weighted imaging for lymph node characterisation in cervical cancer

#### Igor Vieira^1^, Frederik De Keyzer^2^, Elleke Dresen^2^, Sileny Han^3^, Ignace Vergote^3^, Philippe Moerman^4^, Frederic Amant^3,5^, Michel Koole^1^, Vincent Vandecaveye^2^

##### ^1^Department of Nuclear Medicine, University Hospitals Leuven, Belgium; ^2^Department of Radiology, University Hospitals Leuven, Belgium; ^3^Department of Obstretics and Gynaecology, University Hospitals Leuven, Belgium; ^4^Department of Pathology, University Hospitals Leuven, Belgium; ^5^Center Gynaecological Oncology Amsterdam (CGOA), Antoni van Leeuwenhoek; Amsterdam, The Netherlands

###### **Correspondence:** Igor Vieira (ifv.kul@gmail.com) – Department of Nuclear Medicine, University Hospitals Leuven, Belgium

**Aim**: To evaluate whether a statistical classification combining ADC-histogram features (apparent diffusion coefficient) (ADC) improves lymph node characterisation in patients with cervical cancer.

**Methods**: Thirteen patients with cervical cancer underwent pelvic magnetic resonance imaging (MRI) including diffusion-weighted imaging (DWI) with 6 b-values ranging between 0 and 1000 s/mm2. All pelvic lymph nodes of 4 mm or greater in short axis diameter were delineated and twelve first order statistics features were extracted from the ADC histogram. Three features were based on the cumulative ADC intensity-volume histogram: two describing the volume and intensity above a certain threshold of ADC max, and one based on area under the curve. A recursive feature selection algorithm was used to obtain the best combination of features using histopathology after surgical sampling as ground truth. A predictive model based on the optimal combination of features using logistic regression (LR) as classifier was compared with conventional ADC in terms of sensitivity, specificity and accuracy. This evaluation was done by nested cross validation for model selection.

**Results**: Radiological-histopathological correlation was possible for 94 lymph nodes (26 positive and 68 negative nodes). The predictive model combining two features: F6 (Skewness) and F3 (Median) resulted in 86.00 % ± 2.54 % sensitivity, 89.47 % ± 1.47 % specificity, and 88.51 % ± 1.77 % accuracy. In comparison, conventional ADC mean showed 72.56 % ± 3.19 % sensitivity, 73.68 % ± 1.38 % specificity and 73.37 % ± 1.88 % accuracy.

**Conclusion**: Classification combining two first order features extracted from the ADC-histogram of DWI MRI data improves characterization of lymph nodes in cervical cancer compared to conventional ADC.

### S2 Whole-body diffusion-weighted MRI for surgical planning in patients with colorectal cancer and peritoneal metastases

#### R Dresen^1^, S De Vuysere^1^, F De Keyzer^1^, E Van Cutsem^2^, A D’Hoore^3^, A Wolthuis^3^, V Vandecaveye^1^

##### ^1^Department of Radiology, Leuven Cancer Institute, University Hospitals Leuven, KU Leuven, Leuven, Belgium; ^2^Department of Oncology, Leuven Cancer Institute, University Hospitals Leuven, KU Leuven, Leuven, Belgium; ^3^Department of Abdominal Surgery, Leuven Cancer Institute, University Hospitals Leuven, KU Leuven, Leuven, Belgium

###### **Correspondence:** R Dresen (elleke.dresen@uzleuven.be) – Department of Radiology, Leuven Cancer Institute, University Hospitals Leuven, KU Leuven, Leuven, Belgium

**Aim:** To evaluate whether whole-body diffusion-weighted magnetic resonance imaging (WB-DWI) is able to correctly predict operability in patients with colorectal cancer and peritoneal metastases.

**Methods:** Sixty patients diagnosed with colorectal cancer and peritoneal metastases underwent WB-DWI prior to laparotomy in this prospective single centre study. The peritoneal cancer index (PCI) was estimated on WB-DWI and compared to PCI during laparotomy. Furthermore, presence of nodal and/or distant metastases was evaluated. Histopathology after surgery or biopsy was used as primary reference standard.

**Results:** WB-DWI correctly predicted operability in 54 of 60 patients (90 %). In 6 patients the disease extent was underestimated by WB-DWI (in 5 patients the predicted PCI was too low, in 1 patient a periportal adenopathy was missed). In none of the patients WB-DWI overestimated the PCI, thus none of the patients were incorrectly deprived from surgery. Eighteen patients had irresectable distant metastases. WB-DWI correctly identified 17 of these 18 patients (94 %).

**Conclusion:** WB-DWI is a reliable staging method for surgical planning in patients with colorectal cancer and peritoneal metastases. Our results need to be confirmed in larger studies.

### S3 Role of apparent diffusion coefficient (ADC) diffusion-weighted MRI for predicting extra capsular extension of prostate cancer

#### P. Pricolo, S. Alessi, P. Summers, E. Tagliabue, G. Petralia

##### European Institute of Oncology, Milano, Italy

###### **Correspondence:** P. Pricolo (paola.pricolo@ieo.it) – European Institute of Oncology, Milano, Italy

**Aim:** To evaluate the potential of the apparent diffusion coefficient (ADC) in predicting extracapsular extension (≥ pT3a) of prostate cancer (PCa).

**Materials and methods:** We analysed 301 consecutive PCa patients (137 low-risk and 164 intermediate/high-risk by EAU classification) who underwent pre-surgical multiparametric MRI. The index lesion was assigned a score (from 1-5) for its probability of being ≥ pT3a based on the T2-weighted images (ECE score) and its ADC value measured. An ADC value cut-off for predicting ≥ pT3a was obtained from ROC analysis. Diagnostic performances of risk groups, ECE score and ADC (best cut-off) of index lesion for predicting ≥ pT3a were calculated. The effects of risk group, ECE score and ADC value (best cut-off) for predicting ≥ pT3a were calculated on a multivariate analysis.

**Results**: 119 patients were ≥ pT3a and 182 patients < pT3a at surgery. The ADC cut-off value (1031 μm^2^/s) showed an ability to rule out (Sensitivity, negative predictive values, negative likelihood ratio) and rule in (specificity, positive predictive value, positive likelihood ratio) ≥ pT3a respectively of 85 %/86 %/0.24 and 63 %/60 %/2.27. The corresponding values for the ECE score were 78 %/85 %/0.26 and 83 %/75 %/4.59 and those for risk group were 76 %/80 %/0.39 to 60 %/ 55 %/1.91 respectively. The addition of the ADC value to risk group and to ECE score improved the ability to predict ≥ pT3a (AUC from 0.85 to 0.87; p <0.0001).

**Conclusions**: The index lesion ADC value showed an additional value in predicting ≥ pT3a to risk group classification and to the standard assessment of extracapsularity based on T2-weighted images.

### S4 Generating evidence for clinical benefit of PET/CT – are management studies sufficient as surrogate for patient outcome?

#### C. Pfannenberg^1^, B. Gückel ^1^, SC Schüle^1^, AC Müller ^2^, S. Kaufmann^1^, N. Schwenzer^1^, M. Reimold ^3^,C. la Fougere^3^, K. Nikolaou^1^, P. Martus^4^

##### ^1^Department of Diagnostic and Interventional Radiology, University Tuebingen, Tübingen, Germany; ^2^Clinic of Radiation Oncology, University Tuebingen, Tübingen, Germany; ^3^Department of Nuclear Medicine, University Tuebingen, Tübingen, Germany; ^4^Institute of Clinical Epidemiology and applied Biostatistics, University Tuebingen, Tübingen, Germany

###### **Correspondence:** C. Pfannenberg (christina.pfannenberg@med.uni-tuebingen.de) – Department of Diagnostic and Interventional Radiology, University Tuebingen, Tübingen, Germany

**Aim:** To evaluate the impact of PET/CT on clinical management in daily routine based on a prospective data registry and to link these information with the expected benefit on patient outcome (“linked evidence approach”).

**Methods:** A prospective patient cohort (04/2013-04/2015) having a PET/CT for oncological reasons was evaluated based on questionnaires from referring physicians on intended management pre- and post-PET/CT. Primary endpoint was the impact of PET/CT on management regarding treatment or further investigations in different indications and cancer types. Secondary endpoint was to develop a model linking the management changes (=intermediate outcome) with patient outcome.

**Results:** 2692 patients were evaluated (61 ± 15 ys, 64 % m). Lung, prostate, melanoma, NET and lymphoma accounted for 2/3 of cases. The most frequent PET/CT indication was staging (62 %). Overall, physicians changed their intended management in 58 % of patients (95 % CI, 56 %-60 %) based on PET/CT results. In 24 % of all cases (22 % prostate, 34 % melanoma) management changed from non-treatment (e.g. watching, additional imaging) to a post PET/CT treatment strategy. The impact of PET/CT was highest in reducing demands for additional testing. Uncertainties regarding treatment goal (curative vs palliative) were reduced by 35 %. By linking registry data with FU data, first in melanoma, the impact of PET/CT on outcome could be demonstrated (RO metastasectomy).

**Conclusion:** Registry data confirmed that physicians often change their intended management on the basis of PET/CT. Based on certain assumptions the “linked evidence” approach could be used as a model to generate evidence for the clinical benefit of PET/CT.

### S5 Heterogeneity of treatment response in skeletal metastases from breast cancer with 18F-fluoride and 18F-FDG PET

#### GJ Cook, GK Azad, BP Taylor, M Siddique, J John, J Mansi, M Harries, V Goh

##### Cancer Imaging Dept, King’s College London and Guys & St Thomas’ Hospitals, London, UK

###### **Correspondence:** GJ Cook (gary.cook@kcl.ac.uk) – Cancer Imaging Dept, King’s College London and Guys & St Thomas’ Hospitals, London, UK

**Purpose:** To evaluate heterogeneity of response in skeletal metastases from breast cancer with 18F-fluoride and 18F-FDG PET.

**Method:** Baseline and 8 week 18F-fluoride and 18F-FDG PET scans were analysed in 8 females (mean age 65.6 years) taking part in a multimodality treatment response study. SUVmax was measured in up to 5 metastases in each patient (33 lesions) and EORTC PET criteria used for response categorisation (SD, PR, PD). Clinical response was measured up to 24 weeks by conventional biochemical and imaging methods (bone scan, CT, pain score, biochemistry, tumour markers).

**Results:** Less than half of the skeletal metastases showed concordant response categorisation between 18F-fluoride and 18F-FDG (14/33 lesions) with 15/33 lesions showing minor discordance and 4/33 lesions complete discordance. 6 patients showed interlesional response categorisation heterogeneity with 18F-fluoride and 5 with 18F-FDG, PD and PR lesions coexisting in 2 patients in each group. In patients with 24 week clinical PR, 8/19 18F-fluoride and 11/19 18F-FDG lesions showed PR. In those with clinical PD, 2/10 18F-fluoride and 0/10 18F-FDG lesions showed PD. 10/13 ^18^F-fluoride lesions in patients with a clinical PR showed a flare at 8 weeks that reduced by 12 weeks.

**Conclusion:** There is significant discordance between 18F-fluoride and 18F-FDG PET response assessment in breast cancer skeletal metastases at 8 weeks. ^18^FDG PET is better at predicting response at 8 weeks but neither tracer can reliably predict progressive disease at this early time point. Within individual patients there is frequent interlesional response heterogeneity with both tracers.

### S6 Accuracy of suspicious breast imaging—can we tell the patient?

#### S Seth, R Burgul, A Seth

##### Forth Valley Royal Hospital, Scotland, UK

###### **Correspondence:** A Seth (archana.seth@nhs.net) – Forth Valley Royal Hospital, Scotland, UK

**Aim**: To determine if suspicious breast imaging can be used to inform the patients of the diagnosis of breast cancer, at first one-stop clinic attendance.

**Methods**: List of breast cancers diagnosed in year 2013 was obtained from the audit department. Radiology database and clinical portal were used to review clinical requests and imaging. Caldicott approval was obtained. Breast imaging results were analysed and correlated with core biopsy results and final histopathology. For all patients with benign imaging, radiology database was checked for 2 years to ensure no representation with cancer. Accuracy of imaging in the diagnosis of breast cancer and positive predictive value (PPV) of abnormal/suspicious imaging was calculated.

**Results**: Over 3800 patients had imaging in 2013. Of these, 198 patients were diagnosed with breast cancer. Imaging was indeterminate/suspicious/positive for malignancy in 93.4 % (185 cases) of cases of proven malignant diagnosis. 82.3 % [n = 163] patients had suspicious imaging with biopsy proven diagnosis of breast cancer. There were 4 false positive cases (suspicious imaging but benign histology). Two cases had B3 core biopsy results and there was 1 case of parenchymal distortion. Excision was recommended for all three cases. There was 1 case of peri-ductal mastitis, which was suspicious on radiology. PPV of suspicious radiology for breast malignancy was 97.6 % PPV of suspicious radiology for surgery was 99.4 %

**Conclusion**: Suspicious radiology (overall imaging opinion of 4 or above) has high PPV for diagnosis of breast cancer and/or surgery. This can be used reliably to inform the patients of their likely diagnosis.

### S7 Measurement method of tumour volume changes during neoadjuvant chemotherapy affects ability to predict pathological response

#### S Waugh^1^, N Muhammad Gowdh^2^, C Purdie^3^, A Evans^2^, E Crowe^4^, A Thompson^5^, S Vinnicombe^2^

##### ^1^Dept of Medical Physics, Ninewells Hospital, Dundee, UK; ^2^Dept of Breast Imaging, Ninewells Hospital, Dundee, UK; ^3^Dept of Pathology, Ninewells Hospital, Dundee, UK; ^4^Dept of Clinical Radiology, Ninewells Hospital, Dundee, UK; ^5^Department of Surgical Oncology, MD Anderson Cancer Centre, Houston, USA

###### **Correspondence:** S Waugh (shelley.waugh@nhs.net) – Dept of Medical Physics, Ninewells Hospital, Dundee, UK

**Aim**: To assess whether changes in semi- and fully automated measures of tumour volume between baseline and interim magnetic resonance imaging (MRI) are equivalent in predicting pathological response to neoadjuvant chemotherapy (NAC) for primary breast cancer, focusing on the accurate prediction of minimal residual disease.

**Methods**: 78 patients undergoing NAC underwent contrast-enhanced MRI before and after 3 cycles of treatment. Tumour volumes were measured using fully- (BreVis; FTV) and semi-automated (ITK-Snap; ETV) methods. Two-minute post-contrast subtracted images were analysed for ETV. ETV intra- and inter-observer reproducibility was assessed with calculation of the coefficient of reproducibility (CoR). Percentage reduction in FTV and ETV between baseline and interim MRI was compared with final pathological response using the residual cancer burden score (RCB) on resected specimens, and significance of any differences was assessed with Mann-Whitney U tests.

**Results**: There was good correlation between ETV and FTV (r = 0.744). Intra and inter observer CoR for ETV was 11.6 % and 14.8 % respectively. Mean % reductions in ETV by pathological response were: pCR 96.4 % (n = 12), RCB-I 66.6 % (n = 10), RCB-II 62.9 % (n = 39), RCB-III 27.3 % (n = 17). Corresponding values for FTV were: 88.8 %, 70.6 %, 54.6 % and 20.8 %. Significant differences in % ETV changes were found for pCR vs. RCB-I, II & III (all p < 0.008). For FTV, significant differences were measured only for pCR vs. RCB-II & III (both p < 0.001).

**Conclusion**: ETV changes between baseline and interim MRI differentiate better between pCR and minimal residual disease than FTV. Such differentiation could facilitate novel approaches to surgical management post NAC.

### S8 Diagnostic yield of CT IVU in haematuria screening

#### F. Arfeen, T. Campion, E. Goldstraw

##### Homerton Hospital, Homerton Row, London, UK

###### **Correspondence:** F. Arfeen (farrukharfeen@doctors.org.uk) – Homerton Hospital, Homerton Row, London, UK

**Aims**: The aim of this study was to determine the diagnostic yield of urothelial malignancy in patients undergoing haematuria screening with CT intravenous urography (IVU).

**Methods**: A retrospective study reviewed 332 consecutive patients undergoing CT IVU at the Homerton Hospital over a six-month period (July to December 2015. Inclusion criteria were only haematuria screening patients Demographic details, whether haematuria was macroscopic or microscopic, cause for haematuria (if present), and significant incidental findings, were recorded.

**Results**: A total of 256 patients met the inclusion criteria. The average age was 59 (range 26 - 95) with 56 % (144/256) men and 44 % (112/256) women. Of these, 52 % (132/256) had macroscopic and 44 % (113/256) had microscopic haematuria (not recorded in 4 %). A cause for haematuria was determined in 14 % of patients (35/256). Malignancy was seen in 3 % (7/256), of which bladder cancer accounted for 1.5 % (4/256) and renal cancer for 1.2 % (3/256). No ureteric malignancy was detected. A malignant cause for macroscopic haematuria was seen in 4 % (5/132) and for microscopic haematuria in 2 % (2/113) patients. Renal tract calculi accounted for haematuria in the remaining 11 % (28/256).

**Conclusion**: The results demonstrate likely overuse of CT IVU with low diagnostic yield in haematuria screening. With only 14 % demonstrating a cause and no ureteric malignancy evident, better resource management is seemingly possible. No current consensus guidelines are available and, after review of current literature, we recommend the use of stricter criteria for imaging.

### S9 Percutaneous radiofrequency ablation of unresectable locally advanced pancreatic cancer: preliminary results

#### D’Onofrio M, Ciaravino V, Crosara S, De Robertis R, Pozzi Mucelli R

##### University Hospital G.B. Rossi, Verona, Italy

###### **Correspondence:** D’Onofrio M (Mirko.donofrio@univr.it) – University Hospital G.B. Rossi, Verona, Italy

**Aims**: The objective of this study was to evaluate the efficacy of percutaneous radiofrequency ablation of locally advanced pancreatic cancer located in the pancreatic body.

**Materials and Methods:** Patients with biopsy-proven locally advanced pancreatic adenocarcinoma were considered for percutaneous radiofrequency ablation. Post-procedural CT studies and Ca19.9 tumour marker evaluation were performed at 24 hours and 1 month. At CT treatment effect was evaluated excluding the presence of complications. The technical success of the procedure is defined at CT as the achievement of tumoural ablated area.

**Results**: 23 patients have been included in the study. 5/23 of the patients were excluded. At CT the mean size of the intra-lesional post-ablation necrotic area was 32 mm (range 15 – 65 mm). Technical success of the procedure has been obtained in (16/18) 93 % of cases. None of the patients developed post-procedural complications. Mean Ca19.9 serum levels one day before, one day after and one month after the procedure were respectively 285,8 U/ml (range 16,6–942,0 U/ml), 635,2 U/ml (range 17,9–3368,0 U/ml) and 336,0 U/ml (range 7,0–1400,0 U/ml). Follow-up duration was less than 6 months for 11 patients, more than 6 months for 7 patients. At the time of the draft of this paper, the mean survival of the patients included in the study was 185 days (range 62–398 days). 

**Conclusion:** Percutaneous radiofrequency ablation of locally advanced adenocarcinoma has an high technical success rate and is effective in cytoreduction both at imaging and laboratory controls.

### S10 Iodine maps from dual energy CT improve detection of metastases in staging examinations of melanoma patients

#### M. Uhrig, D. Simons, H. Schlemmer

##### German Cancer Research Center Heidelberg, Germany

###### **Correspondence:** M. Uhrig (m.uhrig@dkfz.de) – German Cancer Research Center Heidelberg, Germany

**Aim**: Staging of melanoma patients by CT is challenging, because metastases may hide in every part of the body and can exhibit a similar density to some body own tissues, e.g. musculature. Iodine maps (IM) from dual energy CT (DECT) highlight enhancing lesions and visualise their spatial contrast medium distribution. Purpose of this study was to evaluate whether IM improve lesion detection in staging examinations of melanoma patients.

**Methods**: 75 DECT scans (thorax (T) and abdomen (A)) from 75 melanoma patients were retrospectively analyzed. For each patient 3 conventional image reconstructions (cCT) (T: lung kernel 1 mm axial and soft tissue kernel 3 mm axial, A: soft tissue kernel 3 mm axial) as well as 3 IM (T: 1 mm and 3 mm axial, A: 3 mm axial) were provided. A radiologist reported the number of lesions detected additionally by analysing the IM following cCT.

**Results**: Total 44 lesions (17 metastases) in 29 patients were additionally detected on IM. All lesions could retrospectively be identified on cCT and were located in the liver (34 %), inter or intramuscular (23 %), subcutaneous (9 %), lung (7 %), mesenterial (5 %), intestinal (5 %), mediastinal (5 %), skeleton (4 %), pancreas (2 %), vagina (2 %), supraclavicular (2 %) and peritoneal (2 %). Lung findings included 2 pulmonary emboli.

**Conclusion**: IM from DECT improve detection of metastases and relevant secondary findings in staging examinations of melanoma patients.

### S11 Can contrast enhanced CT predict pelvic nodal status in malignant melanoma of the lower limb?

#### Kate Downey (kate.downey@me.com)

##### The Royal Marsden Hospital, London UK

**Aim:** At our institution iliac lymph node (LN) dissection for melanoma is often performed concurrently with inguinal LN dissection. If CT can accurately stage pelvic lymph nodes above the inguinal ligament a subset of patients may be spared iliac node dissection and its associated morbidity. We evaluate the accuracy of CT in LN staging and the predictive value of individual CT imaging features of inguinal and pelvic LN metastases using histology as a gold standard.

**Method:** In a retrospective analysis of 92 consecutive patients, LN characteristics on CT; size, a rounded morphology, the presence of extracapsular spread, abnormal contrast enhancement, cortical nodularity and a fatty hilum, within the inguinal, cloquet, external iliac, side-wall, common iliac and para-aortic LN groups were compared to histology (Fishers exact test).

**Results:** LN size on CT was predictive of pathological involvement (p < 0.001) although a rounded morphology was not. The sensitivity of extracapsular spread within the inguinal LN group was high (100 %, p = 0.52) and predictive of pelvic LN spread (p = 0.009). CT was sensitive for inguinal and common iliac (100 %) as well as external iliac (83 %) but less sensitive for cloquet (67 %) and side-wall involvement (67 %). The presence of cortical nodularity or the absence of a fatty hilum were not helpful.

**Conclusion:** CT can help predict pelvic nodal status in patients with malignant melanoma of the lower limb and the presence of extracapsular spread in inguinal LN group is suggestive of more proximal disease.

### S12 Current practice in the investigation for suspected Paraneoplastic Neurological Syndromes (PNS) and positive malignancy yield

#### S Murdoch, AS Al-adhami, S Viswanathan

##### Glasgow Royal Infirmary, Scotland, UK

###### **Correspondence:** AS Al-adhami (abdullahaladhami@gmail.com) – Glasgow Royal Infirmary, Scotland, UK

**Aim:** To assess the positive yield for malignancy in imaging investigations for suspected paraneoplastic neurological syndrome.

**Method**: Study performed in Institute of Neurological Sciences, a tertiary neurological centre serving 2.7 million in the west of Scotland. Obtained list of all inpatients who had a CT Chest abdomen and pelvis (CT CAP) done for suspected PNS over a 16 month period. Case notes, clinic letters, other imaging investigations and autoantibody tests were retrospectively reviewed. Patients’ initial neurological symptoms, ultimate neurological diagnosis and presence of a malignancy after a minimum of 1-year follow-up were recorded.

**Results**: 75 patients had a CT for suspected PNS (Excluding myasthenia gravis), 16 of which had follow-up PET-CT, 3 Mammograms, 5 Testicular ultrasounds and 6 repeat CT CAP. Only three relevant tumours detected (4 %), two on initial CT CAP and one on subsequent PET-CT. One incidental breast cancer found, unrelated to patient’s neurology. Only two of those with no cancer detected after one-year follow-up are still suspected to be paraneoplastic, with the remaining 69 cases given an alternative neurological diagnosis.

**Conclusion**: There is very little in literature on expected positive yield of malignancy for suspected PNS. However, our study found the yield to be very small. Many of those scanned had nonspecific neurology rather than recognised PNS. Given the significant amount of radiation involved, we felt there is a need for an agreed guideline on which patients should be investigated.

## Poster presentations

### P1 Technical success and efficacy of pulmonary radiofrequency ablation: an analysis of 207 ablations

#### S Smith, P Jennings, D Bowers, R Soomal

##### Ipswich Hospital, University of Suffolk, Heath Rd, Ipswich, Suffolk, UK

###### **Correspondence:** S Smith (simon.smith@ipswichhospital.nhs.uk) – Ipswich Hospital, University of Suffolk, Heath Rd, Ipswich, Suffolk, UK

**Aim**: To analyse the technical success of ablation therapy and the incidence of complications in patients treated with pulmonary ablation at a District General Hospital without on-site cardiothoracic support. This poster will present the complication profile in this patient group. Management of complications will be discussed.

**Materials and methods:** Technical success and complications in all patients undergoing lung ablation between June 2009 and July 2015 was recorded. Ablation was performed under deep conscious sedation according to standard protocols.

**Results**: 207 pulmonary ablations were performed in 86 patients at 156 attendances. A variety of tumour types were treated.

Technical success was achieved in 207/207 (100 %).

- 30 and 90 day mortality was 0 %.
- 35/86 (40.7 %) patients were treated as day cases. None were readmitted.
- 84/86 (97.7 %) patients were discharged within 24 hours of the ablation.

The major complication (pneumothorax requiring chest drain, severe chest pain and symptomatic pseudoaneurysm) rate was 13/86 (15 %).

- 2/86 (2.3 %) patients developed psuedoaneurysms.
- 15/86 (17.4 %) patients had a significant pneumothorax.
- 6/86 (7.0 %) patients required a chest drain.
- 6/86 (7.0 %) patients described significant chest pain.
- 1/86 (1.1 %) developed an asymptomatic rib fracture.

**Conclusion**: RFA is a safe and effective procedure that can be performed without on-site cardiothoracic support.

### P2 Lesion control and patient outcome: prospective analysis of radiofrequency abaltion in pulmonary colorectal cancer metastatic disease

#### S Smith, P Jennings, D Bowers, R Soomal

##### Ipswich Hospital, University of Suffolk, Heath Rd, Ipswich, Suffolk, UK

###### **Correspondence:** S Smith (simon.smith@ipswichhospital.nhs.uk) – Ipswich Hospital, University of Suffolk, Heath Rd, Ipswich, Suffolk, UK

**Aim**: to assess factors affecting local disease control and patient survival in patients with pulmonary metastases from colorectal cancer treated with percutaneous radiofrequency ablation.

**Methods**: Using a prospective database overall survival and local disease control was calculated.

Factors influencing outcome were explored.

**Results**: 101 pulmonary metastases were treated in 46 patients.

- An average of 2.2 lesions per patient (range 1 to 8).
- The median diameter was 9 mm (range 3-29 mm).
- 18 (40.9 %) had bilateral metastases.
- 13 (29.5 %) had extrapulmonary metastatic disease.

Mean survival time of 53.58 +/- SE3.47 months with a 1, 2, 3, 4 and 5 year survival of 97.4 %, 91.3 %, 81.5 %, 59.8 % and 48.0 %.

There was no statistically significant difference in survival with regards to:

- time to development of metastatic disease.
- the total number of lesions ablated.
- the initial number of lesions ablated.
- the maximum size of lesion treated.
- unilateral vs bilateral disease.

Patients with extra-pulmonary disease had a shorter survival from primary diagnosis.

23 (22.8 %) of the 101 lesions were found to have progressed after first RFA. The mean time to recurrence was 9.6 +/- SD7.7 months (median 8 months, range 1 – 38 months).

- The mean initial size of the progressed lesions was greater than the mean initial size of the stable lesions p = 0.002.
- Local relapse was more likely when a metastases was close to a large (>3 mm) vessel (p = 0.002).

**Conclusion**: Ablation of pulmonary metastases in selected patients can improve patient outcome. Lesion size and proximity to large vessels are important considerations when planning treatment.

### P3 Hepatocellular carcinoma in a post-TB patient: case of tropical infections and oncologic imaging challenges

#### TM Mutala, AO Odhiambo, N Harish

##### University of Nairobi, Nairobi, Kenya

###### **Correspondence:** TM Mutala (musilamutala@gmail.com) – University of Nairobi, Nairobi, Kenya

**Aim**: We describe a case study that demonstrates radiology can correctly predict a malignant process in a mass forming infectious clinical background.

**Methods**: We analysed clinical findings of a 34 year old male patient who had successfully completed treatment for pulmonary TB with suspected multisytemic relapse. Chest radiograph, chest and head CT scans were ordered. Tissue diagnosis of an easily accessible intracranial lesion was done on radiological recommendation.

**Results**: A large right occipital lobe mass and other satellite smaller lesions within the contralateral frontal lobe were seen on the cranial CT. In the chest right apical findings consistent with post-TB changes and bilateral multiple rounded opacities within the lung parenchyma were seen. An irregular mass within the visualised right liver lobe was also noted. Pre-contrast images had attenuation indicating haemorrhage within the lesions. These features strongly suggested a radiological diagnosis of metastatic disease rather than TB from our experience. Histologic diagnosis of the large brain lesion was a malignant epithelial tumour with high vascularity and necrosis. Immunohistochemistry tested positive for hepatocyte paraffin (Hep Par 1) and cytokeratin AE1/AE3 antibodies. Clinical and serological findings had not depicted higher risk for HCC.

**Conclusion**: In tropical Africa mass forming infectious diseases are very common radiological findings. Radiologists must be knowledgeable of the patterns that are more predictive of a malignant lesion even when the clinical information suggests an infective process.

### P4 Role of apparent diffusion coefficient (ADC) diffusion-weighted MRI for predicting extracapsular extension of prostate cancer

#### P. Pricolo, S. Alessi, P. Summers, E. Tagliabue, G. Petralia

##### European Institute of Oncology, Via Ripamonti 435, Milano, Italy

###### **Correspondence:** P. Pricolo (paola.pricolo@ieo.it) – European Institute of Oncology, Via Ripamonti 435, Milano, Italy

**Aim:** To evaluate the potential of the apparent diffusion coefficient (ADC) in predicting extracapsular extension (≥ pT3a) of prostate cancer (PCa).

**Materials and methods:** We analysed 301 consecutive PCa patients (137 low-risk and 164 intermediate/high-risk by EAU classification) who underwent pre-surgical multiparametric MRI. The index lesion was assigned a score (from 1-5) for its probability of being ≥ pT3a based on the T2-weighted images (ECE score) and its ADC value measured. An ADC value cut-off for predicting ≥ pT3a was obtained from ROC analysis. Diagnostic performances of risk groups, ECE score and ADC (best cut-off) of index lesion for predicting ≥ pT3a were calculated. The effects of risk group, ECE score and ADC value (best cut-off) for predicting ≥ pT3a were calculated on a multivariate analysis.

**Results**: 119 patients were ≥ pT3a and 182 patients < pT3a at surgery. The ADC cut-off value (1031 μm^2^/s) showed an ability to rule out (Sensitivity, negative predictive values, negative likelihood ratio) and rule in (specificity, positive predictive value, positive likelihood ratio) ≥ pT3a respectively of 85 %/ 86 %/ 0.24 and 63 %/ 60 %/2.27. The corresponding values for the ECE score were 78 %/ 85 %/ 0.26 and 83 %/ 75 %/ 4.59 and those for risk group were 76 %/ 80 %/ 0.39 to 60 %/ 55 %/1.91 respectively. The addition of the ADC value to risk group and to ECE score improved the ability to predict ≥ pT3a (AUC from 0.85 to 0.87; p <0.0001).

**Conclusions**: The index lesion ADC value showed an additional value in predicting ≥ pT3a to risk group classification and to the standard assessment of extracapsularity based on T2-weighted images.

### P5 What a difference a decade makes; comparison of lung biopsies in Glasgow 2005 and 2015

#### M. Hall, M. Sproule, S. Sheridan

##### Queen Elizabeth University Hospital, 1345 Govan Rd, Glasgow G51 4TF, UK

###### **Correspondence:** M. Hall (markhall5@nhs.net) – Queen Elizabeth University Hospital, 1345 Govan Rd, Glasgow G51 4TF, UK

**Aim**: To audit and compare the change in lung cancer histopathology and biopsy practice over a decade in a Glasgow hospital with reference to the standards set by the British Thoracic Society.

**Materials and methods:** All the lung biopsies performed in the west of Glasgow in 2005 and 2015 were analysed looking at patient demographics, lesion characteristics, complication rates and sample histopathology. The results for 2005 and 2015 were compared to each other to assess change in tumour type and clinical practice audited against the current BTS guidelines.

**Results**: 89 lung biopsies were carried out in 2005 and 106 in 2015. We found that there was a rise in the incidence of adenocarcinoma and a decrease in incidence of small cell carcinoma. The mean lesion size had decreased, depth from the skin increased and there was a marked increase in the percentage of biopsied lesions measuring 1 cm or less (2 % to 32 %). The complication rate for both years was within the reference standard set by the BTS and showed little interval change.

**Conclusions**: In our population we have shown over 10 years a shift in cancer histopathology, with a rise in pulmonary adenocarcinoma. We found a trend to performing biopsies on smaller and deeper lesions. This may be due to earlier cancer detection, and a greater push from clinicians to obtain definitive histology.

### P6 Solid pseudopapillary tumour of pancreas: imaging features of a rare neoplasm

#### KY Thein, CH Tan, YL Thian, CM Ho

##### Khoo Teck Puat Hospital, Alexandra Health Group, Yishun, Singapore

###### **Correspondence:** KY Thein (kythein@yahoo.com) – Khoo Teck Puat Hospital, Alexandra Health Group, Yishun, Singapore

**Aim:** Solid pseudopapillary tumour of the pancreas is a rare exocrine tumour with low malignant potential accounting for 0.2-2.7 % of all primary pancreatic neoplasms. It is frequently asymptomatic and usually detected when it has grown to a large size.

It is typically seen on imaging as a well-encapsulated mass with varying solid and cystic components caused by hemorrhagic degeneration. Calcifications and enhancing solid areas may present at the periphery of the mass. It usually located in the pancreatic body and tail. We aim to describe imaging findings in patients with pathologically-confirmed solid pseudopapillary tumour of the pancreas.

**Methods:** 6 patients with pathologically-confirmed solid pseudopapillary tumour of the pancreas from 4 institutions in Singapore within the last 5 years were reviewed. There were 1 male and 5 females with age range from 9 to 62 years. Multiphasic CT for all 6 cases and multiphasic MRI for 1case were performed.

**Results:** 3 patients were asymptomatic and the other 3 presented with abdominal pain. Imaging patterns were well-encapsulated mass (5 cases), ill-defined mass with extracapsular invasion (1 case), hypodense mass with varying solid and cystic component (all 6 cases), peripheral calcification (2 cases), enhancing peripheral solid component (all 6 cases), located in body or tail (all 6 cases).

**Conclusion:** Most of our cases showed typical appearances with some atypical imaging features. Recognising their imaging features will help early diagnosis.

### P7 MDCT - pathological correlation in colon adenocarcinoma staging: preliminary experience

#### S De Luca, C Carrera, V Blanchet, L Alarcón, E Eyheremnedy

##### Hospital Aleman, Buenos Aires, Argentina

###### **Correspondence:** S De Luca (sdeluca@hospitalaleman.com) – Hospital Aleman, Buenos Aires, Argentina

**Introduction**: Colon cancer represents the fourth leading cause of cancer death worldwide. In recent years, medical practice has started implementing the concept of “Locally advanced colon cancer" for cancer patients with T3, T4 or N positive.

Since neo-adjuvant therapy was proven beneficial in cases of other locally advanced tumours, the question of whether it could also benefit patients with locally advanced colon cancer has arisen.

**Objectives**: Determine whether preoperative staging with MDCT can predict the TN stage of the disease.

**Materials and methods**: We included 39 MDCT of patients who went through a primary colon tumour resection during the 2013-2016 period.

Patients with visible metastases were excluded. The preoperative CT scans were reviewed independently by two radiologists who were unaware of the histological stage.

**Results**: The observer A correctly predicted 64 % of cases for T and 46 % for the N stages. The observer B predicted 56 % for T and 12 % for N stages.

The agreement between observers for the prognostic stage was 66 % for T and 4 % for N. Both observers agreed among themselves and with the final pathology in 51 % and 5 % of cases for T and N, respectively.

**Conclusion**: MDCT is an accurate method for staging pre-surgical colorectal cancer patients. The diagnostic yield is particularly high in the identification T stage becoming lower in N staging, where greater variability between observers is obtained. Recognising locally-advanced-tumours in the preoperative.

### P8 Image guided biopsy of thoracic masses and reduction of pneumothorax risk: 25 years experience

#### B K Choudhury, K Bujarbarua, G Barman

##### Dr. B. Borooah Cancer Institute, Guwahati, India

###### **Correspondence:** B K Choudhury (bkchoudhury@gmail.com) – Dr. B. Borooah Cancer Institute, Guwahati, India

**Aim**: The purpose of our study was to determine accuracy and reliability of image guided thoracic biopsy and to know the efficacy of uses of different measures to reduce the risk of pneumothorax.

**Materials and methods**: Fine needle aspiration biopsies were performed in 1650 patients from 1989 to 2014 using Fluoroscopy, Ultrasound and CT as Image guidance. After reviewing the patient, chest skiagram, CT and coagulation profile an appropriate plan was formulated for safest approach to the lesion by a fine needle. Several measures were applied to reduce the risk of pneumothorax. Immediately after the procedure puncture site was put on dependant position. Other measures included accurate and careful manipulation of needle, extra pleural approach, widening of extrapleural space, pathway through non-aerated lung, use of small bore needle etc. All patients underwent chest radiography to detect a pneumothorax.

**Results:** Fluoroscopy, US and CT as guidance were used in 170 (10.30 %), 270 (16.36 %) and 1210 (73.33 %) cases respectively. Results obtained in 1419 cases (86 %). Malignant cases were 1078 (76 %). Common complication was pneumothorax and occurred in 38 cases (2.3 %). Only three cases required placement of a chest tube, rest were small and resolved spontaneously. Incidence of pneumothorax dropped significantly after using several measures.

**Conclusion**: Image guided biopsy is a safe and reliable method in tissue diagnosis of thoracic lesions with low morbidity and high accuracy. Most common complication is pneumothorax. Pneumothorax can be significantly reduced by meticulous planning using several measures.

### P9 Tumour heterogeneity analysis of 18F-FDG-PET for characterisation of malignant peripheral nerve sheath tumours in neurofibromatosis-1

#### GJ Cook, E Lovat, M Siddique, V Goh, R Ferner, VS Warbey

##### Cancer Imaging Department, King’s College London and Guys & St Thomas’ Hospitals, London, UK

###### **Correspondence:** GJ Cook (gary.cook@kcl.ac.uk) – Cancer Imaging Department, King’s College London and Guys & St Thomas’ Hospitals, London, UK

**Purpose:** 18F-FDG PET texture analysis (TA) provides a measure of tumour heterogeneity but it is unknown if it can differentiate benign and malignant peripheral nerve sheath tumours (PNSTs) in patients with neurofibromatosis-1, a frequent diagnostic dilemma. We compared TA with maximum standardised uptake value (SUVmax) in 18F-FDG PET for this purpose.

**Methods:** 18F-FDG PET was performed at 90 and 240mins post-injection in 55 patients with neurofibromatosis-1 and suspected malignant transformation (M:F 30:25, mean age 34.9 years). All patients had a histological reference standard. TA was performed at both time-points measuring 1^st^, 2^nd^, high-order and model-based texture parameters. Statistical comparisons were made using ROC analysis.

**Results:** 31 benign and 24 malignant lesions were confirmed histopathologically. AUROC was greatest for SUVmax at 90 and 240 min time-points (0.991, 3 false-positives and 0.996, 2 false-positives, respectively). Standard deviation (SD), entropy, fractal dimension (FD) and coarseness performed well (0.965, 0.991; 0.942, 0.952; 0.804, 0.81; 0.890, 0.891). The greatest AUROC resulted from the combined parameter SUVmax/FD (0.997, 0.996) with only one false positive result and no false negatives.

**Conclusions:** TA does not improve discrimination of benign and malignant PNSTs further compared to SUVmax. However, a number of texture features measuring tumour heterogeneity show good discrimination and using a combined parameter (SUVmax/FD) maximises diagnostic accuracy.

### P10 Impact of introduction of vacuum assisted excision (VAE) on screen detected high risk breast lesions

#### L Potti, B Kaye, A Beattie, K Dutton

##### North Cumbria University Hospitals NHS Trust, Cumberland Infirmary, Newtown Road, Carlisle, Cumbria, CA2 7HY, UK

###### **Correspondence:** L Potti (drlekhap@yahoo.com) – North Cumbria University Hospitals NHS Trust, Cumberland Infirmary, Newtown Road, Carlisle, Cumbria, CA2 7HY, UK

**Aim**: Prior to the introduction of VAE, diagnostic surgical excision was required for high risk (B3) breast lesions. Our aim was to find if the introduction of VAE reduced the number of diagnostic surgical excision biopsies in our unit.

**Methods**: We conducted an audit over a one year period, Feb 2015 to Jan 2016, at our institution. From our records we identified all patients with B3 or B4 diagnosis in the initial (10G or 14G) biopsy who then went on to have 7G VAE or diagnostic surgical excision biopsies.

**Results**: 21 patients in the breast screening programme were identified to have B3 or B4 lesions. 18 of these patients had VAE and 3 had diagnostic surgical excision. Of these 3, the first patient declined VAE, the second patient opted for therapeutic surgery without further biopsy and the procedure was unsuccessful in the third patient due to technical difficulty.

Of the 18 cases that had VAE, three cases were upgraded to B5 and underwent therapeutic surgery. Eight cases were downgraded to B2 and seven cases remained B3. Therefore we avoided surgery in 15 out of 21 patients.

Our study demonstrates that 7G VAE has reduced the number of diagnostic surgeries for high risk breast lesions. The number of referrals to the regional centre for the procedure has also reduced since the availability of the equipment and expertise at our local unit.

### P11 Can we reduce prevalent recall rate in breast screening?

#### AA Seth, F Constantinidis, H Dobson

##### West of Scotland Breast Screening Centre, Glasgow, UK

###### **Correspondence:** AA Seth (archana.seth@nhs.net) – West of Scotland Breast Screening Centre, Glasgow, UK

**Aim:** To reduce prevalent recall rate and unnecessary false positive assessments using risk stratification scoring system.

**Materials-methods:** Retrospective review of 2013-2014 local screening database. Number of women at assessment clinics, initial mammographic concern (R3-probably benign, R3-indeterminate, R4-suspicious and R5-malignant), numbers of biopsies and surgical referrals were recorded and the recall Positive Predictive Value (PPV) for each category was calculated.

**Results:** 2965 women attended the assessment clinic (43 % prevalent round). Prevalent recall rate was 10.3 %, with PPV of recall being 9.6 % compared to 25 % on the incident screen.

10.6 % of mammograms were categorised as R3-probably benign with low PPV of recall at 1.6 %.

Largest subgroup was of patients with R3-indeterminate mammograms [74.9 %]. PPV for recall in this subgroup is low at 9.5 % which drops to 4.4 % for the prevalent screen. Biopsy rate was 25.3 % and prevalent screeners were more likely to have benign biopsies.

Specificity was high for R4/R5 scores with combined PPV of 76.7 % which increases to 93.6 % for R5 score. No significant variation within this group was noted between incident and prevalent rounds.

If patient recall was limited to R4/R5 lesions on the prevalent screens, we would have missed 8.6 % of malignancies (47cases). If patient recall was limited to R3-indeterminate and above, our recall rate would have dropped to 7.8 % and potentially 0.4 % of cancers would have been missed (2 cases).

**Conclusion:** Use of risk stratification scoring system can help reduce prevalent recall rate. This may however, have a negative impact on the cancer detection rate.

### P12 How to reduce prevalent recall rate? Identifying mammographic lesions with low Positive Predictive Value (PPV)

#### AA Seth, F Constantinidis, H Dobson

##### West of Scotland Breast Screening Centre, Glasgow, UK

###### **Correspondence:** AA Seth (archana.seth@nhs.net) – West of Scotland Breast Screening Centre, Glasgow, UK

**Aim**: To identify mammographic lesions with low PPV on the prevalent screen to decrease false-positive assessment.

**Methods:** The recall rate for 2013-2014 and assessment data for March 2014 were obtained from the local database. Type of mammographic lesion and final outcome were compared.

**Results**: The prevalent recall rate at our unit is 10.3 %. 270 women were assessed in one month. Symptomatic recalls and recalls for axillary lymph-nodes were excluded (n = 26). Of the remaining 244 cases, 107 were prevalent screeners.

Asymmetric densities accounted for 40 % of the assessment clinic workload (50 % on prevalent screen, 32 % on incident screen), with low PPV of 15.9 %. In the prevalent group, no malignancy was diagnosed.

Well-defined opacities accounted for 25 % of the assessment clinic workload, with a very low PPV of 6.4 % (4 % prevalent round, 8.1 % incident round).

Ill-defined opacities and parenchymal distortion are two subgroups with high PPV of 58 % for malignancy and/or surgical referral. On the prevalent round, PPV is 28.6 % for ill-defined opacities and 25 % for distortions. On the incident round, PPV is 62.9 % for ill-defined opacities and 75 % for distortions.

Micro-calcifications accounted for 15.6 % of the cases recalled. 86.8 % of these were biopsied, with PPV of 28.9 % for malignancy (no significant difference between the incident and prevalent rounds).

**Conclusion**: Well-defined opacities and asymmetric densities are mammographic findings with low PPV for malignancy. It is feasible to reduce the prevalent recall rate at our centre by not recalling these mammographic lesions, without any detrimental effect on the cancer detection rate.

### P13 Behaviour of untreated pulmonary thrombus in oncology patients diagnosed with incidental pulmonary embolism on CT

#### R. Bradley^1^, G. Bozas^2^, G. Avery^2^, A. Stephens^2^, A. Maraveyas^2^

##### ^1^North Lincolnshire & Goole NHS Foundation Trust, Scunthorpe, UK; ^2^Hull and East Yorkshire Hospitals NHS Trust, Kingston upon Hull, UK

###### **Correspondence:** R. Bradley (robert.bradley1@nhs.net) – North Lincolnshire & Goole NHS Foundation Trust, Scunthorpe, UK

**Aim:** Identifying a clinically unsuspected pulmonary embolism (UPE) on CT is a common occurrence in oncology patients. This study evaluates the behaviour of untreated UPE by reviewing the change in imaging appearances with time.

**Materials and methods:** Between March 2010 and March 2012, 108 oncology patients had clinically UPE identified on CT chest imaging. Retrospective review was performed; looking for pre-existing unreported pulmonary thrombus on prior CT, comparing changes in imaging appearances.

**Results:** 91 patients with incidental UPE had relevant previous CT chest imaging. The prevalence of pre-existing pulmonary thrombi was 13 % (n = 12). In two (17 %) of these patients pre-existing thrombi were located in the lobar arteries and in 10 (83 %) in segmental and sub-segmental branches.

Comparison of the distribution of UPE in baseline and previous imaging showed that in six (50 %) cases it had remained similar for a median of 92 days (range: 63–253), in three (25 %) there was progressive extent of thrombosis (scan intervals: 84, 174, 193 days respectively) and in three (25 %) distributions of thrombi were different (scan intervals: 51,232, 547 days respectively).

**Conclusions:** Untreated UPE in oncology patients does not always progress in the immediate term, with the majority in this cohort remaining static. Resolution and variable vascular distribution suggests that pulmonary thrombus can develop locally rather than as the consequence of a thromboembolic event.

### P14 A one-stop lymphoma biopsy service – is it possible?

#### S Bhuva, CA Johnson, M Subesinghe, N Taylor

##### Oxford University Hospitals NHS Foundation Trust, Oxford, UK

###### **Correspondence:** S Bhuva (shaheel.bhuva@ouh.nhs.uk) – Oxford University Hospitals NHS Foundation Trust, Oxford, UK

**Aims**: To evaluate the feasibility of a one-stop lymph node core biopsy service based on recent experience.

**Methods**: Retrospective review of all patients referred by haematology to a single radiologist for ultrasound guided core biopsy over 17 months. Data on turnaround time, prior imaging, indication, biopsy site and histological outcome recorded.

**Results**: 74 consecutive haematology referrals directly to a specific radiologist for nodal biopsy from November 2014 to April 2016. Steady increase in referrals over the 17 months. Ultrasound guided 14G core biopsy used in virtually all cases; 14G × 5 cores for ‘new diagnoses’ and 14G × 3 for ‘relapse/transformation’.

53/74 patients (71 %) were biopsied within a week of referral, 69/74 patients (93 %) biopsied within two weeks. Most 62/74 patients (84 %) had relevant diagnostic imaging prior to biopsy. 41/74 (55 %) patients referred as a ‘new diagnosis’ and 33/74 (46 %) for ‘relapse/transformation’. Biopsy sites: axilla > cervical > inguinal nodes.

**Histological outcomes**: 58 malignant and 16 benign. 53 haematological malignancies including 2 patients having an excision biopsy for confirmation (both T cell lymphomas). 5 non-haematological malignancies. 16 benign, of which 5 underwent another test/repeat biopsy: 3 upgraded to malignant. Overall, >95 % diagnostic on the first US guided core biopsy.

**Conclusion**: US guided core biopsy provides an effective diagnosis of haematological malignancy. Radiological considerations for a one-stop service include: providing either ad hoc ultrasound slots or a dedicated list; number of radiologists to support the service; considering coordinating biopsy slots with CT staging appointments; and anticipating referrals from other MDMs should the service show promise.

### P15 Changes in the new TNM classification for lung cancer (8^th^ edition, effective January 2017)

#### LE Quint, RM Reddy, GP Kalemkerian

##### University of Michigan Health System. Ann Arbor, Michigan, USA

###### **Correspondence:** LE Quint (lequint@umich.edu) – University of Michigan Health System. Ann Arbor, Michigan, USA

**So Learning objectives**

The International Association for the Study of Lung Cancer recently proposed changes to the TNM Classification for Lung Cancer in order to better align with patient prognosis. The changes will be incorporated into the 8^th^ edition classification system, taking effect in January 2017. The object of this poster is to describe these changes and illustrate them with imaging examples.

**Content organisation:**

*T stage changes:*

Greater emphasis on primary tumour size, recognising the correlation between size and prognosis. Lower T subcategories will be designated using 1 cm size increments, and larger tumours will be shifted into higher T subcategories.

T1a ≤ 1 cm

T1b > 1 – 2 cm

T1c > 2 – 3 cm

T2a > 3 – 4 cm

T2b > 4 – 5 cm

T3 > 5 – 7 cm

T4 > 7 cm

T2 category will include:

- Involvement of the main bronchus, regardless of distance from the carina (without carinal involvement).
- Associated post-obstructive atelectasis/pneumonitis that extends to the hilar region, involving either part or the entire lung.

T4 category will include diaphragmatic invasion.

*N stage changes:* none.

*M stage changes:*

- M1b Single extrathoracic metastasis in a single organ.
- M1c Multiple extrathoracic metastases in one or several organs.

*Stage Groupings changes:*

- New subcategories in Stages I, II and III based on T1 subcategories.
- New stage IIIC including higher T stage tumours (T3, T4) and N3 nodal involvement.
- New subcategories in Stage IV based on M1 subcategory.

**Conclusion:**

Radiologists need to be familiar with the staging changes in order to be informed and integral members of multidisciplinary patient care teams.

### P16 Cancer immunotherapy: a review of adequate imaging assessment

#### G González Zapico, E Gainza Jauregui, R Álvarez Francisco, S Ibáñez Alonso, I Tavera Bahillo, L Múgica Álvarez

##### Hospital Universitario Cruces, Bizkaia, Spain

###### **Correspondence:** G González Zapico (ggzapico@gmail.com) – Hospital Universitario Cruces, Bizkaia, Spain

**Learning objectives**: To review the response evaluation of immunotherapeutic agents with the immune – related Response Criteria (irRC), advances made since their implementation and potential imaging pitfalls that derive from their differences.

**Content organisation:** Recent developments in anti-cancer therapies had created the need to think of new response evaluation techniques, as past criteria did not explain nor completely correlate with all clinical tumour responses.

We would like to show an iconografic review of irRC applied to our patients, including new developments and progress published to date since their creation in 2009.

We will remember the potential pitfalls in imaging evaluation of these rather new therapies and how to manage them.

**Conclusions:** Imaging rests as one of the most important evaluation pillars for all anti-cancer therapies, and the use of irRC helps monitoring new types of response that correlate better with clinical responses that result from the use of these new immune agents, avoiding that way potential pitfalls that arise from the use of prior classic response criteria.

### P17 Succinate dehydrogenase mutations and their associated tumours

#### O Francies, R Wheeler, L Childs, A Adams, A Sahdev

##### Barts Health NHS Trust, London, UK

###### **Correspondence:** O Francies (olivia.francies@bartshealth.nhs.uk) – Barts Health NHS Trust, London, UK

**Learning objectives:** To review the protean tumour manifestations of succinate dehydrogenase (SDH) gene complex mutations through multi-modality imaging techniques.

**Content organisation:** Mutations in the SDH gene complex are linked to an increased incidence of a spectrum of tumours, namely paragangliomas, and recently demonstrated strong links to other tumours including gastrointestinal stromal tumours (GISTs) and renal cell carcinomas. These mutations and their associated syndromes have been documented in the last 15 years but the process of tumourigenesis is still incompletely understood and the phenotypic expression and its penetrance remains unpredictable. Advances in medical genetics have allowed easier and earlier recognition of these mutations, with imaging playing a major role in primary diagnosis, screening and surveillance.

We analysed patients with SDH mutations referred to our institution over the last decade to:

- Review the genetic basis of SDH mutations, including the current theories behind tumourigenesis.
- Highlight the variety of clinical presentations that lead to a specialist and tertiary centre referral.
- Present a multi-modality imaging review of the variety of tumours and their distribution within each SDH mutation, highlighting any differences between sporadic and syndrome-related tumours.
- Explain surgical and medical management challenges that are encountered when tumours manifest.
- Present our current screening and surveillance programmes for these patients.

**Conclusions:** SDH mutations are associated with an early and life-long risk of tumour development. The spectrum of tumours and their currently unpredictable pattern of disease present complex challenges for clinical teams, and Radiologists play a vital and increasingly significant role.

### P18 Initial experience in the usefulness of dual energy technique in the abdomen

#### SE De Luca, ME Casalini Vañek, MD Pascuzzi, T Gillanders, PM Ramos, EP Eyheremendy

##### Deustches Hospital, Buenos Aires, Argentina

###### **Correspondence:** T Gillanders (tatigillanders@gmail.com) – Deustches Hospital, Buenos Aires, Argentina

**Learning objectives:** The purpose of this review is to describe advantages of Dual Energy technique in tumour detection and lesion characterisation.

**Content organisation:** Dual energy is an innovative imaging technique that has been described to have a considerable effect on the care of oncological patients. It operates applying two different energy settings that makes it possible to differentiate materials with different molecular compositions on the basis of their attenuation profiles.

We will describe the physics behind Dual Energy CT technique and the reconstruction techniques including iodine maps, virtual unenhanced and monochrome virtual images. We will review 65 cases of patients in our institution performed with a 320-row detector CT scanner (Aquilion One, Toshiba medical systems, Otawara, Japan), between January 2014 and January 2016.

We will discuss the following pathologies:

- Hepatocellular carcinoma.
- Primary renal and pancreatic malignant tumours.
- Hypervascular tumour metastasis.

**Conclusion:** Dual energy is an innovative imaging technique that has a considerable effect on the care of oncologic patients. In 10 % of our patients, included in our initial database, we found that this tool improved lesion detection in hypervascular masses. Therefore helping define a more accurate oncological staging in these patients. Dual Energy improved lesion conspicuity in all patients with hepatocellular carcinoma, including one patient whose lesion was only visible in the iodine map. In addition surgical planning was modified in 3 %.

### P19 Recognising the serious complication of Richter’s transformation in CLL patients

#### C Stove, M Digby

##### Stobhill Hospital, 133 Balornock Rd, Glasgow G21 3UW, UK

###### **Correspondence:** C Stove (c.stove.10@aberdeen.ac.uk) – Stobhill Hospital, 133 Balornock Rd, Glasgow G21 3UW, UK

**Learning objectives:** To review the clinical and radiological features of a Richter’s Transformation in Chronic Lymphocytic Leukaemia[CLL].

**Content organisation:** In patients with CLL a Richter’s transformation to a diffuse large B cell Lymphoma is a serious complication occurring in 5-10 % of patients. Even with treatment patient’s median survival is only approximately 6 months. Therefore it is very important the radiologist is aware of this condition which is the purpose of review.

We present the common clinical symptoms and signs associated with the condition.

Also we discuss the common radiological findings of this condition with supporting clinical examples from our institution. In particular we describe how rapidly enlarging lymphadenopathy and extranodal disease in CLL patients should alert the radiologist to a different pathology such as a Richter’s Transformation.

The role of percutaneous biopsy to confirm the diagnosis and further imaging with PET-CT will also be described.

**Conclusion**: The purpose of this review is to describe the clinical and radiological features of the serious complication of a Richter’s transformation in CLL patients.

### P20 Body diffusion-weighted MRI in oncologic practice: truths, tricks and tips

#### M. Nazar, M. Wirtz, MD. Pascuzzi, F. Troncoso, F. Saguier, EP. Eyheremendy

##### Deutsches Hospital, Buenos Aires, Argentina

###### **Correspondence:** M. Wirtz (melinawirtz@yahoo.com) – Deutsches Hospital, Buenos Aires, Argentina

**Learning objectives:** Describe the basics concepts of diffusion-weighted imaging (DWI) and list the common indications in oncology and applications of a DWI sequences. Discuss DW MR changes in response to therapy and how this can be assessed. We also include common tips and tricks to optimise quality and to avoid common mistakes in oncology practice.

**Content organisation:** We will present the spectrum of tumours imaging and discuss:

- **MRI techniques:** In addition to axial acquired DWI (*b* value*:* 0, 200, 400 and 800), conventional T1w, T2w and STIR images were usually obtained in axial and coronal planes.
- **Clinical applications for oncology DW-MRI**: Lesion detection, characterisation, and response assessment.
- **Image interpretation:** disease is identified as areas of impeded diffusion, however DWI findings are not specific for malignancy because other cellular processes, such as inflammation can result in similar findings.

We will review:

- False - positive results.
- False - negative results.

**Conclusion:** DWI can be clinically useful for tumour detection, lesion characterisation, and therapy response. A correct technique and knowledge of potential interpretative pitfalls will help to avoid mistakes.

### P21 Methotrexate-induced leukoencephalopathy in paediatric ALL Patients

#### D.J. Quint, L. Dang, M. Carlson, S. Leber, F. Silverstein

##### University of Michigan Medical Center, Ann Arbor, Michigan, USA

###### **Correspondence:** D.J. Quint (djquint@umich.edu) – University of Michigan Medical Center, Ann Arbor, Michigan, USA

**Learning objectives:** In paediatric patients with Acute Lymphoblastic Leukemia (ALL) being treated with intrathecal methotrexate, we will review the:

- prevalence and breadth of subacute neurologic symptoms.
- MRI findings with special emphasis on diffusion MR.
- differential diagnosis of MRI findings.
- expected clinical course.

**Content organization:** ALL is a common paediatric malignancy and a common cause of non-traumatic paediatric mortality. Methotrexate (MTX) is one of the key therapeutic agents utilised for treatment of these patients.

MTX is well-known to be neurotoxic with/without associated radiation therapy.

Neurotoxicity related to MTX administration may be manifest clinically in the acute/subacute period as seizures, confusion and/or focal neurologic deficits similar to other pathologic processes that these patients are at risk for developing including stroke, opportunistic infection, hemorrhage, venous sinus thrombosis and/or spread of neoplasm.

MRI is often performed in these patients to assess for abnormality. While the majority of MR imaging sequences are usually normal in patients with MTX-related neurotoxicity, supratentorial white matter diffusion MR findings have been identified in an often reversible pattern that appears to correlate with symptomatology.

Examples of children with reversible toxic leukoencephalopathy related to MTX administration will be reviewed. Proposed underlying pathophysiologic mechanisms for the observed MRI findings will also be reviewed.

**Conclusion:** Leukoencephalopathy in ALL patients following IT MTX administration should not be confused with other more ominous pathologic processes that can also effect these patients. Interpreters of paediatric neuroimaging studies should be aware of these MR findings and their implications.

### P22 Pitfalls in oncology CT reporting. A pictorial review

#### R Rueben, S Viswanathan

##### Royal Infirmary, Glasgow, UK

###### **Correspondence:** R Rueben (raymondrueben@gmail.com) – Royal Infirmary, Glasgow, UK

**Learning objectives**: To demonstrate reporting errors, where normal anatomy or variants have been described as pathology.

**Content organisation:** Computed tomography (CT) is the mainstay of oncology diagnosis and follow up. With increasing number in reliance of CT studies, the aim of this pictorial review is to highlight common and uncommon pitfalls that occur during reporting secondary to normal variants and/or technical artefacts.

We aim to present organ specific examples and how to identify and avoid unnecessary further investigations or under/over calling. These would include: ­Lung ­Pleura

- Vascular structures.
- Solid abdominal organs.
- Peritoneum and soft tissue.

It is inevitable that there will be occasions when errors are made. However some errors may drastically alter a patient’s management plan and these individuals may be over or under treated.

**Conclusions:** With increased reliance on CT for oncological diagnosis and follow up it is imperative to that errors in reporting are minimised. It is however impossible to avoid all errors. Hence, we hope this pictorial review will raise ones’ awareness on some of the areas that may be prone to discrepancy in order to reduce unnecessary errors in oncological reports.

### P23 Imaging of perineural extension in head and neck tumours

#### B Nazir, TH Teo, JB Khoo

##### National Cancer Centre, Singapore, Singapore

###### **Correspondence:** B Nazir (bab_nazir@hotmail.com) – National Cancer Centre, Singapore

**Learning objective**: To present anatomical and patho-physiological perspective of “Perineural extension” in Head and Neck Tumours, its clinical implications and highlight the role of imaging through comprehensive pictorial essay of representative cases.

**Content organisation:** Nerves provide easy conduit for spread of malignant cells in head and neck cancers. This involvement of nerves, known as “Perineural extension”, is often clinically occult. However, its presence up grades the tumour staging, alters treatment planning and portends poorer prognosis. Imaging plays pivotal role in the assessment and precise delineation of perineural extension.

We will review the:

- Importance of perineural extension in surgical treatment and radiotherapy planning.
- Clinical presentation of perineural extension.
- Anatomy of cranial nerve distribution and inter-neural connections in head and neck region. Patho-physiological mechanism of nerve involvement in cancer.

We will discuss the:

- Imaging planning and protocols for optimal detection of perineural extension.
- Imaging features on CT, MRI and PET scan in perineural extension. We will present the:
- Pictorial examples of spectrum of representative cases of perineural extension.
- Differentia diagnosis and imaging pitfalls in perineural extension.

**Conclusion**: Perineural extension in head and neck cancer influences the prognosis. It has important implications on the surgical and radiation treatment planning. Knowledge of pattern of perineural extension and the imaging features is essential for proper multidisciplinary management of patients with head and neck and tumours.

### P24 MRI findings of molecular subtypes of breast cancer: a pictorial primer

#### K Sharma, N Gupta, B Mathew, T Jeyakumar, K Harkins

##### St. Vincent’s Medical Center, Bridgeport, Connecticut, USA

###### **Correspondence:** K Sharma (drkomal_sharma@yahoo.in) –St. Vincent’s Medical Center, Bridgeport, Connecticut, USA

**Learning objectives:** Provide an overview of molecular classification of breast cancer. To review the imaging features of the individual subtypes of breast cancer based on recent studies.

**Content organisation:** Breast carcinoma is the most common malignancy affecting women in the United States. Traditionally breast cancer is classified based on the morphologic features. In addition predictive biologic markers including estrogen, progesterone receptors, Her-2/neu receptor status and Ki-67 can be used to subclassify breast cancer into following intrinsic subtype:1. Luminal A 2. Luminal B 3. Her-2/neu or ERBB2 4. Basal.

Several recent studies have evaluated MRI features associated with different intrinsic subtype of breast cancers. For example, ERBB2 and luminal B subtypes have been noted to have increased incidence of multicentric disease, multifocal disease, skin-nipple-periareolar involvement, and axillary disease than luminal A. Preopertaive MRI in Her-2/neu and luminal B, may thus be helpful to better define the extent of disease for surgical planning and optimal local treatment.

Our presentation will be a pictorial essay demonstrating the MRI features of some of the intrinsic subtypes of breast cancer using examples of the cases performed at our women’s imaging center.

**Conclusions:** Based on recent studies MRI features may be used to predict molecular subtypes of breast cancer. However, currently data is limited and further studies are needed to establish the role of pre-operative MRI based on molecular subtypes.

### P25 When cancer can’t wait! A pictorial review of oncological emergencies

#### K Sharma, B Mathew, N Gupta, T Jeyakumar, S Joshua

##### St. Vincent’s Medical Center, Bridgeport, Connecticut, USA

###### **Correspondence:** K Sharma (drkomal_sharma@yahoo.in) – St. Vincent’s Medical Center, Bridgeport, Connecticut, USA

**Learning objectives:** Provide a pictorial review of imaging findings of the common and uncommon emergencies seen specifically in patients with malignancy.

**Content organisation:** Cancers is a leading cause of morbidity and mortality worldwide, with reported 14 million new cases and 8.2 million cancer related deaths in year 2012. Newer improving cancer therapies have resulted in overall increasing prevalence of cancer despite significant cancer related mortality. Acute presentation of a patient to ED may be a result of the primary disease, effect of treatment or unrelated to the diagnosis of cancer. The oncological emergencies are sub classified as metabolic, hematologic and structural conditions. Imaging plays a key role in identifying some of the structural abnormalities such as cord compression or superior vena cava syndrome or treatment complication.

We present a pictorial review of the common and uncommon oncological emergencies seen in radiology department in emergency setting. The focus will be on the conditions that have characteristic imaging findings. The imaging case examples will include cases such as superior vena cava syndrome, acute cord compression, massive pulmonary embolism, chemotherapy related bowel perforation, intestinal obstruction secondary to bowel mass and cerebral herniation among others.

**Conclusions:** Oncological emergencies are uncommon cause of mortality in cancer patients. However, with increasing cancer prevalence radiologist should be familiar with the imaging features of the specific conditions in this subgroup of patient presenting to emergency department.

### P26 MRI of pancreatic neuroendocrine tumours: an approach to interpretation

#### D Christodoulou, S Gourtsoyianni, A Jacques, N Griffin, V Goh

##### Guy’s and St Thomas’ Hospitals NHS Foundation Trust, London, UK

###### **Correspondence:** D Christodoulou (dimitra1@doctors.org.uk) – Guy’s and St Thomas’ Hospitals NHS Foundation Trust

**Learning objectives:** To describe the MRI sequences used in the detection of pancreatic neuroendocrine tumours (pNETs). To demonstrate the typical MRI features of pNETs and highlight the atypical features with reference to histologically proven cases.

**Content organisation:** PNETs may occur sporadically or in association with syndromes such as multiple endocrine neoplasia and von Hippel Lindau disease. Imaging detection can be challenging, particularly when lesions are small. Early diagnosis is crucial to avoid metastatic disease but may be difficult in cases of sporadic nonsecreting tumours. We demonstrate a range of MRI images to illustrate the variety of appearances that may be seen.

**Conclusion:** Being aware of the less common MRI appearances of pNETs may encourage earlier consideration of a neuroendocrine tumour, particularly in patients without a history of a familial syndrome.

### P27 Gynaecological cancers in pregnancy: a review of imaging

#### CA Johnson, J Lee

##### Oxford University Hospitals NHS Foundation Trust, London, UK

###### **Correspondence:** CA Johnson (c.a.yoong@gmail.com) – Oxford University Hospitals NHS Foundation Trust, London, UK

**Learning objectives**: To review the imaging findings of gynaecological cancers in pregnancy; including the unusual as well as the common presentations of malignancy in addition to conditions that may mimic cancers.

**Content organisation:** Gynaecological cancers in pregnancy are well recognised and cause great concern for the patient and her healthcare professionals, as any treatment may also affect the developing foetus and reproductive tract. Management is influenced by the tumour site and stage and nodal staging- all of which are performed with non-ionising radiation. Ultrasound, especially TV ultrasound, and MR are the most common imaging modalities.

We will present the most common gynaecological malignancies in pregnancy, their typical findings; specific effects that drive growth in pregnancy and typical appearances on radiological imaging. In addition, we will also describe lesions that cause concern in pregnancy, may grow in size and have adverse appearances suggesting malignancy but are actually benign on histology.

Imaging is all the more important in pregnancy as biochemical markers such as Ca-125 and BHCG are already raised in pregnancy.

**Conclusions**: Gynaecological cancers in pregnancy cause great concern and challenges to the Oncological Radiologist. The pertinent imaging findings are included in this pictorial review.

### P28 Suspected paraneoplastic neurological syndromes - review of published recommendations to date, with proposed guideline/flowchart

#### JA Goodfellow, AS Al-adhami, S Viswanathan

##### Glasgow Royal Infirmary, 84 Castle St, Glasgow G4 0ET, UK

###### **Correspondence:** AS Al-adhami (abdullahaladhami@gmail.com) – Glasgow Royal Infirmary, 84 Castle St, Glasgow G4 0ET, UK

**Learning objectives:** To review the recommendations for diagnosis and investigation of suspected Paraneoplastic Neurological Syndromes in the literature, and propose a guideline.

**Content organisation:** Paraneoplastic Neurological Syndromes (PNS) almost always predates the detection of malignancy, and prompt diagnosis increases likelihood of a favorable neurologic outcome. However, true PNS are rare with even sub-specialty neurologists often only seeing a handful of cases a year. Moreover, there are many different PNS which span multiple neurological subspecialties, each with different associated tumours.

We will discuss the different categories of PNS and their diagnostic criteria.

We will also review the published recommendations on investigations for suspected PNS, and follow-up for definite PNS.

Lastly, we will propose a flowchart which summarises these recommendations. This flowchart has been adopted by the Glasgow’s Institute of Neurological Sciences (the tertiary neurological centre for the west of Scotland). It is intended to help make the published recommendations more accessible to referring clinicians. In addition, it provides a valuable guide for reporting radiologists on what the associated tumours are to help focus their reporting.

**Conclusion**: Clinicians requesting investigations for suspected PNS should have an understanding of the different types of PNS, and detail specific syndrome suspected in the imaging request. This will provide a more focused imaging pathway. A simplified flowcharted would aid achieving this for both the referring clinician and the reporting radiologists.

### P29 Multi-parametric MRI of the pelvis for suspected local recurrence of prostate cancer after radical prostatectomy

#### R Bradley (robert.bradley1@nhs.net)

##### North Lincolnshire & Goole NHS Foundation Trust, Scunthorpe, UK

**Learning objectives:** To review the role of multi-parametric MRI (mpMRI) in detection of suspected locally recurrent prostate cancer when biochemical failure occurs after radical prostatectomy.

**Content organisation:** Rising prostate-specific antigen (PSA) levels after radical prostatectomy may be an indicator of locally recurrent prostate cancer. Recurrent disease occurs in up to 50 % of high risk patients and 10 % of low risk patients within 15 years of surgery. Rising PSA levels, known as biochemical failure, can be due to local recurrence and/or metastatic disease. Persistently raised PSA after prostatectomy may be due to residual healthy glandular tissue in the post-surgical bed.

I will review the current role of multi-parametric MRI (mpMRI) in the detection of locally recurrent prostate cancer, including:

- Biochemical failure after radical prostatectomy.
- Indications and protocols for imaging.
- Implications of imaging findings for further management.

I will discuss the imaging characteristics of post-prostatectomy changes in the pelvis on mpMRI:

- Appearances of local tumour recurrence.
- Enhancement kinetics in differentiation of benign scarring from tumour recurrence.
- Correlate mpMRI appearances of recurrent tumour with histological findings.

**Conclusions:** Multi-parametric MRI is a useful tool in confirming the diagnosis of locally recurrent prostate cancer after radical prostatectomy in patients with biochemical failure. Combined imaging parameters assist in the differentiation of recurrent tumour from scarring and residual healthy tissue. Imaging findings can be used to guide further management such as salvage radiotherapy.

### P30 Utilisation of PI-RADS version 2 in multi-parametric MRI of the prostate; 12-months experience

#### R Bradley (robert.bradley1@nhs.net)

##### North Lincolnshire & Goole NHS Foundation Trust, UK

**Learning objectives**: To demonstrate the use of the PI-RADS (Prostate Imaging-Reporting and Data System) version 2 (v2) scoring system for multi-parametric MRI (mpMRI) of the prostate, with imaging examples correlated with histological findings.

**Content organisation:** mpMRI of the prostate gland is increasingly being performed to assess the treatment naïve prostate gland prior to performing biopsy in suspected prostate cancer. Advantages include tumour detection and localisation of suspected clinically important lesions, facilitating individualised biopsy patterns for patients, and providing an opportunity for staging without post-biopsy artefact. MRI may also have a role in risk stratification potentially deferring biopsy in some cases.

I will review the current PI-RADS v2 with imaging examples from our own experience over the last 12 months in approximately 800 patients.

I will discuss the changes in scoring of lesions compared to PI-RADS version 1(v1) with imaging examples.

I will present our local Prostate Cancer pathway and how mpMRI and PI-RADSv2 has been incorporated into this.

**Conclusions: PI-RADS provides a** standardised approach to reporting lesions detected on mpMRI of the prostate, with version 2 simplifying the overall scoring system. Widespread incorporation into prostate cancer pathways is needed to understand how best to apply mpMRI findings to individual patients. Continuing experience will provide an opportunity to investigate whether PI-RADS 3 indeterminate lesions could be further characterised into those needing biopsy and those that could be safely followed.

### P31 Radiological assessment of the post-chemotherapy liver

#### A Yong, S Jenkins, G Joseph

##### Velindre Cancer Centre, Cardiff, UK

###### **Correspondence:** A Yong (audrey.yong@wales.nhs.uk) – Velindre Cancer Centre, Cardiff, UK

**Learning objectives:**

1. To illustrate the patterns of hepatic parenchymal change/injury following chemotherapy.
2. To differentiate benign changes in response to chemotherapy from malignant disease.
3. To recognize response vs progression in patients undergoing treatment with anti-angiogenesis agents.

**Content organisation:** Chemotherapy-induced hepatic toxicity is a well recognised complication. Certain chemotherapy regimes increase the likelihood of hepatic injury. The recognition of toxicity is particularly important in patients being considered for liver resection. MRI has a role in problem-solving when the CT appearances are difficult to interpret.

We will present the more commonly encountered parenchymal changes (steatosis, steatohepatitis, nodular regenerative hyperplasia, sinusoidal obstructive syndrome), demonstrating the typical appearances on CT and/or MRI.

The patterns of parenchymal change are different in patients who have undergone long-term – and multiple – lines of chemotherapy – these changes will also be demonstrated on CT and/or MRI.

Tumour response to newer anti-angiogenesis chemotherapy agents differs from standard chemotherapy regimes – recognition of ‘pseudo-progression’ and the role of MRI in problem-solving will be highlighted.

**Conclusion:** Chemotherapy- induced hepatic toxicity is a commonly encountered complication, the features of which should be recognised during standard follow-up reporting.

It is important to differentiate response from progression with newer anti-angiogenesis chemotherapy agents.

MRI has a role in problem-solving when hepatic parenchymal and response assessment is suboptimal on CT.

### P32 Skeletal staging with MRI in breast cancer – what the radiologist needs to know

#### S Bhuva, K Partington

##### Oxford University Hospitals NHS Foundation Trust, Oxford, UK

###### **Correspondence:** S Bhuva (shaheel.bhuva@ouh.nhs.uk) – Oxford University Hospitals NHS Foundation Trust, Oxford, UK

**Learning objectives:** To identify normal, benign and metastatic skeletal appearances on MRI.

- To understand that processes, such as marrow conversion, reduce sensitivity.
- To identify complications of metastatic disease and extra-skeletal findings.

**Content organisation:** Breast cancer commonly metastasizes to the axial skeleton. Our local practice is to use a MRI marrow examination using T1WI and STIR sagittal thoracic spine and coronal lumbar-pelvis sequences.

We offer a pictorial review of key features that the radiologist needs to know when reporting MRI marrow examinations for breast cancer (re-)staging. This will include:

- Illustrations of normal appearances.
- Challenges posed by marrow conversion in reducing sensitivity for detecting metastases.
- Review areas and localisers.
- Typical appearances of bone metastases: focal and diffuse infiltration.
- Post treatment changes on MRI and the utility of the CT body study to diagnose a healing response.
- Complications of malignant vertebral collapse and cord compression.
- Benign processes that may be confused for metastatic disease, such as insufficiency fractures, Modic changes, Paget’s disease and benign lesions (eg Pitt’s pit).
- Extra-skeletal findings relevant to the assessment of metastatic burden and/or breast cancer treatment include adenopathy, pleural and pulmonary lesions, ascites, hydronephrosis and pelvic lesions (eg endometrial masses following tamoxifen).

**Conclusion**: MRI is an excellent tool for assessing the skeleton for metastases. Recognising benign and malignant appearances, appreciating processes that limit interpretation, identifying potential complications and an awareness of extra-skeletal findings will allow for an accurate report.

### P33 Perineural spread of lympoma: an educational review of an unusual distribution of disease

#### CA Johnson, S Bhuva, M Subesinghe, N Taylor

##### Oxford University Hospitals NHS Foundation Trust, London, UK

###### **Correspondence:** CA Johnson (c.a.yoong@gmail.com) – Oxford University Hospitals NHS Foundation Trust, London, UK

**Learning objectives**:

- Perineural spread of disease is a rare yet potentially devastating manifestation of lymphoma, that may occur in isolation without other sites of nodal or extra nodal disease.
- CT, MR and PET-CT have an important role in the assessment of perineural spread of disease.

**Content organisation:** Perineural spread of disease is most commonly encountered in the head and neck along branching cranial nerves. However, in lymphoma, perineural involvement can be seen almost anywhere in the body. It most often presents as a painful sensorimotor neuropathy but presentation is variable and dependent upon neural structures involved.

Any linear focus of abnormal FDG uptake especially in the distributions of major nerves without overt structural correlate raises suspicion for perineurial involvement on FDG PET-CT. Retrospective evaluation of previous imaging studies, such as contrast-enhanced MR, can then performed in a “second look” evaluation.

Direct signs of perineural spread include increased prominence of the nerve; with thickening and irregularity and avid enhancement. Indirect signs include muscle weakness and atrophy suggesting denervation. Indirect signs confirmed by CT are bony erosion, destruction and widening of exiting nerve foramina.

**Conclusions:** Perineural spread of disease in lymphoma is less common than the typical nodal masses and splenomegaly. Perineural spread of disease has acute and chronic sequelae. It has a poor prognosis and changes management of disease.

### P34 Visually isoattenuating pancreatic adenocarcinoma. Diagnostic imaging tools

#### C Carrera, A Zanfardini, S De Luca, L Alarcón, V Blanchet, EP Eyheremendy

##### Hospital Alemán. Buenos Aires, Argentina

###### **Correspondence:** S De Luca (sdeluca@hospitalaleman.com) – Hospital Alemán. Buenos Aires, Argentina

**Aim**: To characterise the imaging findings of visually isoattenuating pancreatic adenocarcinoma in MDCT, their secondary signs and their correlation with MRI and US.

**Content organisation:** We reviewed MDTC performed in 85 patients with a suspected pancreatic mass between September 2009 and May 2016. We obtained in all the cases multiplanar dynamic dual-phase scan, and MIP reformatted images. Isoattenuating tumours were found in 10 of them, all histologically confirmed at surgery (n = 7) or biopsy (n = 3). These cases were diagnosed by MRI in 40 %, US in 30 %, both modalities in 20 % and only CT in 10 %. Retrospectively we found one or more subtle secondary signs on MDCT in all of them.

**Summary**: Isoattenuating pancreatic adenocarcinoma are a small but meaningful percentage of pancreatic cancer (5,4 %).

They were histopathology more common moderately differentiated (70 %) than well differentiated (10 %) and poorly differentiated (20 %).

Secondary signs, such as mass effect and/or convex contour abnormality, loss of the normal acinar glandular pattern, dilated biliary and pancreatic ducts, interrupted pancreatic duct, and atrophic distal pancreatic parenchyma, are helpful in the diagnosis.

Although US may be a sensitive method in the detection of these tumours in cases with adequate glandular visualisation; MRI can improve the diagnosis providing better tissue characterisation, especially with diffusion sequence and ADC map.

### P35 Imaging of larynx cancer: when is CT, MRI or FDG PET/CT the best test?

#### K Cavanagh, E Lau

##### Peter MacCallum Cancer Centre, Melbourne, Australia

###### **Correspondence:** K Cavanagh (kardacav@gmail.com) – Peter MacCallum Cancer Centre, Melbourne, Australia

**Learning objectives:** To review Computed Tomography (CT), Magnetic Resonance Imaging (MRI) and ^18^Fluorodeoxyglucose Positron Emission Tomography/ Computed Tomography (PET/CT) in the diagnosis of larynx cancer with an emphasis on optimal imaging modality to elucidate the features of larynx cancer pertinent to staging.

**Content organisation:** CT is the most commonly performed imaging modality. It is fast, relatively inexpensive and widely available. Tailored technique is required to ensure adequate visualisation of larynx cancer and its relationship to associated anatomical planes. Limitations include ability to detect small and mucosal lesions and relative low sensitivity for cartilage invasion.

MRI demonstrates superior soft tissue definition and accurate local disease extent. Protocols need to be tailored for larynx cancer. MRI enables superior visualisation of laryngeal cartilage invasion, pre and para-glottic space invasion to confirm T3 and T4 disease with high sensitivity and moderate specificity. Limitations include relative increased cost, movement artefacts, longer acquisition times, patient compatibility.

PET/CT main indication is detection of cervical adenopathy (reported up to 20 % more accurate than CT or MRI), exclusion of metastatic disease and synchronous primary. Limitations include highest cost, physiological uptake and movement artefacts.

We will discuss the strengths and weakness of each modality and imaging techniques to maximise larynx lesion conspicuity in CT, MRI and PET/CT.

**Conclusions:** Accurate staging is essential in the diagnosis and treatment of larynx cancer. A good understanding of the imaging modalities, their purpose and optimisation enables accurate characterisation of larynx cancer.

